# Intraskeletal Pathway of the Trigeminal Nerve in Modern Bird Beak: 3D Comparative Anatomy Using the diceCT Method

**DOI:** 10.1002/jmor.70188

**Published:** 2026-10-28

**Authors:** Noriyuki Wakimizu, Takanobu Tsuihiji

**Affiliations:** ^1^ Abiko City Museum of Birds Abiko Chiba Japan; ^2^ Department of Paleontology and Anthropology National Museum of Nature and Science Tsukuba Ibaraki Japan; ^3^ Department of Earth and Planetary Science, Graduate School of Science The University of Tokyo Bunkyo‐ku Tokyo Japan

**Keywords:** Aves, beak, Crocodylia, diceCT, neurovascular canal, skull, trigeminal nerve

## Abstract

The morphology of the trigeminal nerve extending through the snout region in Archosauria has recently been the focus of extensive research, particularly in paleontology. However, the pathways of this nerve within cranial bones in extant birds have not been described in detail. Here, we describe trigeminal nerve pathways in the skull of nine avian species using a non‐destructive soft‐tissue imaging technique (diceCT) with a particular emphasis on osteological correlates, including intraskeletal neurovascular canals as well as foramina and grooves on the bony surfaces. In many species, a branch of the trigeminal nerve, the ophthalmic nerve, extends through the anterior premaxillary canal. Similarly, another branch of the trigeminal nerve, the maxillary nerve, passes through a bony canal in the maxillopalatine process at the posterior end of the snout in many species. However, apparently clade‐specific unique pathways are also observed for the ophthalmic nerve around the external naris in Falconiformes and Psittaciformes and the maxillary nerve on the palatal surface in Anseriformes, the latter of which is likely associated with the specialized tactile sensation. Based on comparison with the condition in Crocodylia, the “reptilian‐like” course of the maxillary nerve within the premaxilla is considered to have apomorphically been lost, whereas that of the ophthalmic nerve within the premaxilla has become extremely prominent in Aves. These changes in the intraskeletal nerve pathways in Aves have likely been associated with the enlargement of the premaxilla and the loss of teeth in the avialan lineage. The present observation of the trigeminal nerve pathway in Aves potentially contributes to the discussion of the homology of other associated soft‐tissue structures and the presence of special sensory functions in fossil archosaurs.

## Graphical abstract

We investigated the intraskeletal pathway of the trigeminal nerve in Aves, using diffusible iodine‐enhanced contrast‐enhanced computer tomography. Among the examined species, the main nerve pathways of the ophthalmic, maxillary, and mandibular nerves were highly conserved, although the ophthalmic nerve pathway around the external naris differed in Anseriformes, Falconiformes, and Psittaciformes. These findings may reflect differences in the degree of skull ossification or their eating habits.

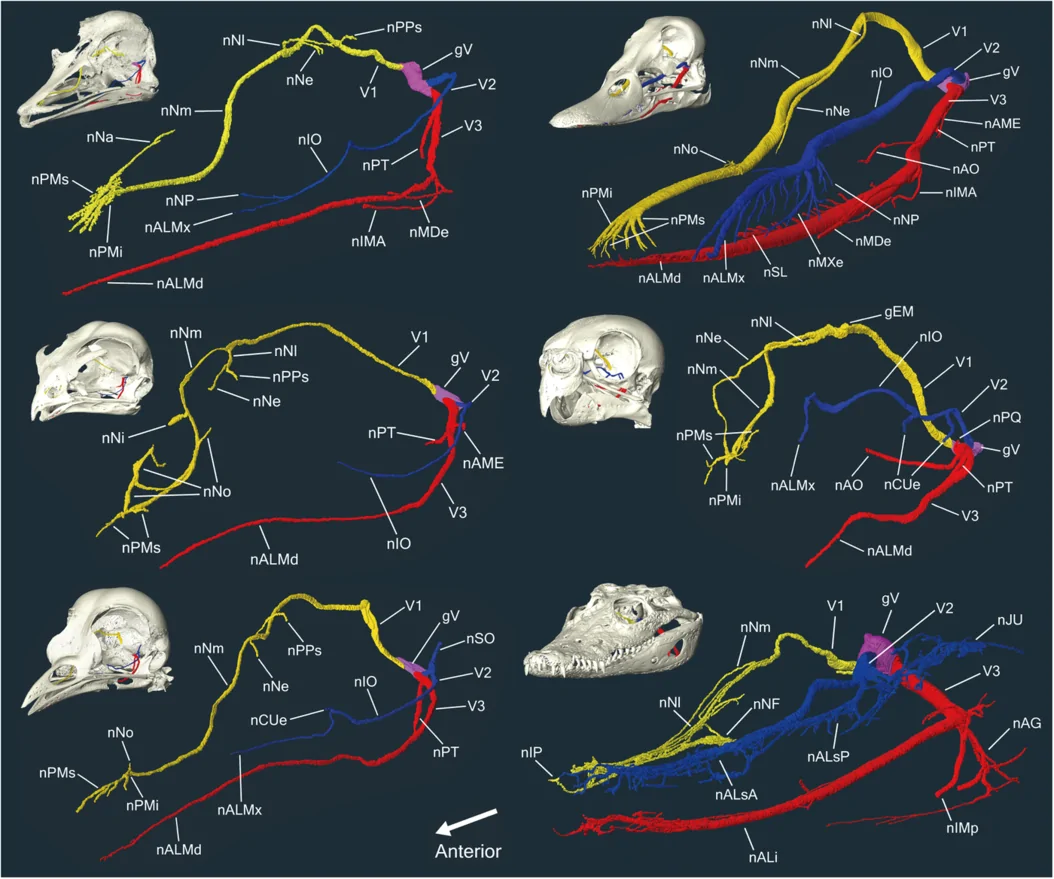

## 1 Introduction

The snout is a functionally important region of the vertebrate body, playing key roles in foraging, olfaction, respiration, and thermoregulation (e.g., Brugmann et al. 2006; Müller et al. 2018). Within Archosauria, snout morphology shows remarkable diversity, varying extensively in length, curvature, and dorsoventral depth (Brusatte et al. 2010; Foth and Rauhut 2013; Bestwick et al. 2018; Hüppi et al. 2021). In addition, edentulous keratinous beaks have evolved independently in several archosaurian lineages, including pterosaurs, ornithischians, oviraptorosaurian theropods, and avialans (Lautenschlager et al. 2013; Meredith et al. 2014; Wang et al. 2017). Numerous hypotheses have been proposed to explain the diversification of snout morphology, citing factors such as diet, foraging behavior, ornamentation, cranial kinesis, and skeletal weight reduction associated with powered flight (Davit‐Béal et al. 2009; Louchart and Viriot 2011; Felice and Goswami 2018; Tattersall et al. 2017; Hone et al. 2020). However, the relative contributions of these factors to the evolution of archosaurian snout morphology remain poorly understood.

Among these factors, we focus here on the snout sensory function, which is mediated primarily by the trigeminal nerve (cranial nerve V; CNV). The trigeminal nerve consists of three major branches: the ophthalmic (V_1_) and maxillary (V_2_) nerves, which innervate the upper jaw, and the mandibular nerve (V_3_), which innervates the lower jaw. Whereas the mandibular nerve contains both sensory and motor branches, the ophthalmic and maxillary nerves are sensory nerves that transmit information from the skin, oral epithelium, and tooth roots (e.g., Dubbeldam 1998; Butler and Hodos 2005). Among extant archosaurs, crocodylians possess hypertrophied trigeminal nerve branches that innervate integumentary sensory organs (ISOs), enabling the detection of mechanical, thermal, and osmotic stimuli (Leitch and Catania 2012; Di‐Poï and Milinkovitch 2013). Birds also possess highly developed sensory organs concentrated at the tip of the beak (Breward and Gentle 1985; Wild 2015; Soliman and Madkour 2017), which support object manipulation in parrots (e.g., Gutiérrez‐Ibáñez et al. 2010; Demery et al. 2011) and the detection of prey through vibrations transmitted through water, soil, and other kinds of sediment in tactile‐foraging birds, including kiwis, ibises, sandpipers, and ducks (e.g., Martin and Piersma 2009; Leitch and Catania 2012; Cunningham et al. 2013).

The specialization of trigeminally innervated sensory structures in extant archosaurs is likely associated with the evolution of snout morphology and vice versa. Evaluating this relationship across Archosauria, including fossil taxa, requires comparative data on the courses of the trigeminal nerve branches that innervate these sensory structures. In particular, branches that pass through or alongside the jaw bones and leave osteological correlates of their pathways are important (e.g., Bellairs and Jenkin 1960; Walker 1972).

At the anterior part of the braincase, the ophthalmic nerve and maxillomandibular ganglion pass through two or three cranial foramina (Goller 1972; Konig et al. 2016). More anteriorly, trigeminal nerve branches may course alongside, or penetrate, cranial bones, leaving grooves, foramina (neurovascular foramina), and enclosed canals (neurovascular canals) as osteological correlates (*sensu* Witmer 1995). These osteological correlates can be identified not only in extant species but also in fossil taxa through direct observations of skeletal material (e.g., Soares 2002; Brusatte et al. 2012) and computed tomography (e.g., Brazaitis and Watanabe 2011; Barker et al. 2017).

In recent years, numerous descriptive studies have documented the osteological correlates of the trigeminal nerve in fossil archosaurs (e.g., Ibrahim et al. 2014; Herne et al. 2019; Cau 2020; Bouabdellah et al. 2022). Based on the relationships observed in extant species, these studies have used osteological features, including the number of neurovascular foramina and the complexity of neurovascular canals, to infer the sensory capabilities and lifestyles of fossil taxa (e.g., Du Toit et al. 2020; Lessner 2021; Kawabe and Hattori 2022; Lessner et al. 2023a). However, caution is needed when considering neurovascular foramina and canals as osteological correlates of trigeminal nerve branches and discussing their functional significance. Cranial nerves commonly course together with blood vessels as neurovascular bundles (Sedlmayr 2002; Porter 2015), and these bony features may reflect the pathways of both neural and vascular structures. Therefore, it is necessary to verify that they reliably reflect the distribution of trigeminal nerve branches rather than vasculature alone.

The trigeminal nerve in extant archosaurs has been studied with a main focus on the innervation of the jaw adductor musculature (Barnikol 1954; Wild and Zeigler 1980; Holliday and Witmer 2007) and sensory organs in the snout tip (e.g., Cunningham et al. 2007; Leitch 2013; Soliman and Madkour 2017), based on histological and gross anatomical observations. Although several studies have described the continuous courses of the trigeminal nerve branches from their origins to the peripheral regions (e.g., Webb 1957; Dubbeldam and Karten 1978; Abdel‐Kader and Fathy 2002), few studies have directly mentioned the relationship between these nerve pathways and the corresponding bony foramina and canals (Watanabe and Yasuda 1970; Bubien‐Waluszewka 1981; Crole and Soley 2016).

Recently, knowledge of the intraskeletal pathways of trigeminal nerve branches in extant archosaurs has expanded through the application of diffusible iodine‐based contrast‐enhanced computed tomography (diceCT: Gignac et al. 2016). In Crocodylia, most anatomical studies have been conducted on the American alligator (*Alligator mississippiensis*; George and Holliday 2013; Gignac and Kley 2014; Lessner and Holliday 2020; Cerio and Witmer 2023; Lessner et al. 2022). In Aves, a complete description of the intraskeletal pathways of the trigeminal nerve is currently available only for the rock dove (*Columba livia;* Jones et al. 2019), although the bony pathways associated with the bill‐tip sensory organ have also been described in the gray parrot (*Psittacus erithacus;* Lessner et al. 2023b).

In the present study, we describe the intraskeletal pathways of the trigeminal nerve around the beak in a broad phylogenetic sample of extant birds using diceCT. We then evaluate trigeminal nerve‐related osteological correlates and reconstruct the ancestral condition of the bony trigeminal nerve pathway in Aves to provide an anatomical framework for reconstructing sensory systems in fossil archosaurs.

## 2 Materials and Methods

### 2.1 Examined Specimens

Carcasses of wild or captive birds that had died of natural causes were obtained to represent nine order‐level clades of Aves (Figure 1 and Table S1): ostrich (*Struthio camelus*; Struthioniformes), Indian peafowl (*Pavo cristatus*; Galliformes), mallard (*Anas platyrhynchos*; Anseriformes), rock dove (*Columba livia*; Columbiformes), great crested grebe (*Podiceps cristatus*; Podicipediformes), great cormorant (*Phalacrocorax carbo*; Suliformes), common kestrel (*Falco tinnunculus*; Falconiformes), common parakeet (*Melopsittacus undulatus*; Psittaciformes), and dusky thrush (*Turdus eunomus*; Passeriformes). Two crocodylian species were also examined as the outgroup to Aves (Figure 1 and Table S1): Siamese crocodile (*Crocodylus siamensis*; Crocodylidae) and spectacled caiman (*Caiman crocodilus*; Alligatoridae).

**Figure 1:**
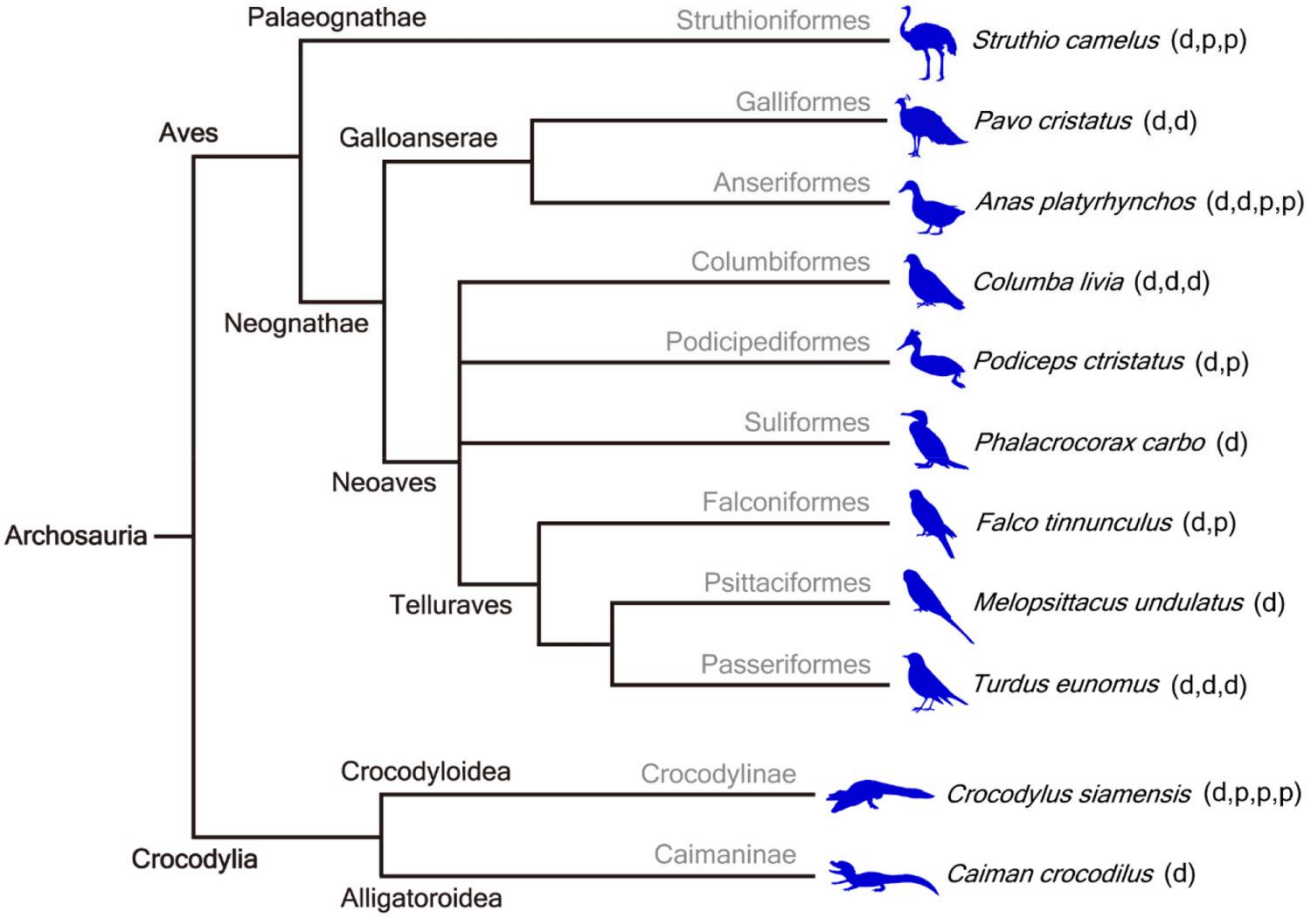
Phylogenetic relationships of all species examined in this study. *Note*. Cladogram is compiled and modified from Prum et al. (2015), Hekkala et al. (2021), and Sangster et al. (2022). Individuals on which both soft tissues and skeletal features were analyzed via diceCT and preCT were indicated with “(d)” following species names, whereas those on which only skeletal features were analyzed via preCT were similarly indicated with “(p).” For *Struthio camelus*, for example, both soft tissues and skeletal features were analyzed in one individual, whereas skeletal features were analyzed in two more individuals. The branch length of this figure is not standardized by time.

### 2.2 Iodine Staining Methods

Heads were separated from the frozen specimens by transection between the first and fifth cervical vertebrae, depending on the specimen size. The isolated heads were fixed in 10% neutral‐buffered formalin (NBF; FUJIFILM Wako Pure Chemical Corporation, Osaka, Japan) for 17–72 days (Table S2). Prior to iodine staining, feathers were removed from the fixed avian specimens to reduce image artifacts caused by the accumulation of contrast agent on the skin surface during CT scanning.

The fixed specimens were stained in 10% (w/v) Lugol's iodine solution (I_2_KI). The staining solution was prepared either by diluting a commercially available 20% Lugol's iodine solution (Muto Pure Chemicals Co. Ltd., Tokyo, Japan) with tap water or mixing 6.7% potassium iodide and 3.3% iodine crystals (FUJIFILM Wako Pure Chemical Corporation, Osaka, Japan) in tap water (Table S2). During staining, the iodine solution was replaced with a fresh solution every 2 weeks.

### 2.3 CT Scan Imaging

Micro‐computed tomography (microCT) scanning was performed using one of three scanners: SkyScan 1173 (BRUKER, Billerica, the United States) at the University of Tokyo; TXS320‐ACTIS (TESCO, Kanagawa, Japan) at the National Museum of Nature and Science, Japan (NMNS); and inspeXio SMX‐225CT FPD HR (SHIMADZU, Kyoto, Japan) at NMNS. During scanning, the specimens were wrapped in moistened polyethylene buffer sheets and sealed in plastic bags to prevent desiccation.

Prior to iodine staining, all specimens were subjected to plain X‐ray CT scanning (preCT) to acquire data on the skeletal morphology of the skull (Table S3). DiceCT scanning was performed on each specimen after at least 17 days of iodine immersion (Table S4). Transverse image series were reconstructed using NRecon version 1.7.4.6 (BRUKER, Billerica, the United States) or VG Studio MAX 2.0 (Volume Graphics, Heidelberg, Germany).

### 2.4 Three‐Dimensional Reconstruction of the Neurovascular Canals and Trigeminal Nerve

Three‐dimensional digital segmentation of the skull and trigeminal nerve pathways was performed using Amira 6.2.0 or Amira 3D Pro version 2021.2 (Thermo Fisher Scientific, Massachusetts, the United States). Owing to hardware limitations (10th generation of Intel Core i9 with 32 GB RAM), CT image stacks were imported into Amira by subsampling every second to fourth slice, resulting in a maximum slice interval of 100 µm.

On the preCT dataset, the skulls were semi‐automatically segmented using the *magic wand* tool, whereas the neurovascular canals and tooth sockets (crocodylians only) within the jawbones were manually segmented using the *brush* tool with the *ambient occlusion* module. On the diceCT datasets, the trigeminal nerve branches were manually segmented using the *brush* tool by tracking each branch from the trigeminal ganglion (Vg) to the tip of the snout through successive transverse sections (x–y plane). The trigeminal nerve branches were carefully traced to avoid including adjacent blood vessels, guided by tissue contrast. For each species, the special relationships between the nerve branches and surrounding anatomical structures (e.g., muscles and skeletal elements) on the diceCT datasets were interpreted using previously published anatomical descriptions of the examined species or closely related taxa (e.g., Webb 1957; Burger 1978; Bout and Dubbeldam 1991; Fjeldså 1993; Carril et al. 2015; Kalyakin 2015; Mahmoud et al. 2017; Jones et al. 2019; Cerio and Witmer 2023).

The segmented skull, neurovascular canals, and trigeminal nerve branches of each specimen were exported separately as Amira surface files using the *generate surface* module. Surface smoothing was applied only to the skull and omitted for the neurovascular canals and trigeminal branches to preserve fine peripheral branching patterns. Because the preCT and diceCT datasets were acquired independently and did not share a common coordinate system, the reconstructed surfaces were manually aligned using the *transform editor* module in Amira. This alignment enabled observations of the special relationship between the trigeminal nerve pathways and cranial osteological features (Figures 2, 3).

**Figure 2:**
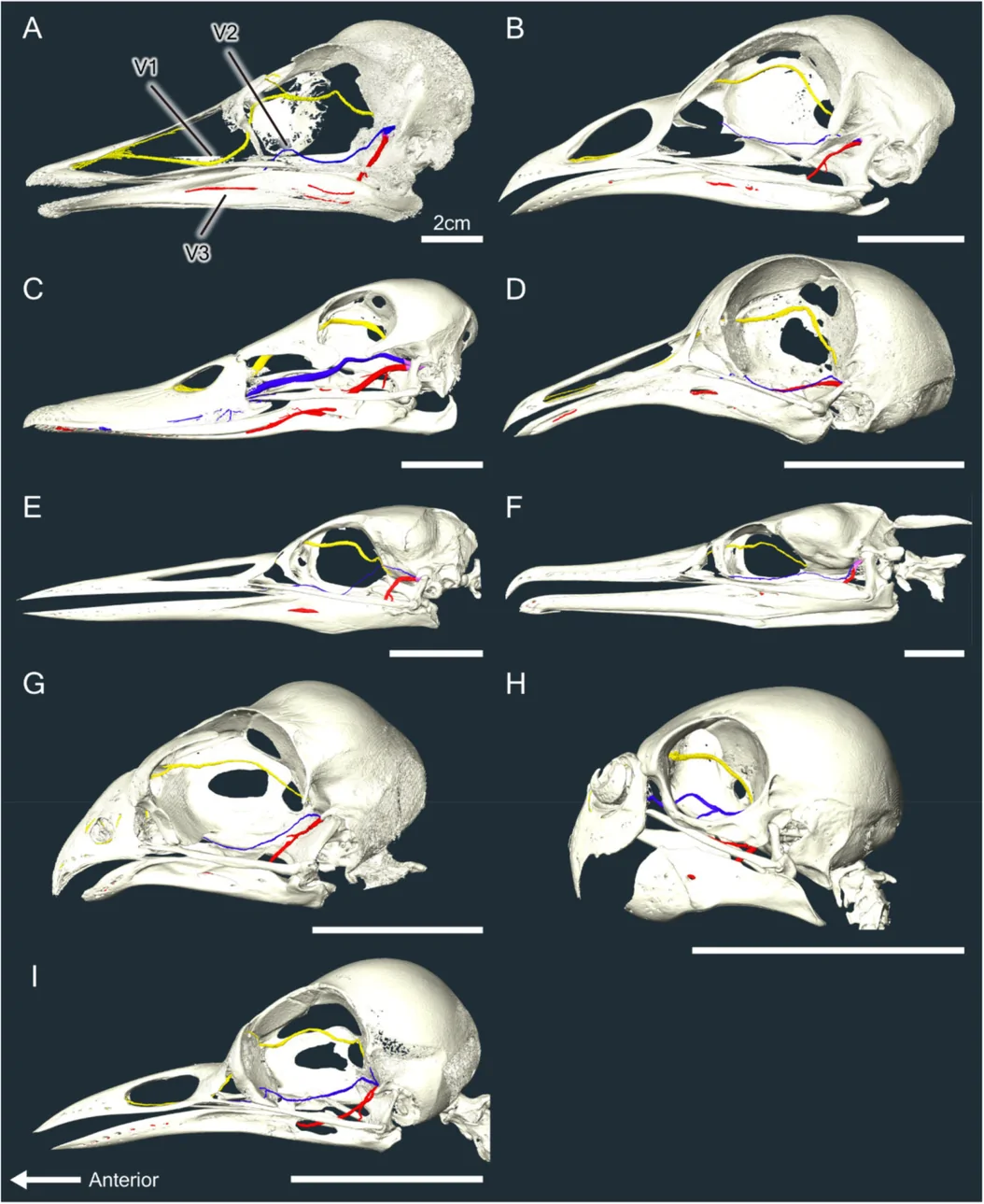
3D reconstruction of the avian trigeminal nerve pathway in left lateral view with solid skull images. (A) *Struthio camelus*. (B) *Pavo cristatus*. (C) *Anas platyrhynchos*. (D) *Columba livia*. (E) *Podiceps cristatus*. (F) *Phalacrocorax carbo*. (G) *Falco tinnunculus*. (H) *Melopsittacus undulatus*. (I) *Turdus eunomus*. The trigeminal ganglion (gV) is in pink, the ophthalmic nerve (V1) is in yellow, the maxillary nerve (V2) is in blue, and the mandibular nerve (V3) is in red. Scale bars: 2 cm.

**Figure 3:**
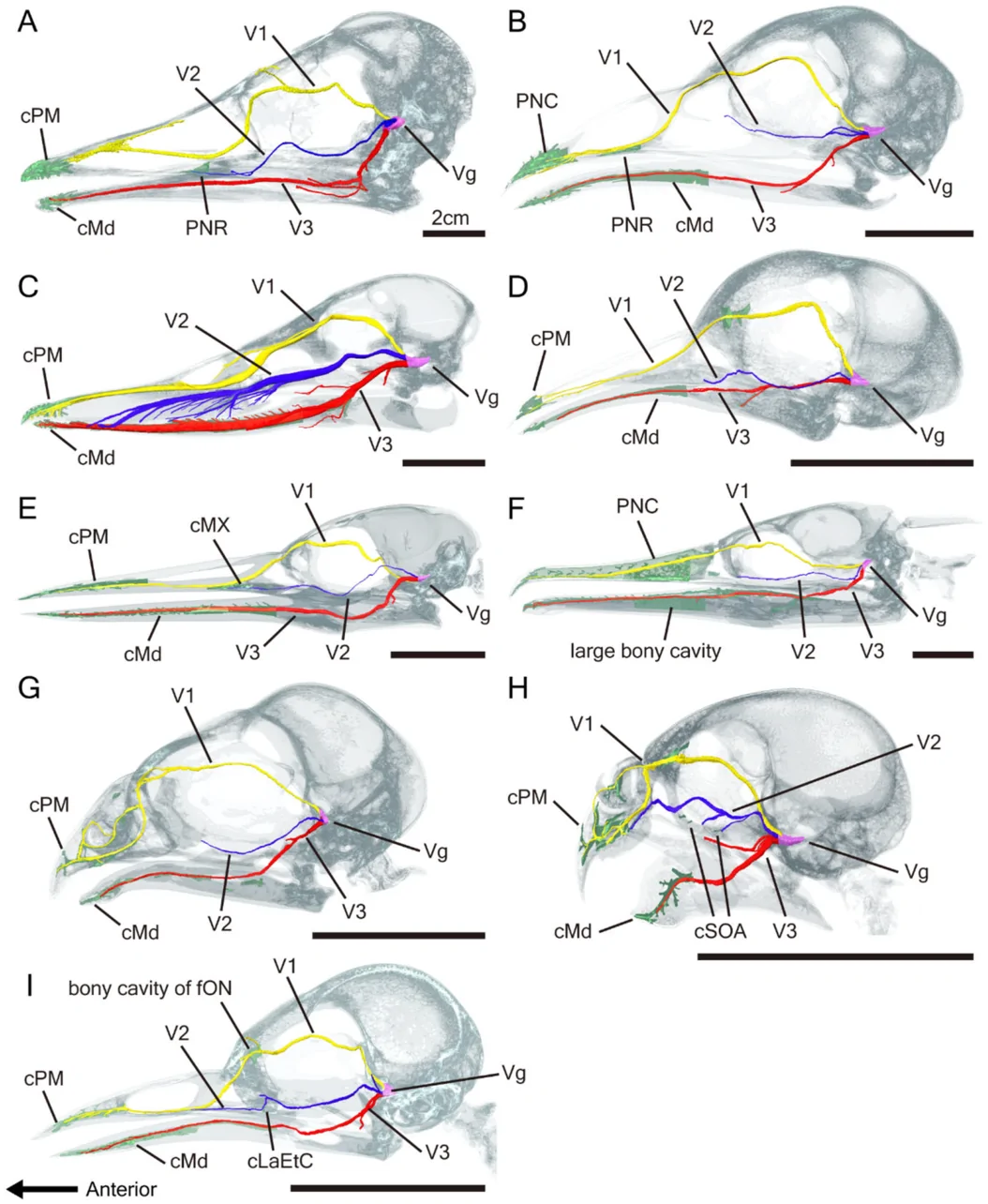
3D reconstruction of the avian trigeminal nerve pathway in left lateral view with transparent skull images. (A) *Struthio camelus*. (B) *Pavo cristatus*. (C) *Anas platyrhynchos*. (D) *Columba livia*. (E) *Podiceps cristatus*. (F) *Phalacrocorax carbo*. (G) *Falco tinnunculus*. (F) *Melopsittacus undulatus*. (I) *Turdus eunomus*. The trigeminal ganglion (gV) is in pink, the ophthalmic nerve (V1) is in yellow, the maxillary nerve (V2) is in blue, and the mandibular nerve (V3) is in red. The neurovascular canal (NVC) and large bony cavities in the skull are shown in light green. cLaEtc, bony canal in the lacrimal–ethmoid bone complex; cMd, mandibular canal; cMX, maxillary canal; cPM, premaxillary canal; cSOA, bony canal in the suborbital arch; fON, orbitonasal foramen; PNC, pneumatic cavity; PNR, pneumatic recess. Scale bars: 2 cm.

## 3 Results

Based on 3D digital data of the trigeminal nerve (Figures 2, 3, 4 and S1–S3), the three main nerve branches (the dorsal ophthalmic, the medial maxillary, and the ventral mandibular nerve) emerge from the cranium posterior to the orbita. These nerve pathways are generally conserved among the examined birds, whereas *A. platyrhynchos* and *M. undulatus* show several distinctive traits. Their anatomical features are described below.

**Figure 4:**
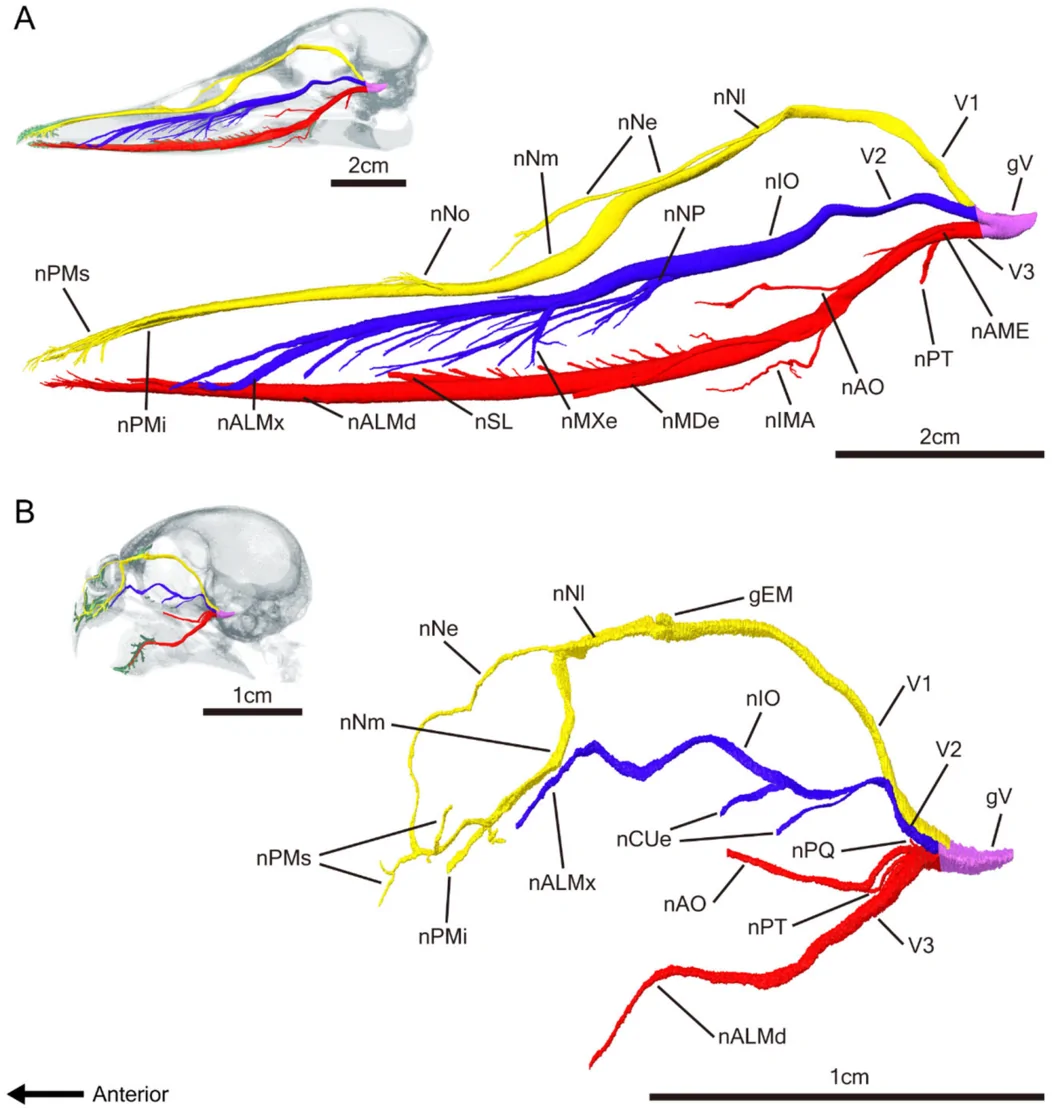
Detailed view of 3D reconstruction of the trigeminal nerve in *Anas platyrhynchos* (A) and *Melopsittacus undulatus* (B) in left lateral view. *Note*. In (A, B), the relationships of the trigeminal nerve course to the skull are shown in the left column. gV, trigeminal ganglion; nALMd, intramandibular nerve; nALMx, maxillary alveolar nerve; nAME, nerve for external adductor muscle of the mandible; nAO, nerve for *anguli oris*; nIMA, intermandibular nerve; nIO, infraorbital nerve; nLCp, lacrimal palpebral nerve; nMDe, external mandibular nerve; nMXe, external maxillary nerve; nNa, nasal branch; nNe, external nasal nerve; nNl, lateral nasal nerve; nNm, medial nasal nerve; nNP, nasopalatine nerve; nPMi, inferior premaxillary nerve; nPMs, superior premaxillary nerve; nPPs, superior palpebral nerve; nPT, pterygoid nerve; nSO, supraorbital nerve; V1, ophthalmic nerve; V2, maxillary nerve; V3, mandibular nerve.

### 3.1 The Ophthalmic Nerve Pathways in Aves

1. **Orbital region**
  Within orbita, the ophthalmic nerve generally extends medially to the eyeball in a convex‐upwards directed trajectory along the interorbital septum before entering the posterodorsal region of the nasal cavity (Figures 2, 3, 4 and S1–S3), as described previously (e.g., Watanabe and Yasuda 1970; Baumel and Witmer 1993). In the posterior orbita, the nerve contacts the lateral surface of the interorbital septum in *S. camelus*, *P. cristatus*, *Po. cristatus*, and *T. eunomus*, although no osteological correlate is present (Figures 7 and S4A).
  The ophthalmic nerve splits into several branches, among which the medial and lateral nasal nerves are most prominent (Figures 4 and S1–S3). The branching position of the lateral nasal nerve (*ramus lateralis nasi*) varies between taxa. In *S. camelus* and *F. tinnunculus*, the nerve emerges in the middle of the orbita (Figures S1A and S3A), whereas in *T. eunomus*, it emerges near the anterior orbital margin and passes through the *foramen orbitonasale mediale* together with the medial nasal nerve (*ramus medialis nasi*, Figures 3 and S4A). In *M. undulatus*, the lateral nasal nerve emerges anterior to this foramen (Figure 6A). In contrast, *A. platyrhynchos* has the most posterior origin among the examined taxa, with the lateral nasal nerve extending anteriorly while contacting the lateral wall of the ethmoidal ridge, a thin longitudinal bony wall on the ventral side of the mesethmoid (Figure 5A–B).
  The lateral nasal nerve subsequently divides into the external nasal nerve (*ramus nasalis externus*) and the superior palpebral nerve (*ramus palpebrales superiores* [Watanabe and Yasuda 1970; Berkhoudt 1980]). In this study, the external nasal nerve is observed in most species, whereas the superior palpebral nerve is observed only in *S. camelus* and *T. eunomus* due to the resolution limitations. No osteological correlates of these nerves are confirmed within the orbita.
2. **From the anterior margin of the orbita to the external naris**
  Within the nasal cavity, the external nasal nerve gives off branches that innervate soft tissues on the posterior margin of the external naris (Watanabe and Yasuda 1970; Berkhoudt 1980). In *A. platyrhynchos*, this nerve is attached to the medial walls of the ectethmoid and nasal bones, while extending anteriorly toward the posterior edge of the external naris (Figure 5C), as described by Berkhoudt (1980). In *F. tinnunculus* and *T. eunomus*, this nerve courses through soft tissues around the dorsolateral wall of the olfactory turbinate. *M. undulatus* differs from these taxa in possessing a bony canal within the mesethmoid (Figure 6B,D), through which the nerve extends before passing through additional canals in the nasal and premaxillary bones toward the posterior midline of the beak (Figure 10A–B).
  The medial nasal nerve generally extends from the anterior orbital tip to the anterior region of the nasal cavity (Watanabe and Yasuda 1970) and descends along the midline elements of the nasal cavity (such as the mesethmoid or cartilaginous nasal septum). In *C. livia*, *F. tinnunculus*, *M. undulatus*, and *T. eunomus*, its course is associated with a bony groove on the mesethmoid (Figures 6C and 7F–G). In contrast, in *Po. cristatus*, the medial nasal nerve pathway is preserved as a shallow groove on the ventral side of the frontal bone (Figure 7A–C). Additionally, nerve‐associated bony canals are confirmed within the mesethmoid of *F. tinnunculus* (Figures S5–S6) and within the maxilla of *M. undulatus* (Figure 6C). In contrast, *A. platyrhynchos* lacks these extensive canals; instead, the nerve follows a shallow depression on the dorsal surface of the maxillopalatine process before passing through the intermaxillary contact posterior to the external naris (Figure 5B–D). *P. carbo* shows a markedly different condition because of the absence of a median nasal septum, with the nerve extending anterolaterally along a large bony cavity rather than curving ventrally toward the palate (Figures 3F and S15I–J).
3. **Bill tip region**
  In the anterior region of the nasal cavity, the medial nasal nerve diverges into the inferior premaxillary nerve (*ramus premaxillaris inferior*) and superior premaxillary nerve (*ramus premaxillaris superior*, Watanabe and Yasuda 1970). In most examined birds, the inferior premaxillary nerve extends beneath the premaxilla before dividing into multiple branches that innervate the soft tissues between the bone and keratinous sheath (Figures 3, 11, and S7A–B). *Anas platyrhynchos* differs in possessing a particularly thick nerve bundle that traverses a deep median groove on the premaxilla before dividing into numerous fan‐shaped branches at the bill tip (Figures 8, 9 and S8) that covers the maxillary cushion (Kooloos et al. 1989, Figure 8A). In *M. undulatus*, the nerve emerges at the premaxilla–palatine joint (Figure 6C) and enters canals within the ventral premaxilla (Figure 6D,F–G). This branch exits anteriorly to innervate the palatal surface (Figure 10E–F).
  The superior premaxillary nerve generally extends into the anterior region of the beak, giving off multiple branches to the outer surface (Watanabe and Yasuda 1970). In most examined taxa, the nerve enters the premaxilla through a foramen near the anterior end of the external naris and branches within the premaxillary canal (Figures 3, 4 and S1–S3). However, *A. platyrhynchos* retains a single trunk almost through the entire premaxillary canal before dividing extensively around the nail and maxillary cushion at the bill tip (Figures 8 and S8). *Falco tinnunculus* shows a posteriorly displaced entrance of the superior premaxillary nerve into the beak through the maxillopalatine process (Figures S5B,C and S6), whereas this nerve in *P. cristatus* divides into branches within a large premaxillary cavity (Figure S7). In *M. undulatus*, the superior premaxillary nerve enters bone through a separate dorsolateral foramen at the premaxilla–palatine joint (Figures 3H, 6C–D, and 10) and joins anteriorly with the external nasal nerve (Figure 10).
  The oral nasal nerve (*ramus nasalis oralis*) has been described as a branch of the superior premaxillary nerve arising near the anterior end of the external naris (Berkhoudt 1980). In *A. platyrhynchos*, it extends anteriorly while giving off branches through fine grooves on the surface of the premaxilla (Figures 5C–D, 8C–E, and S8). *Falco tinnunculus* possesses distinct dorsal and ventral branches extending along the margins of the external naris (Figures S5B and S6C), whereas *M. undulatus* similarly shows a branch extending along the ventrolateral edge of the external naris (Figures 3H, 4B, and 10A–D). In both taxa, the nerve pathways are associated with bony sculptures around the external naris. In *F. tinnunculus*, in addition, the course of the dorsal branch continues toward the ossified nasal turbinate (Figures S5 and S6A–B).

**Figure 5:**
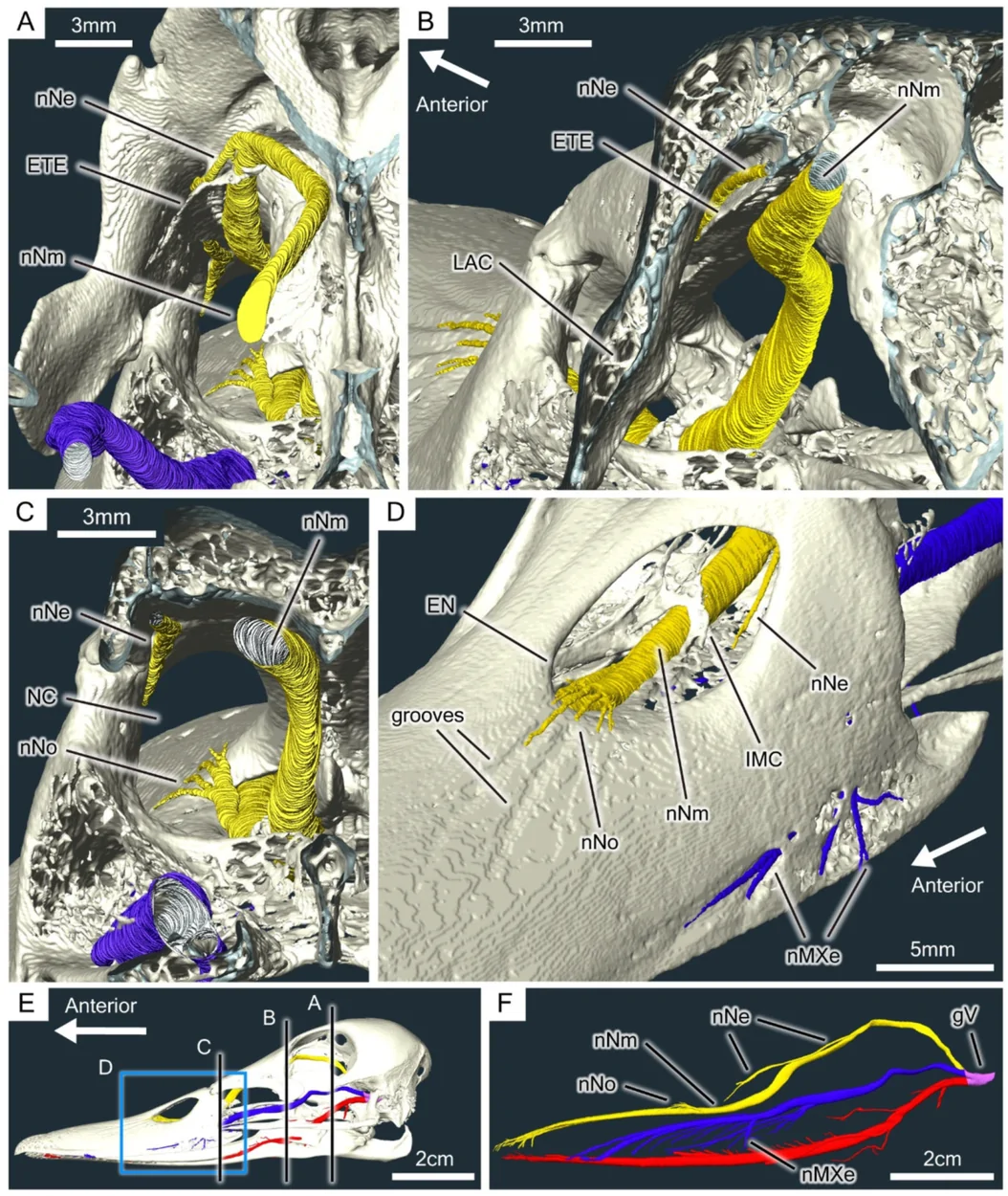
Anterior to middle parts of the ophthalmic and maxillary nerve pathways in *Anas platyrhynchos. Note*. (A) Posterior view of the transverse section in the posterior orbital region. (B) Left posterolateral view of the transverse section in the anterior orbital region. (C) Posterior view of the transverse section in the nasal cavity. (D) Area surrounding the external naris in left anterolateral view. (E) Left lateral view of the skull showing the positions of transverse sections (A–C) and the area shown in (D). (F) Entire course of the trigeminal nerve. EN, external naris; ETE, ethmoidal edge; gV, trigeminal ganglion; IMC, intermaxillary contact; LAC, lacrimal; NC, nasal cavity; nMXe, external maxillary nerve; nNe, external nasal nerve; nNm, medial nasal nerve; nNo, oral nasal branch.

**Figure 6:**
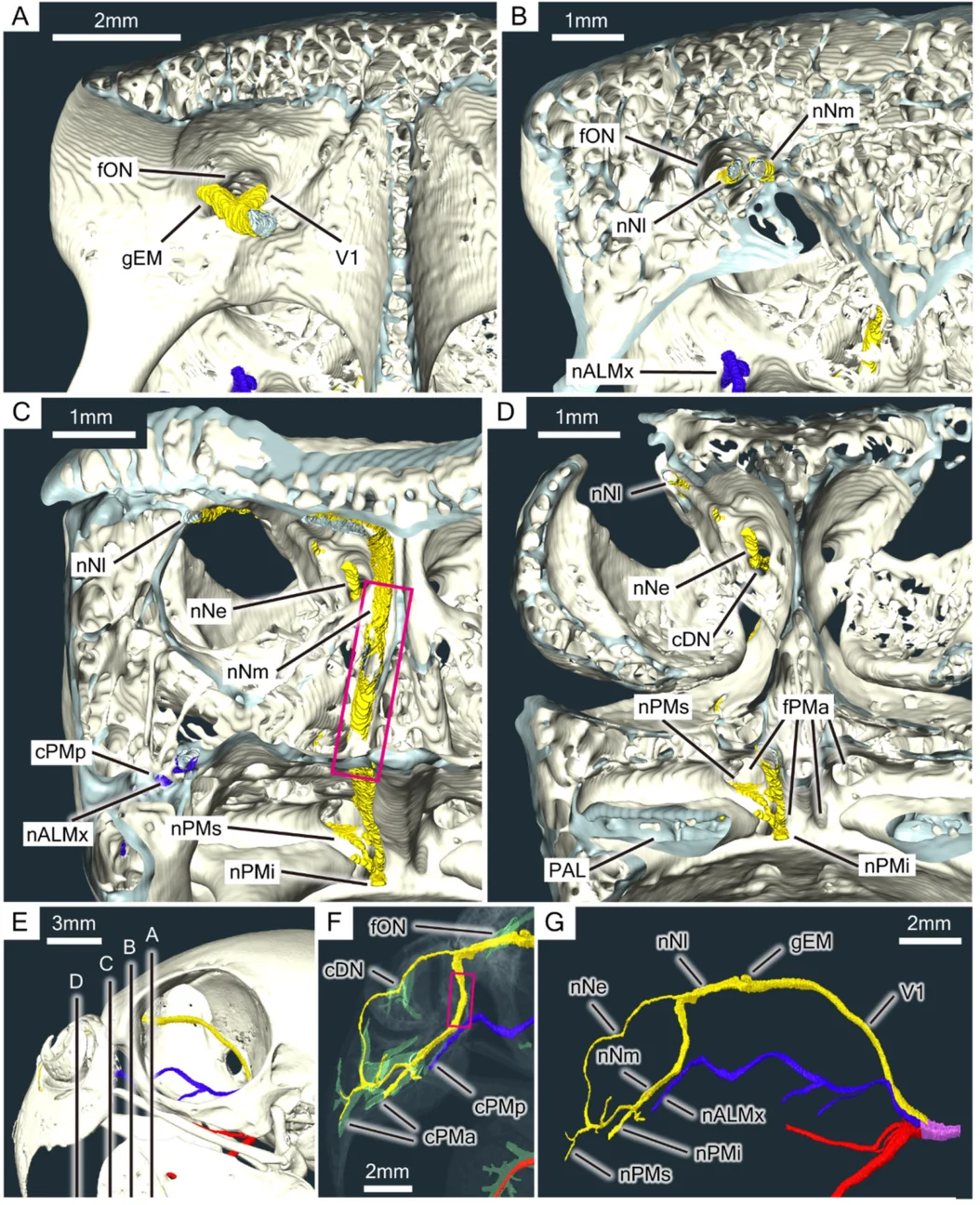
Anterior to middle parts of the ophthalmic and maxillary nerves in *Melopsittacus undulates. Note*. (A–D) Relationship between the nerves and skull on the transverse sections at the positions indicated in (E) in the posterior view. In (C), the area where the medial nasal nerve (nNm) extends within the bone in the midline nasal cavity is shown with a red square. (E) Left lateral view of the skull showing the positions of transverse sections (A–D). (F) Anterior to the middle parts of the ophthalmic and maxillary nerves in a transparent skull in left lateral view. Large bony cavities in the skull are shown in light green. (G) Entire ophthalmic and maxillary nerves in the left lateral view. cDN, dorsonasal canal in mesethmoid; cPMa, anterior part of premaxillary canal; cPMp, posterior part of premaxillary canal; fON, orbitonasal foramen; fPMa, posterior premaxillary foramen connected to the cPMa; gEM, ethmoidal ganglion; nALMx, maxillary alveolar nerve; nNe, external nasal nerve; nNl, lateral nasal nerve; nNm, medial nasal nerve; nPMi, inferior premaxillary nerve; nPMs, superior premaxillary nerve; PAL, palatine; V1, ophthalmic nerve.

**Figure 7:**
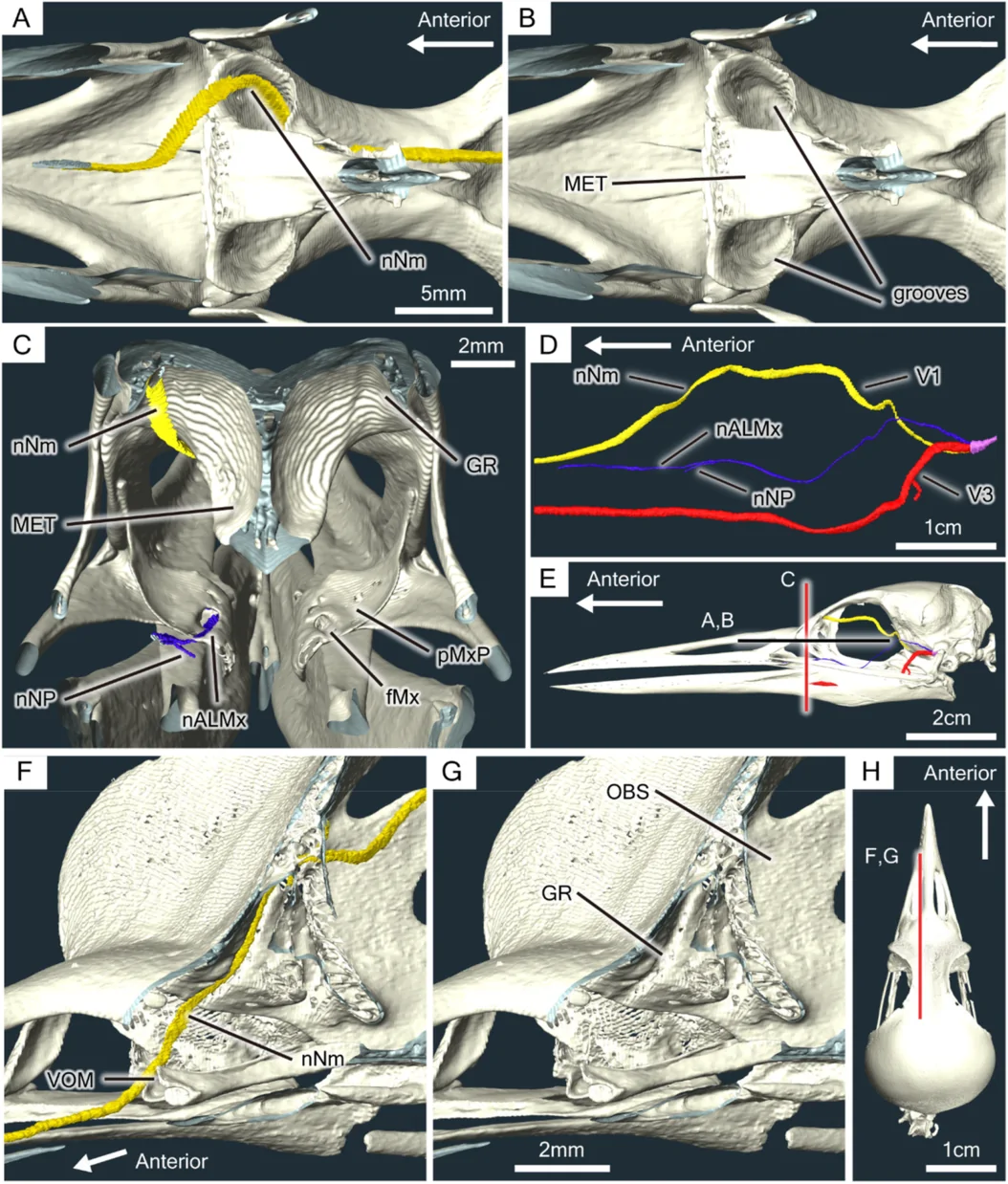
Bony grooves for the medial nasal nerve (nNm) pathway around the nasal cavity. (A–E) *Podiceps cristatus*. (F–H), *Turdus eunomus*. (A) Ventral view of the horizontal section at the level indicated in (E). (B) Same as (A) but without the nerve. (C) Posterior view of the transverse section at the level indicated in (E). (D) The trigeminal nerve in the middle through posterior parts of the head in the left lateral view. (E) Skull and the trigeminal nerve in the left lateral view showing the positions of cross sections (A–C). (D) The anterolateral view of the sagittal section at the level indicated in (H). (G) Same as (F) but without the nerve. (H) The dorsal view of the skull. fMx, maxillary foramen; GR: bony groove; MET, mesethmoid; nALMx, maxillary alveolar nerve; nNP, nasopalatine nerve; OBS, bony orbital septum; pMxP, maxillopalatine process; V1, ophthalmic nerve; V3, mandibular nerve; VOM, vomer.

**Figure 8:**
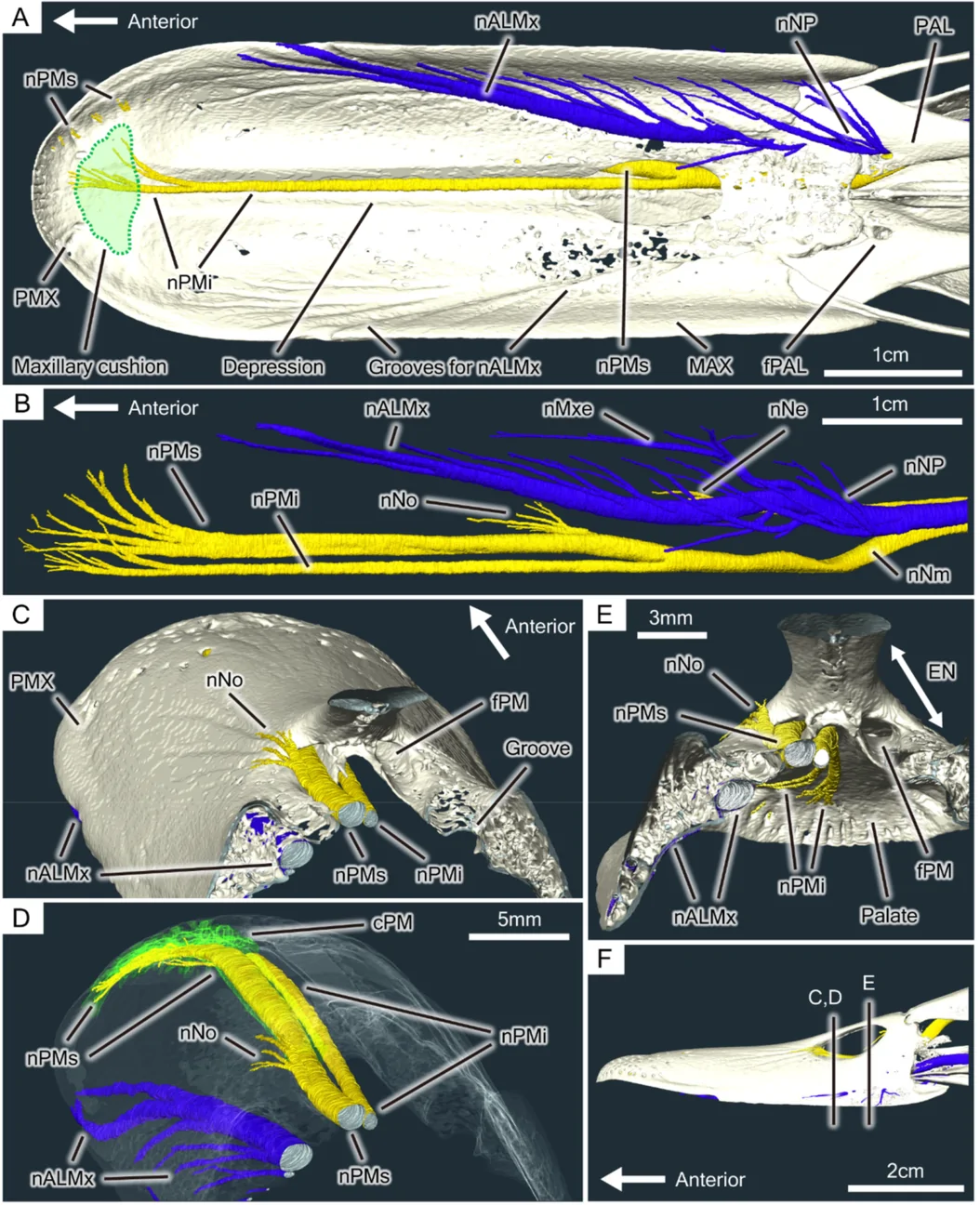
The ophthalmic and maxillary nerve pathway in *Anas platyrhynchos* in the beak. *Note*. (A, B) Ophthalmic and maxillary nerve pathways on the ventral side of the upper beak in the area indicated in (F). In (A), the green area indicates the area of the epidermal maxillary cushion. (C, D) Left posterolateral view of the nerve pathway sectioned at the level indicated in (F) in a solid (C) or transparent (D) skull, respectively. (E) Posterior view of the nerve pathway sectioned at the level indicated in (F). (F) Left lateral view of the skull showing the positions of the cross sections (C–E). cPM, premaxillary canal; EN, external naris; fPAL, palatine foramen; fPM, premaxillary foramen; MAX, maxilla; nALMx, maxillary alveolar nerve; nMXe, external maxillary branch; nNe, external nasal nerve; nNm, medial nasal nerve; nNo, oral nasal branch; nNP, nasopalatine nerve; nPMi, inferior premaxillary nerve; nPMs, superior premaxillary nerve; PAL, palatine; PMX, premaxilla.

**Figure 9:**
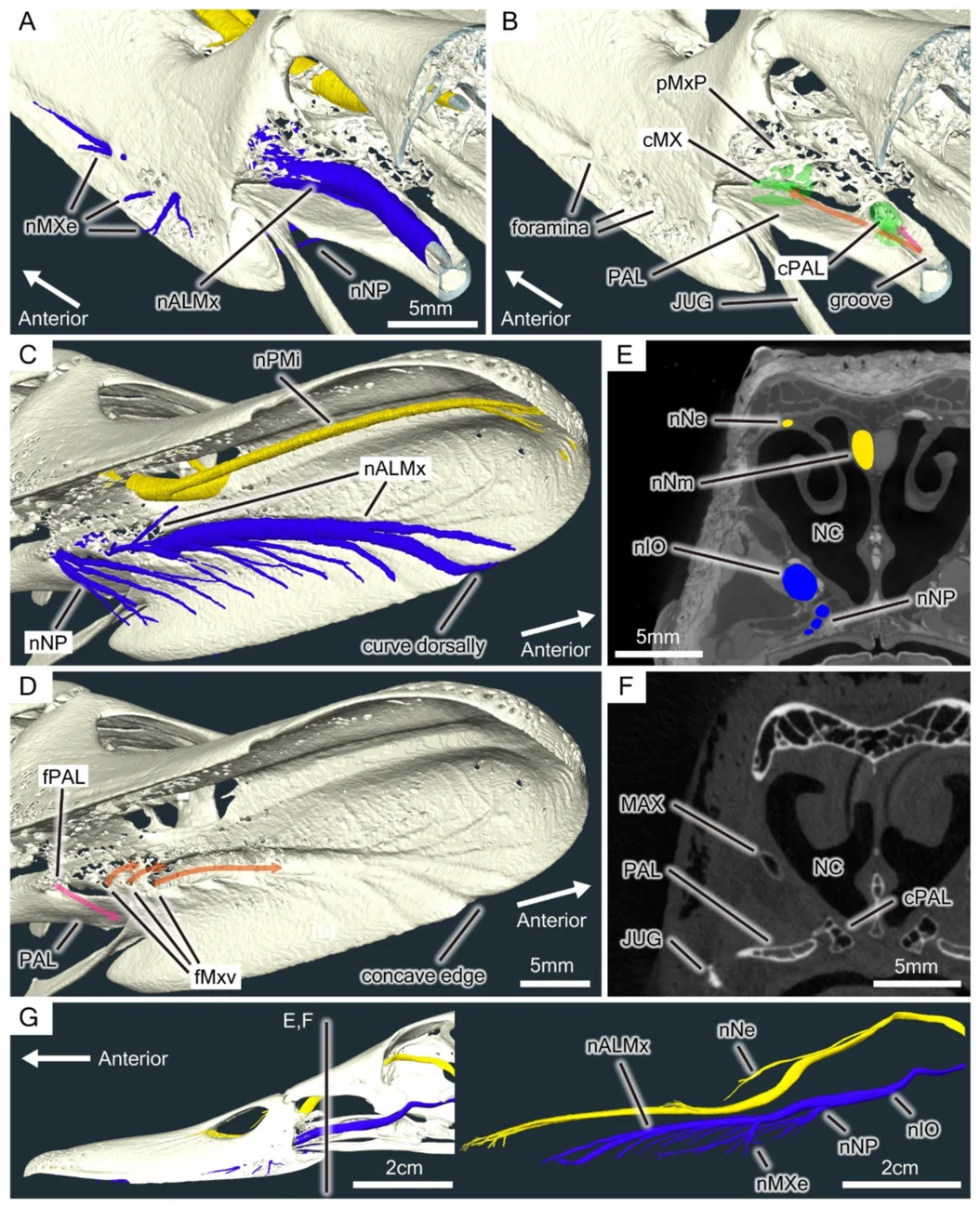
The maxillary nerve pathway at the snout region of *Anas platyrhynchos. Note*. (A–D) Close‐up views of the skull and nerve pathway in the area indicated in (G). (A–B) Left posterolateral view with and without the nerves, respectively. In (B), the bony openings for the maxillary canal (cMX) and palatine canal (cPAL) are indicated in green. (C–D) Right anteroventral view with and without the nerves, respectively. The nerve pathways of the maxillary alveolar nerve (nALMx) and nasopalatine nerve (nNP) are shown in orange and pink lines, respectively, in (B, D). (E, F) Transverse diceCT images at the level indicated in (G). (G) Skull (left) and trigeminal nerve pathway (right), with the position of close‐ups (A–D) and cross sections (E, F) indicated. fMXv, ventral maxillary foramen; fPAL, palatine foramen; JUG, jugal; MAX, maxilla; NC, nasal cavity; nIO, infraorbital nerve; nMXe, external maxillary branch; nNe, external nasal nerve; nPMi, inferior premaxillary nerve; PAL, palatine; pMxP, maxillopalatine process.

**Figure 10:**
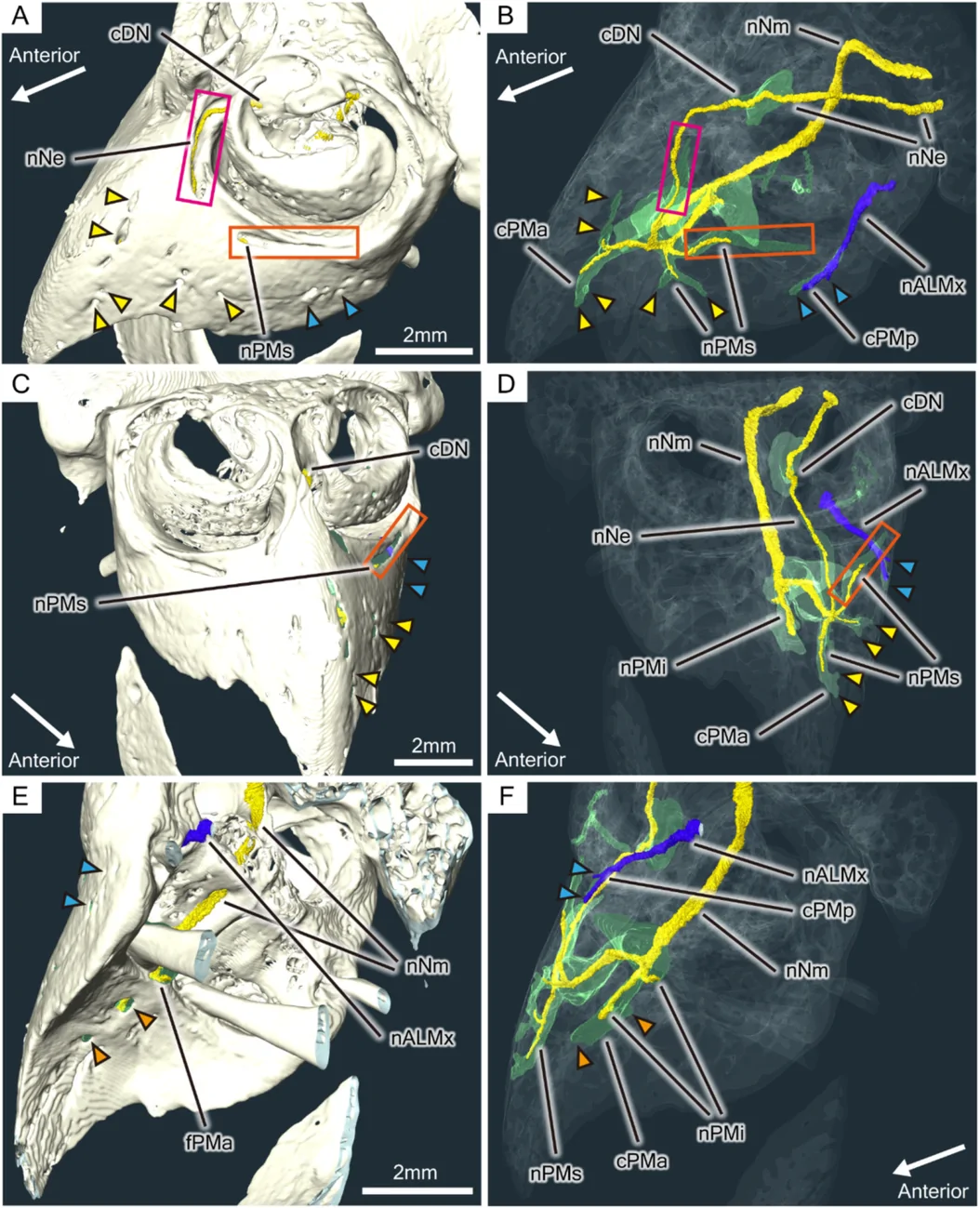
Ophthalmic and maxillary nerve pathways in the snout region of *Melopsittacus undulates. Note*. Nerve pathways are shown with a solid (A, C, E) or transparent (B, D, F) skull. (A, B) Left anterolateral view. (C, D) Right anterolateral view. (E, F) Left posteroventral view. The area where the external nasal nerve (nNe) extends in a bony canal or groove anterior to the external naris is indicated with a red square. The area where the superior premaxillary nerve (nPMs) extends in a bony canal or groove lateral to the external naris is shown in an orange square. Bony foramina connected to the posterior premaxillary canal (cPMp) are indicated with blue triangles. In addition, bony foramina connected to the dorsal and ventral parts of the anterior premaxillary canal (cPMa) are shown in yellow and orange triangles, respectively. cDN, dorsal canal in mesethmoid; fPMa, premaxillary foramen connected to cPMa; nALMx, maxillary alveolar nerve; nNm, medial nasal nerve; nPMi, inferior premaxillary nerve.

### 3.2 The Maxillary Nerve Pathways in Aves

1. **Orbital region**
  In the orbita, the maxillary nerve generally extends beneath the eyeball as the infraorbital nerve (*ramus infraorbitalis*) toward the posterior end of the beak (Watanabe and Yasuda 1970). It branches off some cutaneous nerves for the lower eyelid and is identified in *P. carbo*, *M. undulatus*, and *T. eunomus* (Figures 3, 4B, S2C, S3B, and S4A). In *M. undulatus*, one cutaneous branch passes through a short bony canal within a suborbital arch (Figure S12A–B).
  The main infraorbital nerve then extends anteriorly toward the anterior end of the orbita and follows several species‐specific nerve pathways. In *A. platyrhynchos*, the nerve extends forward along a large depression on the posterodorsal surface of the palatine while giving off a thick nasopalatine nerve (*ramus nasopalatinus*) anteroventrally (Figure 9A–B). In *M. undulatus*, the infraorbital nerve extends anteroventrally between the infraorbital and preorbital diverticulae (Faux and Logsdon 2022) of the infraorbital sinus, continues along the ventrolateral surface of the ethmomandibular muscle (Carril et al. 2015), and reaches the ventral wall of the respiratory turbinate. In contrast, the nerve courses along the ventral wall of the ectethmoid in *C. livia* (Figure S10A,C–F), between the ectethmoid and maxilla in *F. tinnunculus*, and through an ectethmoid canal in *Turdus* (Figure S4A–E). In *S. camelus*, this nerve extends between the antorbital sinus and maxillopalatine process (Figure S9), whereas Webb (1957) described the nerve as passing ventral to the maxillopalatine process.
2. **Palatal region**
  The nasopalatine nerve generally extends ventrally or medially toward the posterior palatal surface of the beak (e.g., Bubien‐Waluzewska, 1981; McKeegan 2004). In *A. platyrhynchos*, the nerve originates in the palatine depression (Figures 8A–B and 9A–B) and passes through a large palatine foramen (Figure 9B). This nerve divides into several branches that extend anterolaterally along narrow grooves on the palatal surface of the maxilla (Figures 8A and 9C–D). In *S. camelus*, this nerve emerges near the maxillopalatine process and extends along the midline of the ventral wall of the nasal cavity (Figure S9).
3. **Maxillary region**

Within the maxilla, the main maxillary nerve generally enters the maxillary canal at the posterior end of the beak, as the maxillary alveolar nerve (*ramus alveolares maxillares* [e.g., Berkhoudt 1980; Abdel‐Kader and Fathy 2002]). This nerve gives off the lateral branch as the external maxillary nerve (*ramus maxillaris externi*), innervating epithelial tissues on the posterolateral side of the beak (Berkhoudt 1980). In *A. platyrhynchos*, the maxillary alveolar nerve enters the maxillary cavity posteriorly, divided into multiple branches (Figures 9A–B and S8C). This nerve gives rise to external maxillary nerves through lateral foramina (Figures 9A–B and S8C) as described in Berkhoudt (1980) and continues anteriorly along the ventral grooves of the maxilla and premaxilla (Figures 8A,C and 9C–F). Some ventral branches contact the nasopalatine nerve in the posterodorsal region of the beak (Figures 8A and 9C). In *M. undulatus*, the maxillary alveolar nerve is enclosed by the premaxilla dorsomedial to the jugal–premaxilla articulation (Figures 6A–B and 10A–B) and gives off external maxillary nerves toward posterolateral foramina (Figures 6C and 10). In *S. camelus*, the maxillary alveolar nerve extends through the maxillary sinus to anterior canals and foramina (Figures 3A and S9).

In *Po. cristatus*, the maxillary alveolar nerve is traced into the middle of the maxillary canal via the maxillopalatine process (Figure S11A,C–D). Although the more anterior course within the canal could not be confirmed, the maxillary canal divides into multiple canals that connect to foramina on the lateral surface of the maxilla (Figure S11C–F). Similarly, in *T. eunomus*, the course of the maxillary alveolar nerve is confirmed only within the posterior part of the maxillopalatine canal (Figure S4). In *C. livia* and *F. tinnunculus*, the anterior course of the maxillary alveolar nerve is not observed; however, posterior maxillary foramina and branching maxillary canals suggest that the nerve likely continues through an intraskeletal pathway within the maxilla (Figures 3 and S10).

### 3.3 The Mandibular Nerve Pathways in Aves

1. **Posterior orbital region**
  The mandibular nerve generally extends anteroventrally in the posterior side of the orbita surrounded by jaw muscles (Holliday and Witmer 2007). Before entering the mandibular canal, the mandibular nerve gives off several motor and sensory branches, some of which were observed in a previous study. The pterygoid nerve (*ramus pterygoideus*), which innervates the pterygoid and pseudotemporal muscles (e.g., Barnikol 1954; Dubbeldam 1993), is confirmed in all examined species except *P. carbo* (Figures 4 and S1–S3). The nerve innervating *musculus protractor quadratus* is confirmed in *M. undulatus* (Figure 4B), and the nerve innervating *musculus adductor mandibulae externum* is identified in *A. platyrhynchos* and *F. tinnunculus* (Figures 4A and S3A). The nerve innervating *anguli oris* is also observed in *A. platyrhynchos* and *M. undulatus* (Figures 4 and 12).
2. **Around the primordial foramen**
  Before entering the mandibular canal, the mandibular nerve gives off the external mandibular nerve (*ramus mandibularis externus*), a cutaneous sensory branch that generally extends through a neurovascular canal in the surangular (Watanabe and Yasuda 1970; Dubbeldam 1993). This nerve is confirmed in *S. camelus*, *P. cristatus*, *A. platyrhynchos*, and *C. livia* (Figures 4A, S1, and S2A). In *Anas*, its course corresponded to a large depression on the lateral surface of the surangular and dentary (Figures 12C and 13).
  The main mandibular nerve also gives off the intermandibular nerve (*ramus intermandibularis*) ventromedially, innervating the intermandibular muscles and epidermis (Watanabe and Yasuda 1970). This nerve is observed in *S. camelus*, *P. cristatus*, and *A. platyrhynchos* (Figures 4A and S1). In *A. platyrhynchos*, this nerve passes through a bony fossa between the surangular and splenial bones (Figure 12A–B), whereas in *P. cristatus*, it passes between a bony fossa between the prearticular and splenial bones (Figure S13A–B).
3. **Within the mandibular canal**

The main mandibular nerve generally enters the mandibular canal through the primordial foramen, as the mandibular alveolar nerve (*ramus alveolares mandibulares*) toward the bill tip region (e.g., Dubbeldam 1993). In *A. platyrhynchos*, the mandibular canal continues anteriorly as numerous small canals extend toward the lateral and medial surfaces of the dentary (Figures 3, 12, and 13I). Within these canals, the mandibular alveolar nerve gives off several branches toward the epidermis of the posterolateral side of the beak (Figures 12 and 13I). In contrast, in *C. livia*, the mandibular alveolar nerve remains enclosed within the dentary before the bill tip region and becomes exposed on the bony surface through an elongated fenestra (Figure 11A–B). In *S. camelus*, the mandibular alveolar nerve enters the mandibular canal only posterior to the mandibular symphysis and the posterior courses corresponding to the shallow depression on the medial surfaces of the splenial and dentary (Figure S9C–E), as previously described (Webb 1957).

**Figure 11:**
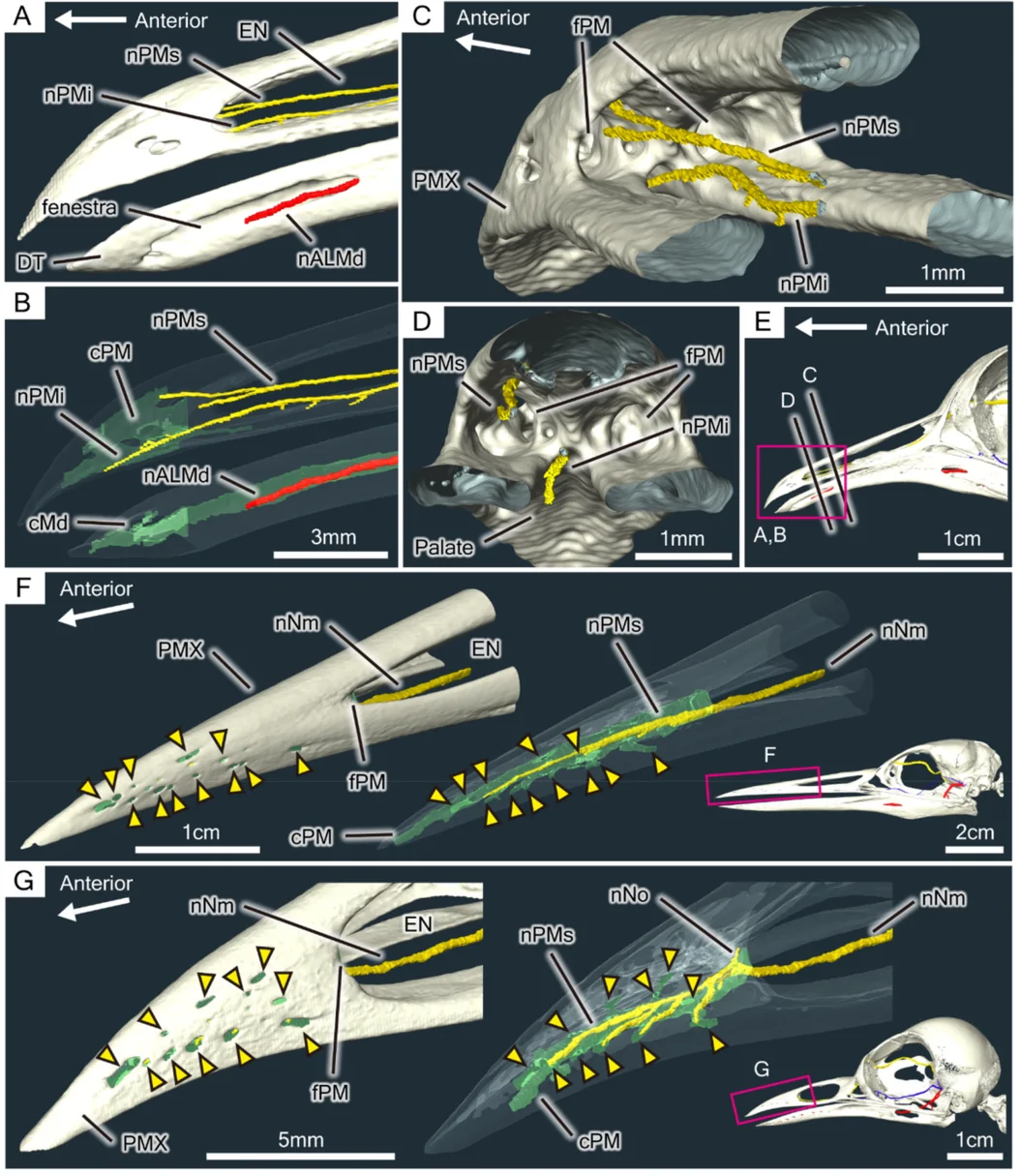
The ophthalmic nerve pathways in the snout tip of *Columba livia* (A–D), *Po. cristatus* (F) and *Turdus eunomus* (G). *Note*. The nerve pathways in *Columba* are shown in left lateral (A, B), left posterolateral (C), and posterior view of the transverse section (D) at the level indicated in (E), respectively. In (F) and (G), the nerve pathways of *Podiceps* and *Turdus* in the areas indicated in the lower right figure are shown with solid and transparent skulls on left and middle, respectively, with the bony foramina connected to the premaxillary canals (cPM) being indicated by yellow triangles. cMd, mandibular canal; DT, dentary; EN, external naris; fPM, premaxillary foramen; nALMd, mandibular alveolar nerve; nNm, medial nasal nerve; nNo, oral nasal branch; nPMi, inferior premaxillary nerve; nPMs, superior premaxillary nerve; PMX, premaxilla.

Within the mandibular canal, the mandibular alveolar nerve generally gives off the sublingual nerve (*ramus sublingualis*), which innervates the mucous membrane or salivary glands (Watanabe and Yasuda 1970), and is observed in *A. platyrhynchos* and *C. livia* (Figures 4A and S2A). In *Anas*, a thick sublingual nerve emerges within the mandibular canal (Figure 13B–D) and extends anteriorly medial to the mandibular alveolar nerve. The sublingual nerve gives off several medial branches toward the middle part of the mandible (Figure 13B–G), whereas the mandibular alveolar nerve itself has no additional branches (Figures 12F and 13I). In *C. livia*, the sublingual nerve emerges posterior to the primordial foramen and extends medially toward the lingual mucosa (Figure S14C–D), as described previously (Cords 1904; Bubien‐Waluszewska 1981).

**Figure 12:**
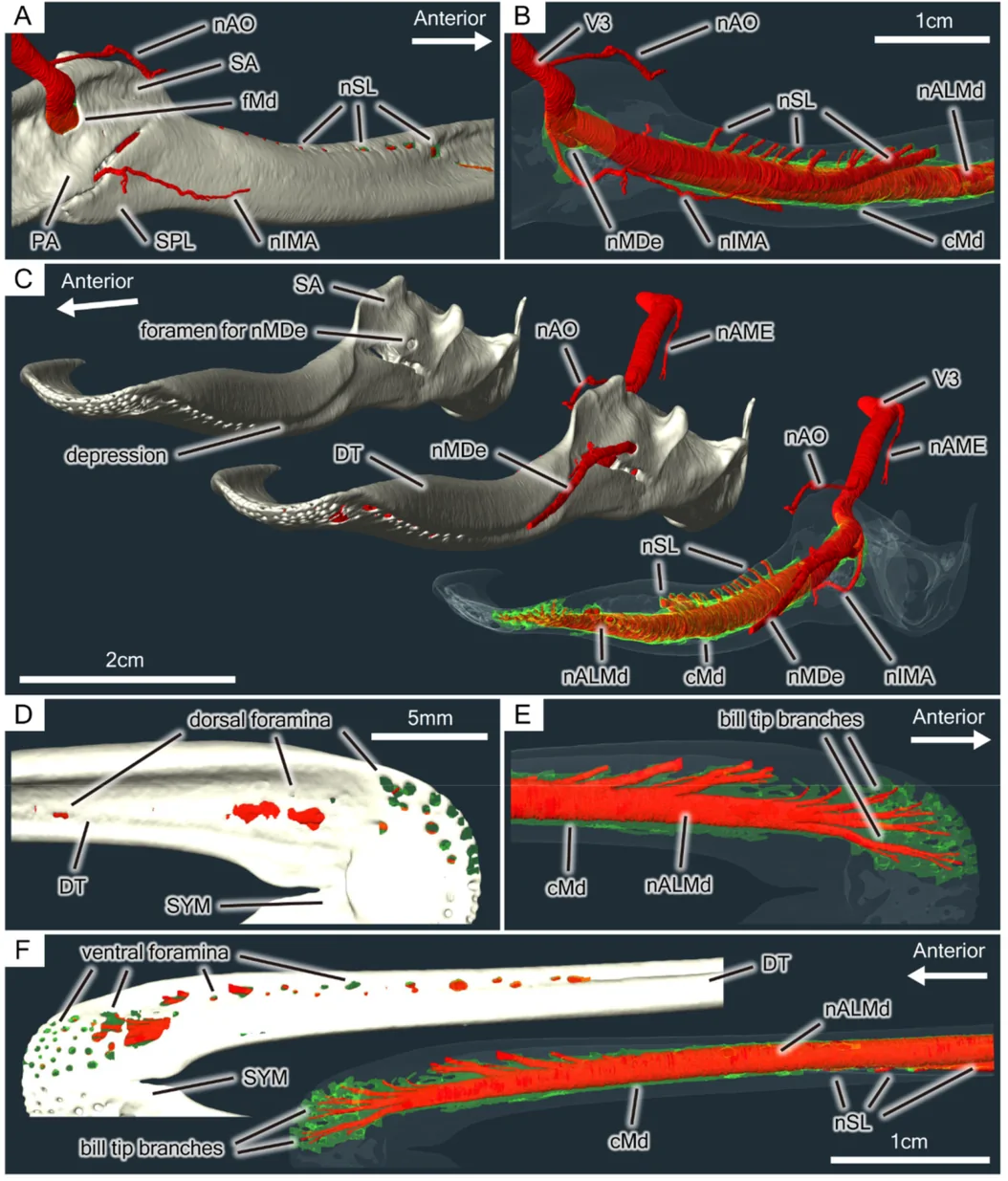
The mandibular nerve pathway in the mandible of *Anas platyrhynchos.* (A, B) Medial view of the right ramus with a solid and transparent skull, respectively. (C) Left anterolateral view of the left ramus, skeleton (top), and solid and transparent skeleton with the nerve (middle and bottom). (D, E) Dorsal view of the mandibular with and without the skeleton being transparent, respectively. (F) Dorsal view of the mandible with a solid (left) and a transparent skull (right). cMd, mandibular canal; DT, dentary; fMd, mandibular foramen; nALMd, mandibular alveolar nerve; nAME, nerve for external adductor muscle of the mandible; nAO, nerve for *anguli oris*; nIMA, intermandibular nerve; nMDe, external mandibular nerve; nSL, sublingual nerve; SA, surangular; SPL, splenial; SYM, mandibular symphysis; V3, mandibular nerve.

**Figure 13:**
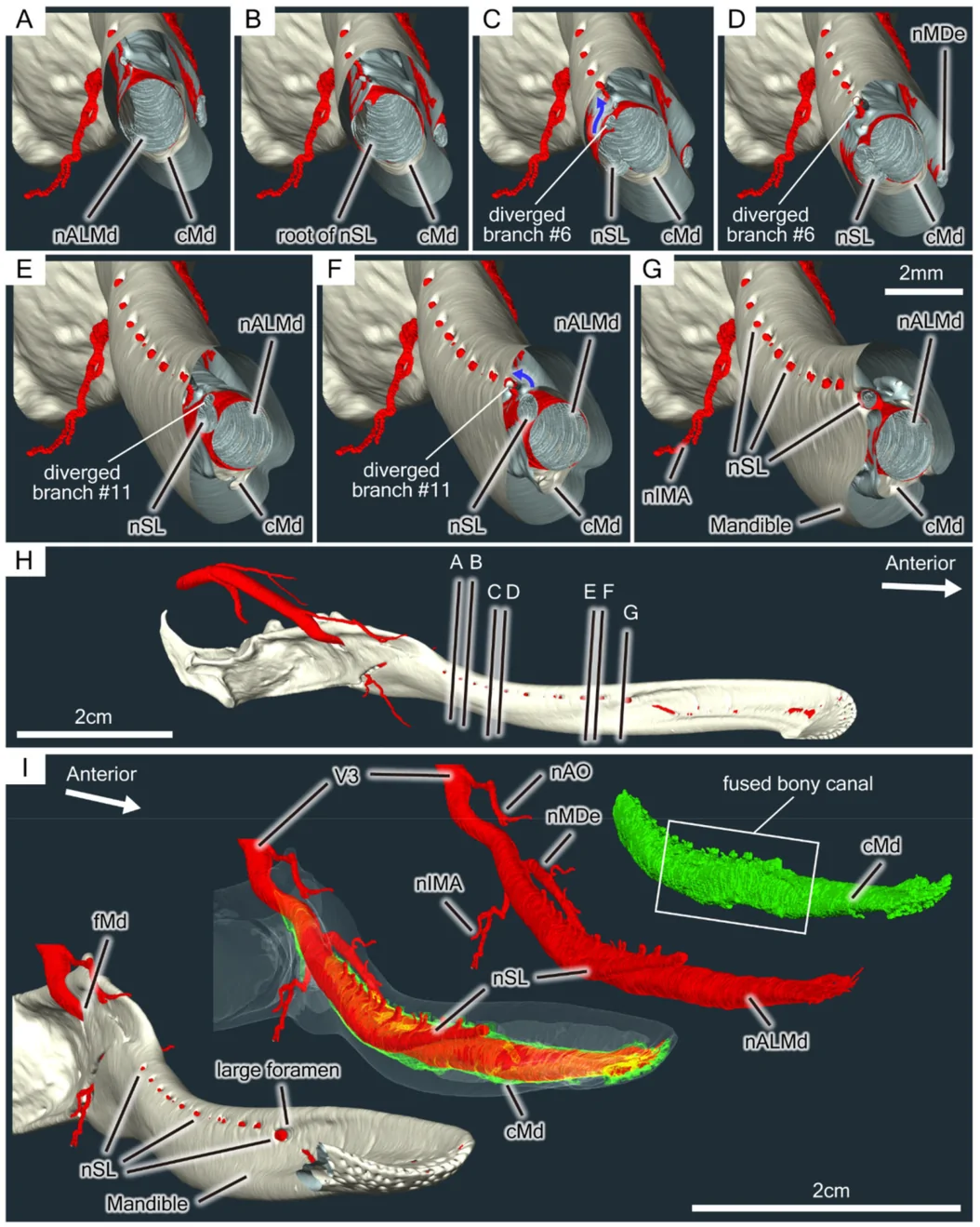
The mandibular nerve pathway of *Anas platyrhynchos.* (A–G), Right anterolateral view of transverse sections of the left ramus at the levels indicated in (H). In (C, F), the nerve pathways of diverged branches of the sublingual nerve (nSL) from the mandibular canal (cMd) to the medial foramina of the mandible are indicated with blue lines with an arrowhead. (H) Medial view of the left ramus. (I) Anteromedial view of the left ramus, showing, from left to right, the nerves with a solid and transparent skull, nerves only, and mandibular canal that houses the nerves. The part where the main sublingual nerve extends with the mandibular alveolar nerve (nALMd) within the mandibular canal is indicated by a white square in the right‐most figure. fMd, mandibular foramen; nAO, nerve for *anguli oris*; nIMA, intermandibular nerve; nMDe, external mandibular nerve; nSL, sublingual nerve.

### 3.4 Trigeminal Nerve Pathways in Crocodylia

To infer the ancestral condition of the trigeminal nerve pathway in Aves, one specimen for each of two crocodylian species, *C. crocodilus* and *C. siamensis*, was observed as an outgroup. The trigeminal nerve pathways are highly conserved between the two species (Figures 14 and S16–S17) and are generally consistent with previous descriptions of crocodylians (e.g., Bellairs and Shute 1953; Gignac and Kley 2014; Lessner and Holliday 2020; Lessner et al. 2019, 2022). Nevertheless, the present study revealed several previously undescribed traits of intraskeletal nerve pathways.

**Figure 14:**
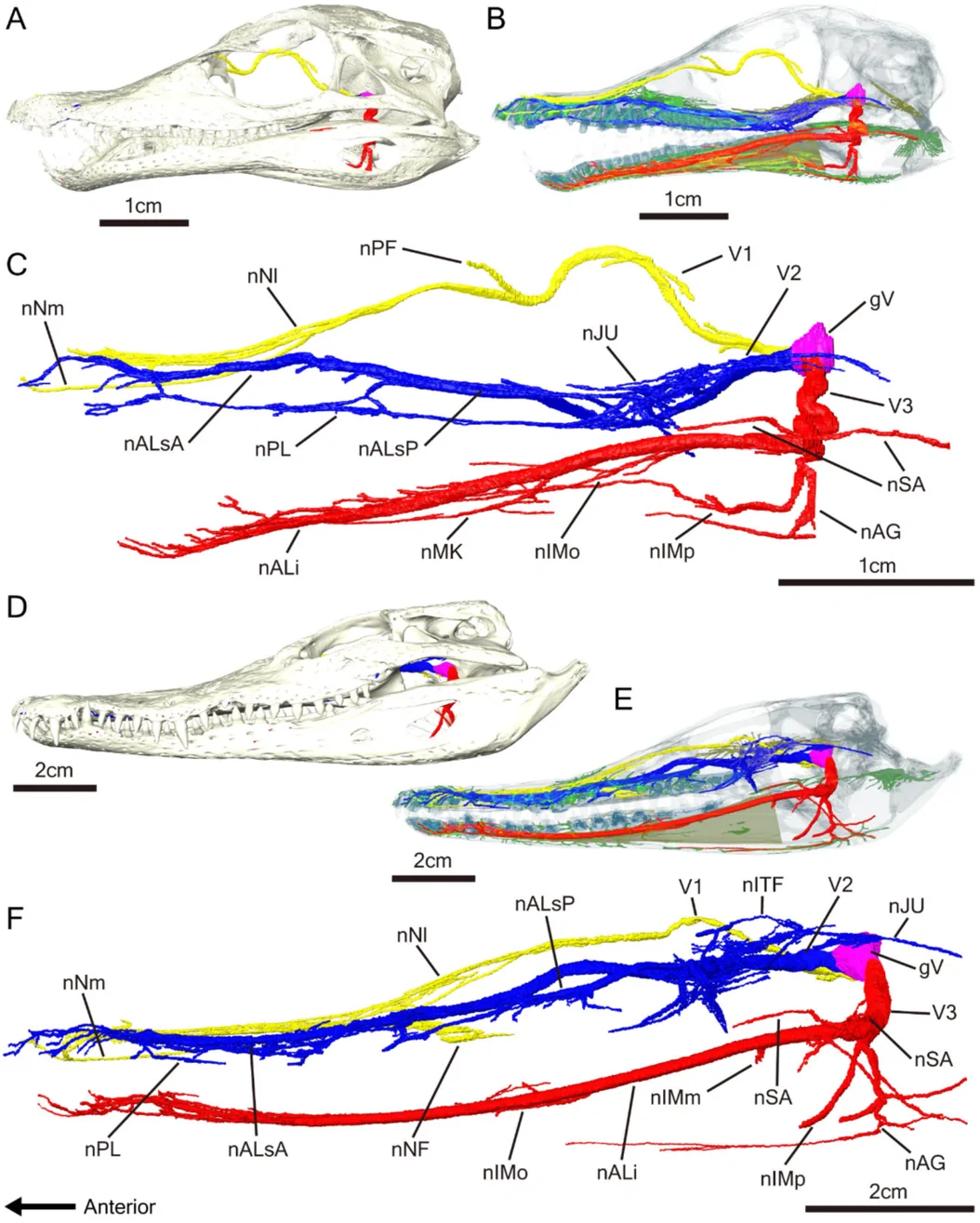
3D trigeminal nerve pathway in the skull of *Caiman crocodilus* (A–C) and *Crocodylus siamensis* (D–F) in left lateral view. (A, D) External views of skulls. (B, E) Nerve pathways in transparent skulls. The neurovascular canals and large bony cavities in the skull are shown in light green. Tooth sockets and large mandibular cavities are also shown in light blue and light yellow, respectively. (C, F) Nerve pathways. The trigeminal ganglion (gV) is in pink, the ophthalmic nerve (V1) is in yellow, the maxillary nerve (V2) is in blue, and the mandibular nerve (V3) is in red. nAG, angular branch; nALi, inferior alveolar nerve; nALsA, anterior division of the superior alveolar nerve; nALsP, posterior division of the superior alveolar nerve; nIMm, branch to the intramandibular muscle; nIMo, oral intermandibular branch; nIMp, caudal intermandibular branch; nITF, branch to infratemporal fenestra; nJU, jugal branch; nMK, Meckelian branch; nNF, branch to the nasal fontanelle; nNl, lateral nasal nerve; nNm, medial nasal nerve; nPF, branch to the posterior part of prefrontal; nPL, palatal branch; nSA, surangular branch.

### 3.5 The Ophthalmic Nerve Pathway in Crocodylia

The general branching pattern is consistent with previous reports. Anterior to the prefrontal pillar, the main ophthalmic nerve divides into the medial nasal nerve (*ramus medialis nasi*) and the lateral nasal nerve (*ramus lateralis nasi*, Figures 14 and S16–S17). The medial nasal nerve extends anteromedially along the cartilaginous nasal septum. Whereas the lateral nasal nerve courses anterolaterally along the dorsomedial wall of the maxilla, leaving shallow grooves as osteological correlates near the maxillary caniniform tooth (Figure 15C–D). At the lateral bony wall of the external naris, one branch of the lateral nasal nerve enters premaxillary canals and joins the maxillary (superior alveolar) nerve (Figure 15A–C,E). In addition, the lateral and medial nasal nerves unite to form the intrapremaxillary nerve, which enters another premaxillary canal toward the snout tip (Figure 15E–F).

**Figure 15:**
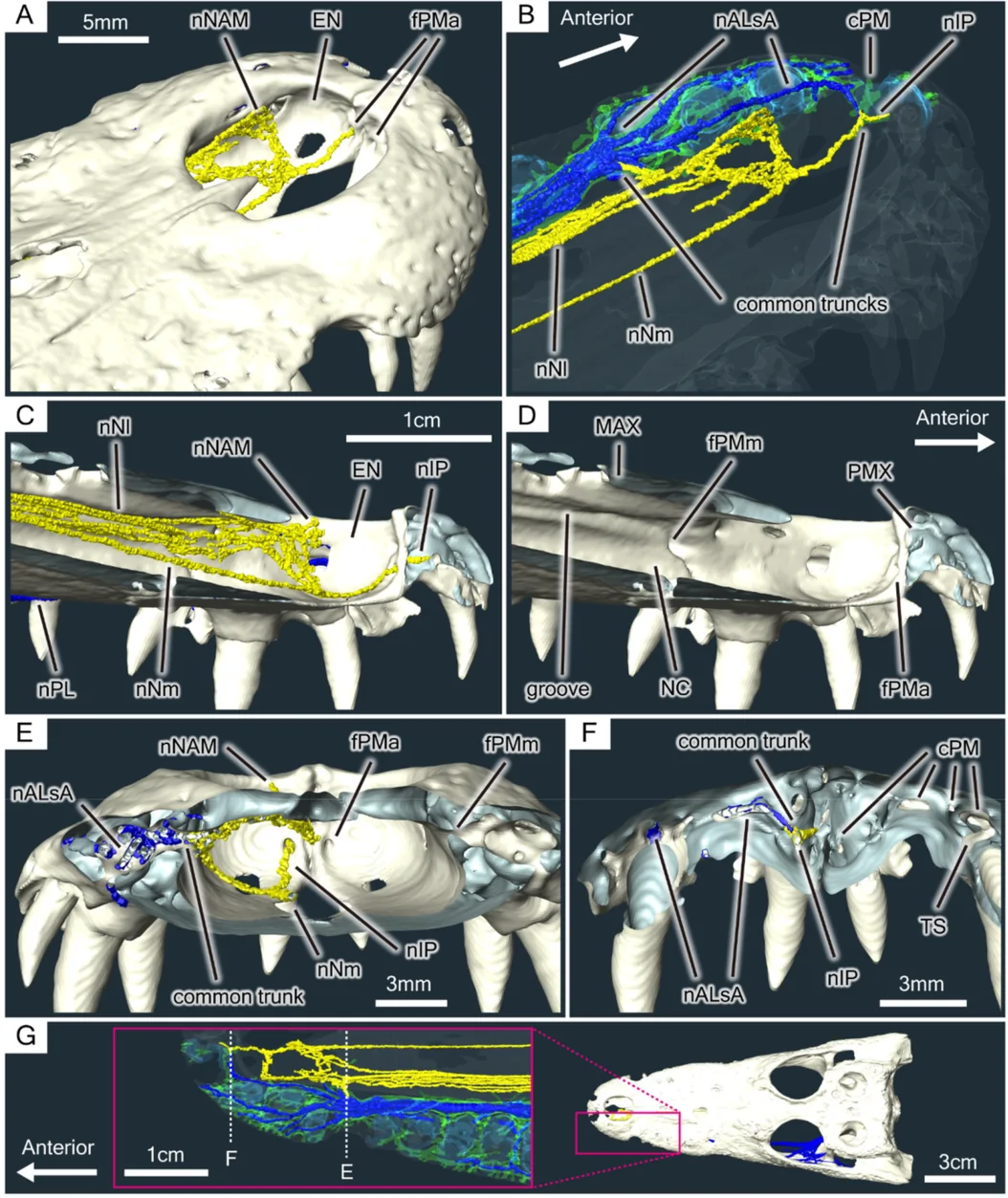
Ophthalmic and maxillary nerve pathways at the anterior tip of the snout of *Crocodylus siamensis.* (A–F) Close‐up views of the areas or at the levels indicated in (G). (A, B) Right posterolateral view with and without the skull being transparent, respectively. (C, D) Left medial view with and without the nerves, respectively. (E, F) Posterior view of the transverse sections at the levels indicated in (G). (G) Dorsal view of the skull (right), and close‐up nerve pathways (left). cPM, premaxillary canal; EN, external naris; fPMa, premaxillary foramen anterior to the external naris; fPMm, medial premaxillary foramen; MAX, maxilla; nALsA, anterior division of superior alveolar nerve; NC, nasal cavity; nIP, intrapremaxillary nerve; nNAM, branch to the narial musculature; nNl, lateral nasal nerve; nNm, medial nasal nerve; nPL, palatal branch; PMX, premaxilla; TS, tooth socket.

### 3.6 The Maxillary Nerve Pathway in Crocodylia

The general branching pattern of the maxillary nerve corresponded to previous studies (e.g., Lessner et al. 2019, 2022; Lessner and Holliday 2022). In the orbita, the main maxillary nerve divides into four major branches: the palatal, jugal, posterior superior alveolar, and anterior superior alveolar nerves (Figures 14, 16, and S16–S20). The palatal branch of *C. crocodilus* takes a course to the lateral maxillary palate, connecting with the nerve emerging from the maxillary alveolar canals. The jugal branches have longitudinal intraskeletal pathways in the jugal bone; however, these canals do not continue into the adjacent maxilla or ectopterygoid bones (Figures S18–S19). The posterior superior alveolar nerve enters the maxillary canal from the posteromedial side of the maxilla, whereas the anterior superior nerve enters the maxillary canal anterior to the orbita (Figures 16 and S16–S18). These two nerves unite just posterior to the maxillary caniniform tooth (Figure 16). The anterior part of the snout, branches of the superior alveolar nerve, continues to enter the premaxillary canal (Figures 16 and S20). At the snout tip in *C. siamensis*, the superior alveolar nerve unites with the intrapremaxillary nerve (Figures 15F and 16D).

**Figure 16:**
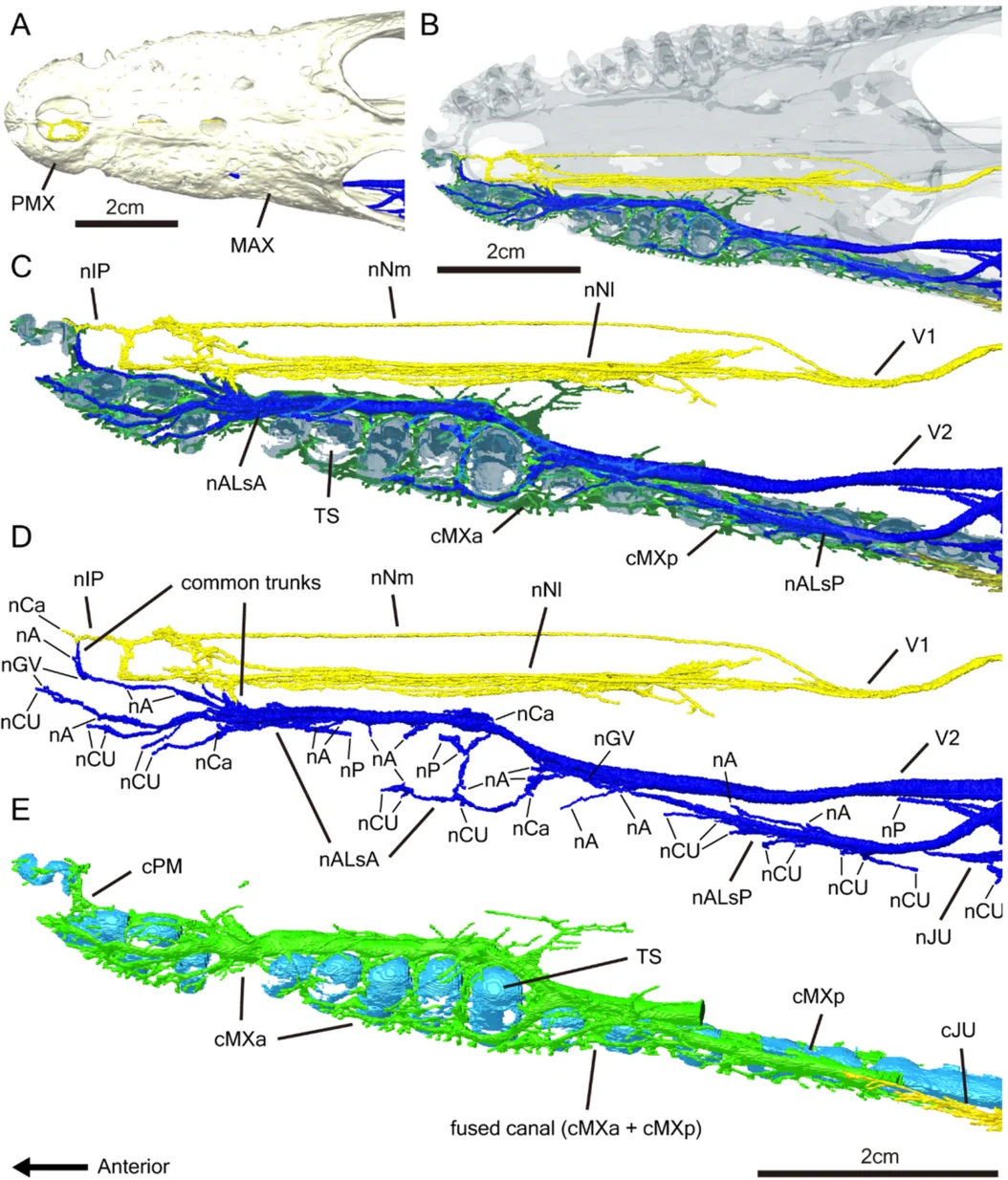
Ophthalmic and maxillary nerve pathway in the snout region of *Crocodylus siamensis.* (A, B) Nerves with a solid and transparent skull. (C) Detailed branching patterns of the nerves are shown with transparent tooth sockets (TS; light blue), maxillary and premaxillary canals (light green), and jugal canal (cJU; light yellow). (D) Same as (C), but only nerves are shown. (E) Bony structures shown in (C). cMXa, anterior part of the maxillary canal; cMXp, posterior part of the maxillary canal; cPM, premaxillary canal; MAX, maxilla; nA, branch to the tooth alveoli; nALsA, anterior division of superior alveolar nerve; nALsP, posterior division of the superior alveolar nerve; nCa, branch with nerve ending within the maxillary canal; nCU; cutaneous branch; nGV, gingival branch; nIP, intrapremaxillary nerve; nJU, jugal branch; nNl, lateral nasal nerve; nNm, medial nasal nerve; nP, branch to palatal surface; PMX, premaxilla; V1, ophthalmic nerve; V2, maxillary nerve.

### 3.7 The Mandibular Nerve Pathway in Crocodylia

The general branching pattern of the mandibular nerve corresponds to previous descriptions (Schumacher 1973; George and Holliday 2013; Lessner et al. 2019, 2022). Anterior to the maxillomandibular foramen, the mandibular nerve extends toward the primordial fossa, where it is divided into the surangular, angular, posterior intermandibular, medial intermandibular, oral intermandibular, inferior alveolar, and Meckelian branches (Figures 14, S16–S17, and S21). The surangular, angular, and inferior alveolar branches generally follow previous descriptions. However, several species‐specific variations are observed. In the surangular, the anterior and posterior bony canals are continuous in *C. crocodilus* but separated in *C. siamensis* (Figures 14 and S21). The posterior intermandibular branch passes through the caudal intermandibular foramen (Ristevski 2022), but in *C. crocodilus*, it extends anteriorly along the splenial and unites with the oral intermandibular branch in the middle region of the mandible (Figures S22–S23). In contrast, the medial intermandibular branch passes through the middle intermandibular foramen only in *C. siamensis*. This condition was previously described in *A. mississippiensis* and *Crocodylus porosus* (Schumacher 1973) but not observed in *Caiman* in this study.

## 4 Discussion

### 4.1 Comparative Anatomy of the Trigeminal Nerve in Aves

This study provides the first comparative set of three‐dimensional reconstruction of the trigeminal nerve pathways extending from the trigeminal ganglion to the bill tip region in a broad range of avian taxa. The present observations demonstrate a substantial systematic variation in the branching patterns and intraskeletal courses of peripheral branches and provide a basis for interpreting their evolutionary and/or functional changes and their osteological correlates in the avian lineage.

### 4.2 Trigeminal Nerve Pathways in the Orbita

The arrangement of the ophthalmic and maxillary nerves in the orbita is highly conserved among the examined birds. The ophthalmic nerve consistently extends dorsomedially to the eyeball, whereas the infraorbital nerve follows the ventrolateral side of the orbita, consistent with previous descriptions (Watanabe and Yasuda 1970; Abdel‐Kader and Fathy 2002; Amendano et al. 2021). These observations indicate that the major orbital branches of the trigeminal nerve have remained evolutionarily conserved in Aves.

Although taxonomic variations are observed in the nerve course in the anterior orbital region, some patterns are shared among the examined birds. In *C. livia*, *M. undulatus*, and *T. eunomus*, the medial nasal nerve passes through the *foramen orbitonasale mediale* on the mesethmoidal wall separating the orbita from the nasal cavity. This foramen is also present on the transverse ethmoidal wall in a wide range of birds (Livezey and Zusi 2006), suggesting that the course of the medial nasal nerve is associated with a consistent osteological correlate.

A similar relationship between nerve pathways and osteological structures is observed for the external cutaneous nerve. In *Gallus* (Watanabe and Yasuda 1970), *Gallinula* (Abdel‐Kader 2000), and *Nyctanassa* (Amendano et al. 2021), the external cutaneous nerve beneath the eyeball is known as the epithelial sensory branch. In *M. undulatus*, this nerve extends through a distinct bony canal within the suborbital arch. Furthermore, although the suborbital arch evolved independently in Psittaciformes and Scolopacidae (Elzanowski and Mayr 2018), both clades possess canals that likely accommodate the external cutaneous nerve (Figure S12).

These observations indicate that trigeminal pathways are generally conserved and leave reliable osteological correlates, including canals and foramina. Such relatively conserved orbital nerve pathways possibly reflect the strong morphological integration of the avian cranium. If the course of the trigeminal nerve is constrained by the spatial relationship between the beak and braincase, its conservation may reflect the integrated evolution of these two cranial regions rather than their independent modification. This pattern is consistent with previous studies showing that the evolution of the beak and braincase has been tightly integrated through craniofacial architecture and allometric constraints (Bright et al. 2016; Marugán‐Lobón et al. 2022).

### 4.3 Evolution of Innervation Pattern Around the External Naris

The distribution of the ophthalmic nerve around the external naris shows considerably greater variation than that in the orbita. Previous studies have reported that the oral nasal and external nasal nerves innervate the anterior and posterior margins of the external naris, respectively (*Galllus* [Watanabe and Yasuda 1970]; *Anas* [Berkhoudt 1980]). In the present study, this pattern is confirmed in *A. platyrhynchos* and *T. eunomus*, whereas *F. tinnunculus*, *M. undulatus*, and *S. camelus* show different patterns. In *Falco*, the oral–nasal nerve innervates both the anterior and posterior margins of the naris. In *M. undulatus*, the posterior narial region is innervated by the superior premaxillary nerve together with the external nasal nerve. These observations suggest that the pattern observed in galloanserans, as well as in *T. eunomus*, represents the ancestral condition for Neognathae, whereas *F. tinnunculus* and *M. undulatus* independently evolved derived innervation patterns. In *F. tinnunculus*, the expansion of the region supplied by the oral nasal nerve indicates that it becomes the predominant sensory branch around the naris. In *M. undulatus*, the region innervated by the external nasal nerve has shifts dorsally toward the area innervated by the medial nasal nerve in other birds, whereas the superior premaxillary nerve expands its distribution to innervate the posteroventral margin of the naris. These modified patterns may be associated with increased ossification of the nasal region (Tada and Tsuihiji 2021), evolution of advanced cranial kinesis (Tokita 2003; Pecsics et al. 2020), modifications related to the bill‐tip organ (Demery et al. 2011), aerodynamic specialization of narial airflow (Kesel et al. 2019), and/or dietary specialization (Bright et al. 2016; Navalón et al. 2019).

### 4.4 Evolution of the Intraskeletal Maxillary Pathway

The present observations show that an intraskeletal course of the maxillary nerve is a common feature in birds, although the specific pathway varies among taxa. In *A. platyrhynchos*, *Po. cristatus*, and *T. eunomus*, the nerve extends through the maxillary canal from the palatine process of the maxilla, whereas it passes through the maxillary pneumatic recess in *Struthio* (Mayr 2020). In *M. undulatus*, the nerve passes through the premaxillary canal in the jugal‐contacting region. *C. livia* and *F. tinnunculus* probably retain an intraskeletal pathway originating from the maxillopalatine process, and *P. cristatus* possesses a pneumatic recess similar to that in *S. camelus*. Together with previous recognition of the maxillary canal in *Gallinula* (starting from the maxillopalatine process; Abdel‐Kader 2000) and *Melops* (starting from the zygomatic process; Abdel‐Kader and Fathy 2002), as well as the large intraskeletal cavity in *Nyctanassa* (Amendano et al. 2021), these observations suggest that the ancestral avian condition involved an intraskeletal maxillary pathway, with lineage‐specific modifications having been acquired at least in Psittaciformes and Pelecaniformes.

Additional comparative data are needed to reconstruct the ancestral condition of this nerve pathway more robustly. Mayr (2020) described a considerable variation in the morphology of the maxilla, including the presence or absence of canals, foramina, and pneumatic recesses, across major avian clades. The present observations add to such diversity by describing a canal anterior to the pneumatic recess in *S. camelus* and a canal associated with the ectethmoid and maxillopalatine process in *T. eunomus*. These structures likely represent osteological correlates of the maxillary alveolar nerve, although some canals may also transmit blood vessels, as described in *Meleagris* (Porter and Witmer 2016). Distinguishing the relative contributions of nerves and blood vessels to these intraskeletal pathways will require additional anatomical data on a broader range of taxa. For example, the middle course of the maxillary alveolar nerve remains poorly documented even in otherwise well‐studied *Gallus* (Watanabe and Yasuda 1970; Bubien‐Waluszewska 1981).

The terminal branches of the maxillary alveolar nerve also show a conserved distribution pattern among birds. The nasopalatine nerve innervates the palatal mucosa in *A. platyrhynchos*, whereas the external maxillary nerve innervates the lateral surface of the posterior upper beak in *A. platyrhynchos* and *Po. cristatus*. These observations are consistent with previous descriptions (McKeegan 2004; Higashiyama and Kuratani 2014) and suggest that the terminal branches of the maxillary alveolar nerve generally reach the palate and lateral maxilla through intraskeletal canals in birds.

### 4.5 Evolution of Mandibular Nerve Pathway

Although the resolution of the present diceCT data does not permit reconstruction of the fine motor branches, the major mandibular nerve pathway is consistently identified across the examined taxa. The branching pattern of the external mandibular and intermandibular nerves is remarkably conserved among *S. camelus*, *P. cristatus*, and *A. platyrhynchos* and is consistent with the previous descriptions on *Anas* (Dubbeldam 1993) and *Gallus* (Watanabe and Yasuda 1970). This pattern likely represents the ancestral condition of the crown clade Aves.

In contrast, the bony features associated with the posterior course of the intermandibular nerve vary between *S. camelus*, *P. cristatus*, and *A. platyrhynchos*. In addition, the intraskeletal course of the sublingual nerve also differs between *A. platyrhunchos* and *C. livia*. These differences may possibly reflect evolutionary changes in jaw musculature architecture between Palaeognathae and Neognathae (Holliday 2006) or specialization associated with the feeding function unique in Anseriformes (Berkhoudt et al. 1981; Crome 1985).

The mandibular canal also shows considerable morphological diversity. *Struthio camelus* lacks a completely enclosed canal in the middle part of the mandible, as may be expected from unusually strong development of the Meckelian cartilage in this bird (Crole and Soley 2016; Lessner et al. 2023a). In *C. livia*, the canal opens laterally near the bill tip region, resembling the condition reported in pan‐alcid charadriiforms such as *Mancalla* (Smith 2011) and *Alle* (Lessner et al. 2023a). These openings may have resulted from the fusion of multiple foramina on the thin dentary cortex (Bouabdellah et al. 2022; Lessner et al. 2023a).

Previous studies have reported that the morphology of the mandibular canal varies near the bill tip region in birds (e.g., Crole and Soley 2016; Lessner et al. 2023a). Based on the results of those studies and the present observations, the following five major canal arrangements can be recognized: (1) complex, closely interconnected canals (e.g., *Dromaius*, Crole and Soley 2016; *Anas*, Lessner et al. 2023a and this study); (2) multiple canals terminating on the anterolateral margin of the mandible (Psittaciformes, Lessner et al. 2023b and this study); (3) multiple canals terminating dorsally (*Pavo*; this study); (4) a single canal terminating dorsally (e.g., *Fulica*, *Alle*, and *Pandion*; Lessner et al. 2023a; *Struthio*, *Columba*, *Phalacrocorax*, *Podiceps*, and *Turdus*, this study), and (5) a single canal terminating anteriorly (*Falco*, this study). These differences likely reflect those of feeding ecology and bill morphology, including the presence or absence of bill‐tip sensory organs (e.g., Gottschaldt and Lausmann 1974; Schneider et al. 2016; Du Toit et al. 2020; Lessner et al. 2023b).

In summary, these observations suggest that evolutionarily conservative posterior pathways of the trigeminal nerve in both upper and lower jaws are in stark contrast to those of the anterior branches innervating the beak, which are highly variable, putatively reflecting ecological and functional specialization of the snout region in birds.

### 4.6 Reconstruction of the Trigeminal Nerve Pathway in Fossil Archosaurs

Numerous studies have reconstructed trigeminal nerve pathways in fossil archosaurs based on intraskeletal neurovascular canals (e.g., Benoit et al. 2016; Barker et al. 2017). However, because these canals may transmit both nerve branches and blood vessels, their soft‐tissue contents must first be established in extant taxa (Porter and Witmer 2020). The comparative data presented here provide an anatomical framework for reconstructing the nerve pathways and evaluating their ancestral condition in Archosauria.

### 4.7 Ancestral Trigeminal Nerve Pathway in Upper Jaw of Archosaurs

Birds and crocodylians share a medial nasal nerve passing through the nasal cavity into the premaxilla. This observation, together with previous descriptions on Testudines (e.g., Ogushi 1911; Soliman 1964) and Gekkota and Varanidae (e.g., Bellairs 1949; Higashiyama et al. 2021), suggests that such an intrapremaxillary pathway represents an ancestral sauropsid condition. In contrast, the course of the maxillary alveolar nerve markedly differs between birds and crocodylians. Crocodylians have a continuous intraskeletal course through the maxilla and premaxilla and an additional intrajugal pathway, both of which have also been inferred in fossil pseudosuchians (*Wannia* and Thallattosuchia, Lessner and Stocker 2017; Bowman et al. 2022) and non‐avian dinosaurs (e.g., *Neovenator*, Barker et al. 2017; *Tyrannosaurus*, Bouabdellah et al. 2022; *Erlikosaurus*, Lautenschlager et al. 2014).

Fossil avialans show that this sauropsid condition was modified during evolution toward Aves. Premaxillary and maxillary canals remained separate in *Yanornis* (Bailleul et al. 2019), whereas more crown‐ward taxa, such as *Ichthyornis* and *Hesperornis*, showed a reduction of the maxillary nerve pathway within the premaxilla (Field et al. 2018). Together with the present observations, these data suggest that the condition of the crown Aves evolved near the origin of this clade itself. In the avian condition, the ophthalmic nerve alone occupies the premaxilla, whereas the course of the maxillary nerve is restricted within the maxilla. The evolutionary transition to such a derived condition may have been associated with tooth loss, enlargement of the premaxilla, and/or development of cranial kinesis and rhamphotheca (e.g., Bout and Zweers 2001; Wang and Hu 2017; Davis and Clarke 2022).

### 4.8 Ancestral Archosaurian Trigeminal Nerve Pathway in Lower Jaw

The evolutionary changes of the mandibular nerve pathway in Archosauria have been discussed previously (e.g., Kawabe and Hattori 2022; Bouabdellah et al. 2022; Lessner et al. 2023a); however, data on extant birds crucial for such discussion in fact remain limited. The present observations indicate that, although the main mandibular alveolar nerve follows a generally conserved course through the mandibular canal in both birds and crocodylians, several lineage‐specific modifications have evolved.

In the posterior mandible, crocodylians possess unique angular and surangular branches extending through separate bony canals, consistent with the dense distribution of nerve branches supplying numerous integumentary sensory organs (Leitch and Catania 2012; Di‐Poï and Milinkovitch 2013). In birds, comparable intraskeletal branches are absent except for the external mandibular nerve, with which sensory innervation of this region is retained in the avian lineage.

At the anterior end of the mandible, both birds and crocodylians show wide‐ranging branching patterns of the mandibular alveolar nerve. The distribution pattern of this nerve in birds, in which alveolar branches have been lost, is most parsimoniously interpreted as an apomorphy that followed tooth loss, as previously proposed for placental mammals (Ferreira‐Cardoso et al. 2019).

These avian features lacking in crocodylians recognized here demonstrate that further comparative studies of mandibular nerve pathways in birds are needed to better understand the evolution of the archosaurian mandible, including major evolutionary transitions such as tooth loss, diversification of beak morphology, and acquisition of the predentary bone in several lineages (e.g., Nabavizadeh and Weishampel 2016; Field et al. 2018; Bailleul et al. 2019). Comparisons among closely related extant species with different dietary features may also help clarify the relationships between nerve branching patterns, mandibular morphology, and sensory structures such as the sensory papillae at the dentary tip (Lessner et al. 2023b).

### 4.9 Reconstructing the Nerve Branching Pattern in Fossil Taxa Using New Bony Traits

Many studies have reconstructed neural pathways in fossil taxa from osteological correlates of the skull (e.g., Evans 2006; Porter and Witmer 2020). The present study identified several previously unrecognized correlates of the ophthalmic and maxillary nerves, including grooves on the nasal cavity wall and the entrance of the infraorbital nerve between the ectethmoid and jugal. These observations suggest that further detailed comparisons in extant taxa may improve the level of inference (*sensu* Witmer 1995) for reconstruction of trigeminal nerve pathways and associated non‐mineralized structures in fossil archosaurs.

### 4.10 Inference of the Snout Sensory Function in Fossil Taxa

The number of neurovascular foramina and branching pattern of neurovascular canals have frequently been used to infer the snout sensory function in fossil taxa (e.g., Du Toit et al. 2020; Lessner 2021; Kawabe and Hattori 2022). However, the present observations show that canal morphology does not necessarily reflect the pattern of trigeminal nerve branching. Multiple nerve branches may pass through a single canal (e.g., the mandibular alveolar and sublingual nerves in *A. platyrhynchos*) and emerge from or pass through a single opening (e.g., the mandibular alveolar nerve in *C. livia* and the medial and external nasal nerves in *T. eunomus*). Furthermore, the relationship between nerves and canals may vary among taxa because of differences in cranial pneumatization and/or the presence of enclosed blood vessels. These situations could lead to underestimating the complexity of trigeminal innervation patterns and, accordingly, the functional significance of these osteological characteristics in fossils needs to be interpreted together with other anatomical evidence. Network‐like nerve branching may also limit the accuracy of stream‐order analyses, which assume a simple progressive branching pattern (e.g., Lessner 2021).

Despite these limitations, neurovascular canals remain one of the most informative osteological correlates of the trigeminal nerve. Future studies combining osteological correlates with quantitative anatomical approaches, such as analysis of nerve fiber density relative to the foramen size (Holliday et al. 2013) or anatomical network analysis (e.g., Rasskin‐Gutman and Esteve‐Altava 2014; Plateau and Foth 2020), should improve the level or quality of inferences of sensory function in fossil taxa.

### 4.11 Methodological Consideration

As noted previously (Gignac and Kley 2014; Lessner and Holliday 2020), the current diceCT technique is often insufficient for reconstructing fine peripheral nerve branches. In particular, nerve pathways beneath the thick beak rhamphotheca and feathery skin are difficult to visualize because iodine preferentially accumulates in these keratinized tissues, causing beam‐hardening artifacts. Removing feathers and scraping off the rhamphotheca before staining with Lugol's solution, together with optimizing I_2_KI concentration (1% to 11.25% in vertebrate studies; Gignac and Kley 2018) and staining duration (Gignac and Kley 2014), contributes to improving visualization of nerve pathways.

Another problem with the diceCT method is the specimen deformation during iodine staining. Acidification of the solution can decalcify bone (Early et al. 2020), whereas dehydration and osmotic effects cause soft‐tissue shrinkage (Vervust et al. 2009; Tahara and Larsson 2013; Hedrick et al. 2018). These effects are particularly pronounced in birds because of their well‐developed cranial pneumatization and flexible cranial musculoskeletal system, making direct comparison between preCT and diceCT datasets difficult. Because segmentation of bones using diceCT images alone is challenging owing to the reduced contrast between bone and soft tissues, superimposing preCT and diceCT datasets is currently the most reliable approach for digitally identifying osteological correlates of soft tissues. Future improvements should focus on minimizing specimen deformation during iodine staining.

## 5 Conclusion

In this study, the detailed course of the trigeminal nerve was observed in various avian taxa using a contrast‐enhanced CT scanning method. Most avian species examined herein shared a similar nerve distribution pattern, with the ophthalmic nerve extending through the nasal cavity and premaxillary canal and the maxillary nerve extending through the maxillary canal toward the corner of the mouth. Because these characteristics are shared with crocodylians, they likely represent the ancestral branching pattern of Archosauria. Despite this overall similarity, the positional relationships between bones and the courses of the ophthalmic and maxillary nerves around the external naris varied among birds. Such variation is likely to be associated with morphological diversification of the beak correlated with foraging behavior. The data on the course of the trigeminal nerve in extant archosaurs provided in this study will serve as a solid basis for detailed identification of osteological correlates for this nerve system in fossil archosaurs and contribute to a better understanding of their paleo‐neuroanatomy and dynamic evolutionary history, including avian niche adaptation.

## Author Contributions

**Noriyuki Wakimizu:** conceptualization, methodology, software, data curation, investigation, validation, formal analysis, funding acquisition, visualization, resources, writing – original draft. **Takanobu Tsuihiji:** resources, writing – review and editing, supervision, project administration, validation, data curation, software, methodology, conceptualization.

## Supporting information


Supporting File

## Data Availability

The data that support the findings of this study are available on request from the corresponding author. The data are not publicly available due to privacy or ethical restrictions.
