# Supplementary material for: Intraskeletal Pathway of the Trigeminal Nerve in Modern Bird Beak: 3D Comparative Anatomy Using the diceCT Method
Source: J Morphol. 2026 Oct 28;287(11):e70188. doi: 10.1002/jmor.70188 (PMC13714627; doi:10.1002/jmor.70188)
Supplement: Supplementary file 1 [file JMOR-287-e70188-s001.pdf]

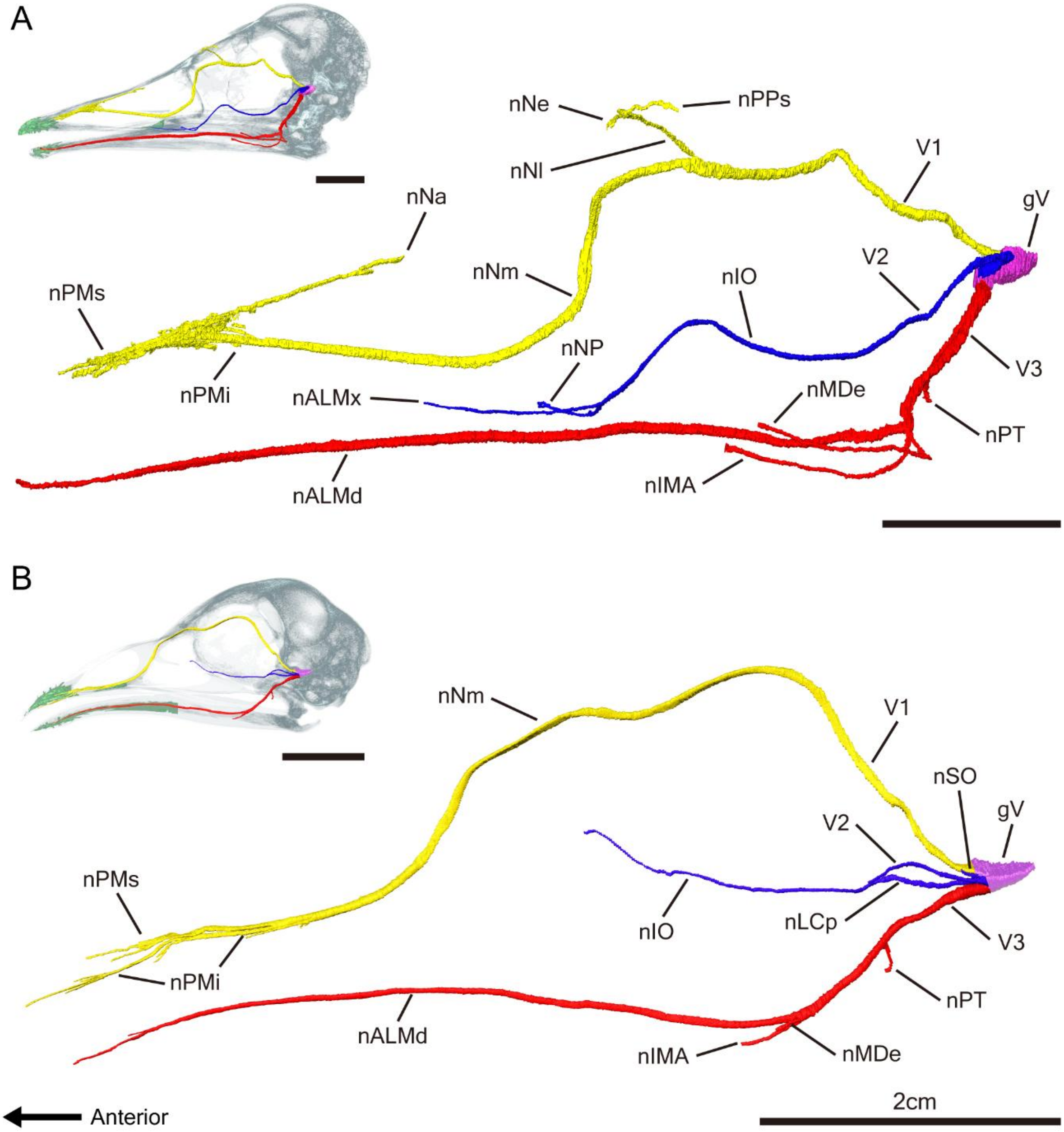

Figure S1.

Detailed view of 3D reconstruction of the trigeminal nerve in *Struthio camelus* (A) and *Pavo cristatus* (B) in left lateral view.

Note. In (A-B), the relationships of the trigeminal nerve course to the skull are shown in the left column. Abbreviations: gV, trigeminal ganglion; nALMd, intramandibular nerve; nALMx, maxillary alveolar nerve; nAME, nerve for external adductor muscle of the mandible; nIMA, intermandibular nerve; nIO, infraorbital nerve; nLCp, lacrimal palpebral nerve; nMDe, external mandibular nerve; nMXe, external maxillary nerve; nNa, nasal branch; nNe, external nasal nerve; nNl, lateral nasal nerve; nNm, medial nasal nerve; nNP, nasopalatine nerve; nPMi, inferior premaxillary nerve; nPMs, superior premaxillary nerve; nPPs, superior palpebral nerve; nPT, pterygoid nerve; nSO, supraorbital nerve; V1, ophthalmic nerve; V2, maxillary nerve; V3, mandibular nerve.

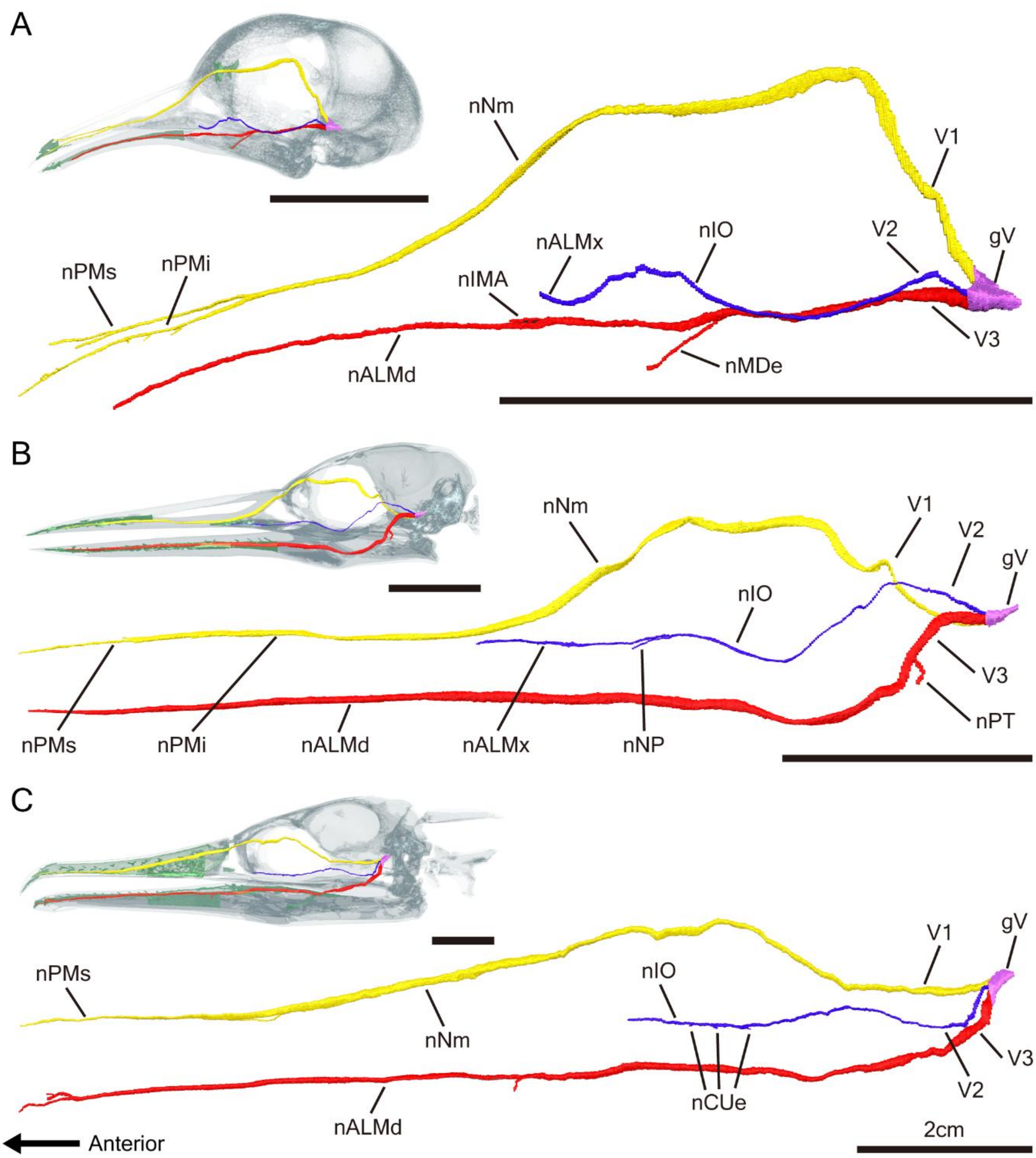

Figure S2.

Detailed view of 3D reconstruction of the trigeminal nerve in *Columba livia* (A), *Podiceps cristatus* (B), and *Phalacrocorax carbo* (C) in left lateral view.

Note. In (A-C), the relationships of the trigeminal nerve course to the skull are shown in the left column. Abbreviations: gV, trigeminal ganglion; nALMd, intramandibular nerve; nALMx, maxillary alveolar nerve; nAME, nerve for external adductor muscle of the mandible; nCUe, external cutaneous nerve; nIMA, intermandibular nerve; nIO, infraorbital nerve; nMDe, external mandibular nerve; nNe, external nasal nerve; nNi, internal nasal nerve; nNl, lateral nasal nerve; nNm, medial nasal nerve; nNo, oral nasal branch; nNP, nasopalatine nerve; nPMi, inferior premaxillary nerve; nPMs, superior premaxillary nerve; nPT, pterygoid nerve; V1, ophthalmic nerve; V2, maxillary nerve; V3, mandibular nerve. Scalebars: 2cm.

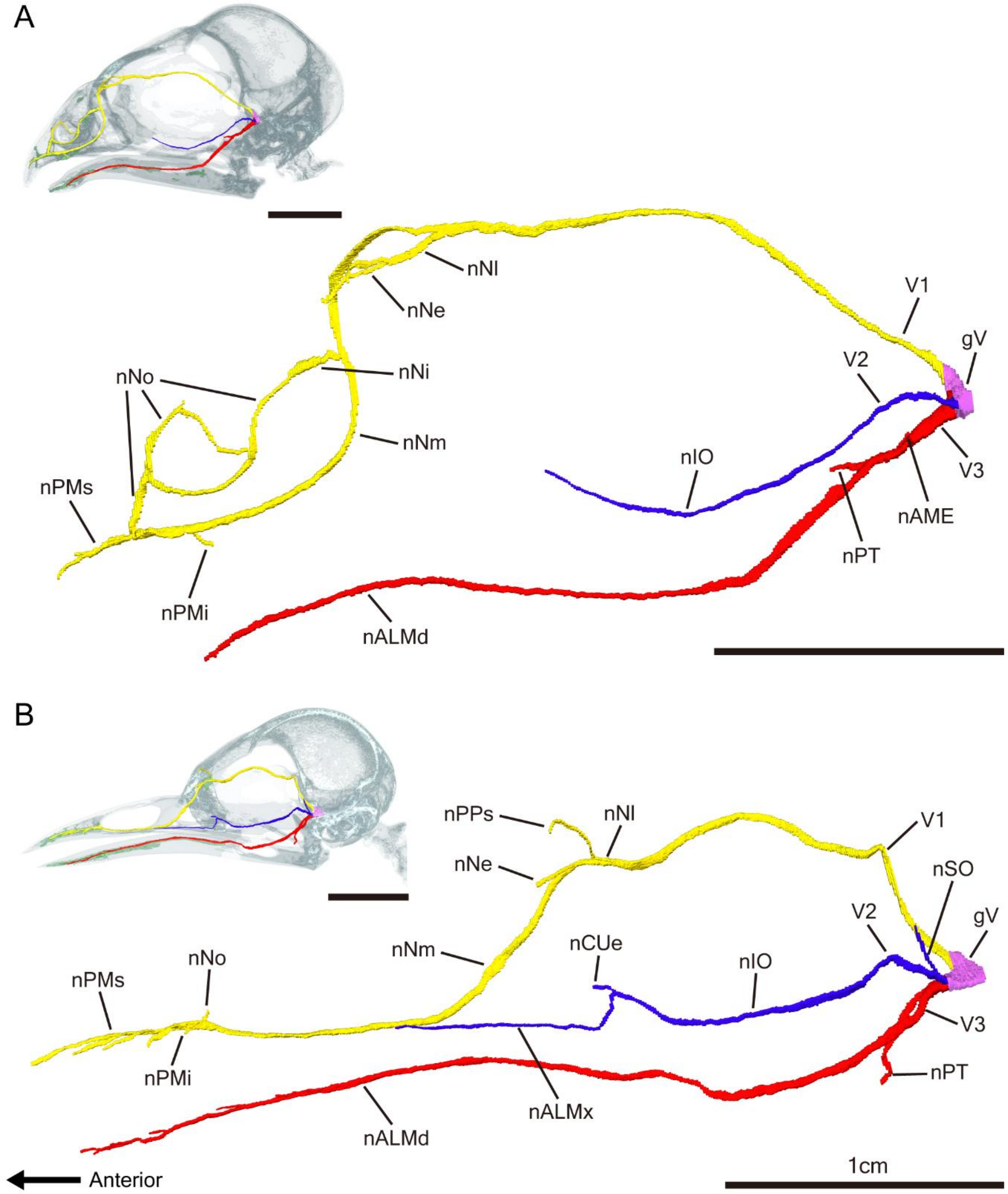

Figure S3.

Detailed view of 3D reconstruction of the trigeminal nerve of *Falco tinnunculus* (A) and *Turdus eunomus* (B) in left lateral view.

Note. In (A, B), the relationships of the trigeminal nerve course to the skull are shown in the left column. Abbreviations: gEM, ethmoidal ganglion; gV, trigeminal ganglion; nALMd, intramandibular nerve; nALMx, maxillary alveolar nerve; nCUe, external cutaneous nerve; nIO, infraorbital nerve; nNe, external nasal nerve; nNl, lateral nasal nerve; nNm, medial nasal nerve; nNo, oral nasal branch; nPMi, inferior premaxillary nerve; nPMs, superior premaxillary nerve; nPPs, superior palpebral nerve; nPT, pterygoid nerve; nSO, supraorbital nerve; V1, ophthalmic nerve; V2, maxillary nerve; V3, mandibular nerve. Scalebars: 1cm.

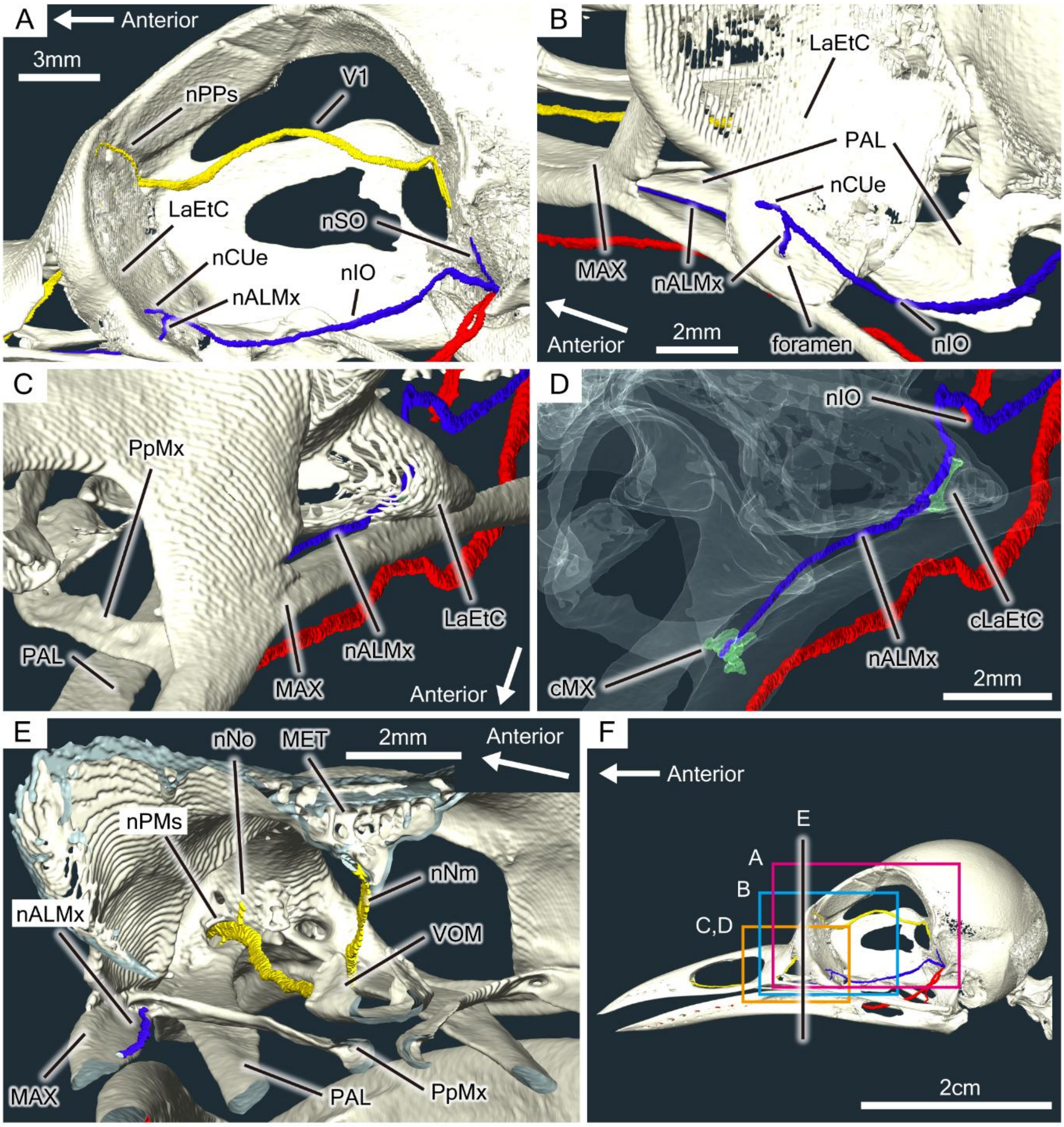

Figure S4.

The middle parts of the ophthalmic and maxillary nerves in *Turdus eunomus.*

Note. (A, B), Orbital region in left lateral and left posterolateral views, respectively. (C, D), Boundary area between the orbital and rostral regions in left anterolateral view with a solid (C) and transparent (D) skulls, respectively. (E), Transverse section at the level indicated in (F) in left posterolateral view. (F), Left lateral view of the skull indicating the viewpoints shown in (A-E). Abbreviations: cLaEtC, bony canal in lacrimal-ethmoid bone complex; cMX, maxillary canal; LaEtC, lacrimal-ethmoid bone complex, MAX, maxilla, MET, methethmoid; nALMx, maxillary alveolar nerve; nCUe, external cutaneous nerve; nIO, infraorbital nerve; nNm, medial nasal nerve; nNo, oral nasal branch; nPMs, superior premaxillary nerve; nPPs, superior palpebral nerve; nSO, supraorbital nerve; PAL, palatine; PpMx, palatal process of maxilla, V1, ophthalmic nerve, VOM, vomer.

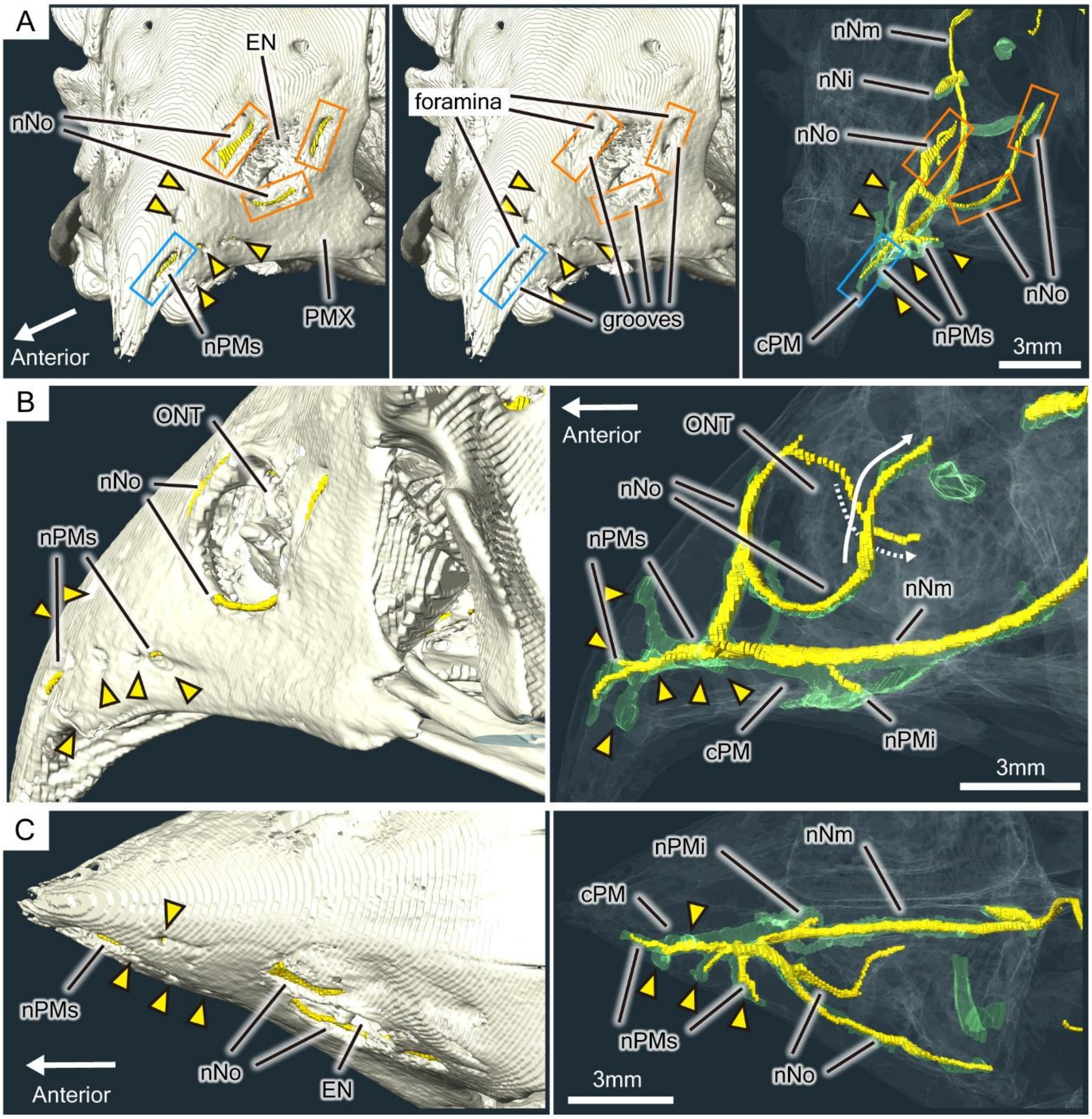

Figure S5.

Ophthalmic nerve pathway and maxillary nerve-related bony traits in *Falco tinnunculus.*

Note. (A-C), Close-up of the beak in left anterolateral (A), in lateral (B), and in dorsal (C) views. In (A-C), the intraskeletal nerve pathways are also shown in the right figures. In (A), in addition, the extra-skeletal pathways of the oral nasal nerve (nNo) and superior premaxillary nerve (nPMs) are indicated with orange and blue squares, respectively, and the bony foramina connected to the premaxillary canal (cPM) are also shown with yellow triangles. Abbreviations: EN, external naris; nNi, internal nasal nerve; nNm, medial nasal nerve; nPMi, inferior premaxillary nerve; NpMx, nasal process of maxilla; ONT, ossified nasal turbinate.

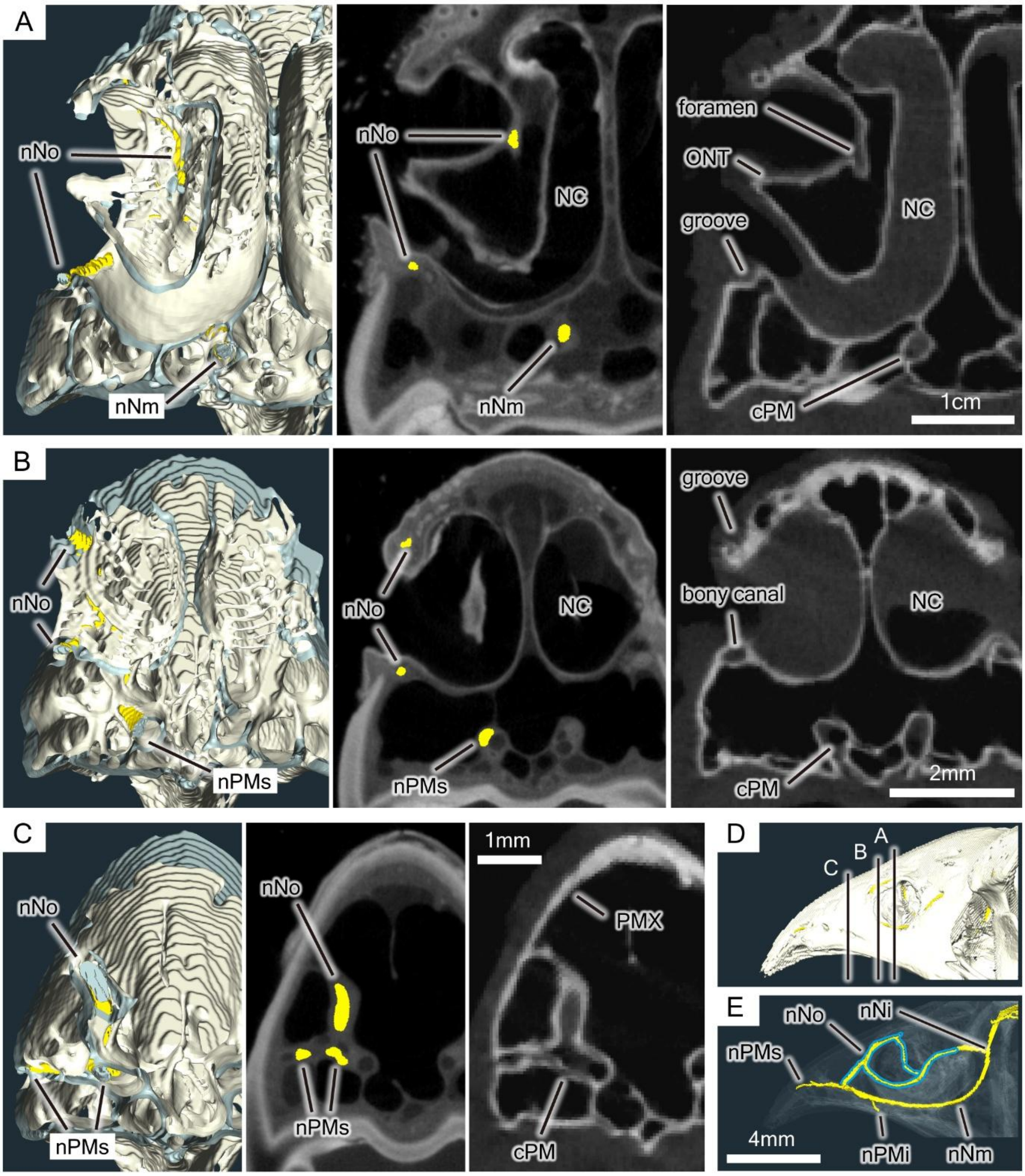

Figure S6.

The ophthalmic nerve pathway of *Falco tinnunculus* at the center of the external naris (A), anterior tip of the external naris (B), and posterior part of the snout tip (C).

In (A-C), 3D reconstructions of the nerve pathway, diceCT images and preCT images are shown in the left, middle and right columns. (D), Left lateral view of the snout region of the skull showing the position of the cross-sectioning area of (A-C). (e), Ophthalmic nerve pathway with the transparent skull image of (D). In (A-E), on the diceCT images, ophthalmic nerve branches are shown in yellow. In (E), the oral nasal nerve (nNo) is shown with blue line to distinguish from the internal nasal nerve (nNi). Abbreviations: cPM, premaxillary canal; NC, nasal cavity; nNm, medial nasal nerve; nNo, oral nasal nerve; nPMi, inferior premaxillary nerve; nPMs, superior premaxillary nerve; ONT, ossified nasal turbinate; PMX, premaxilla.

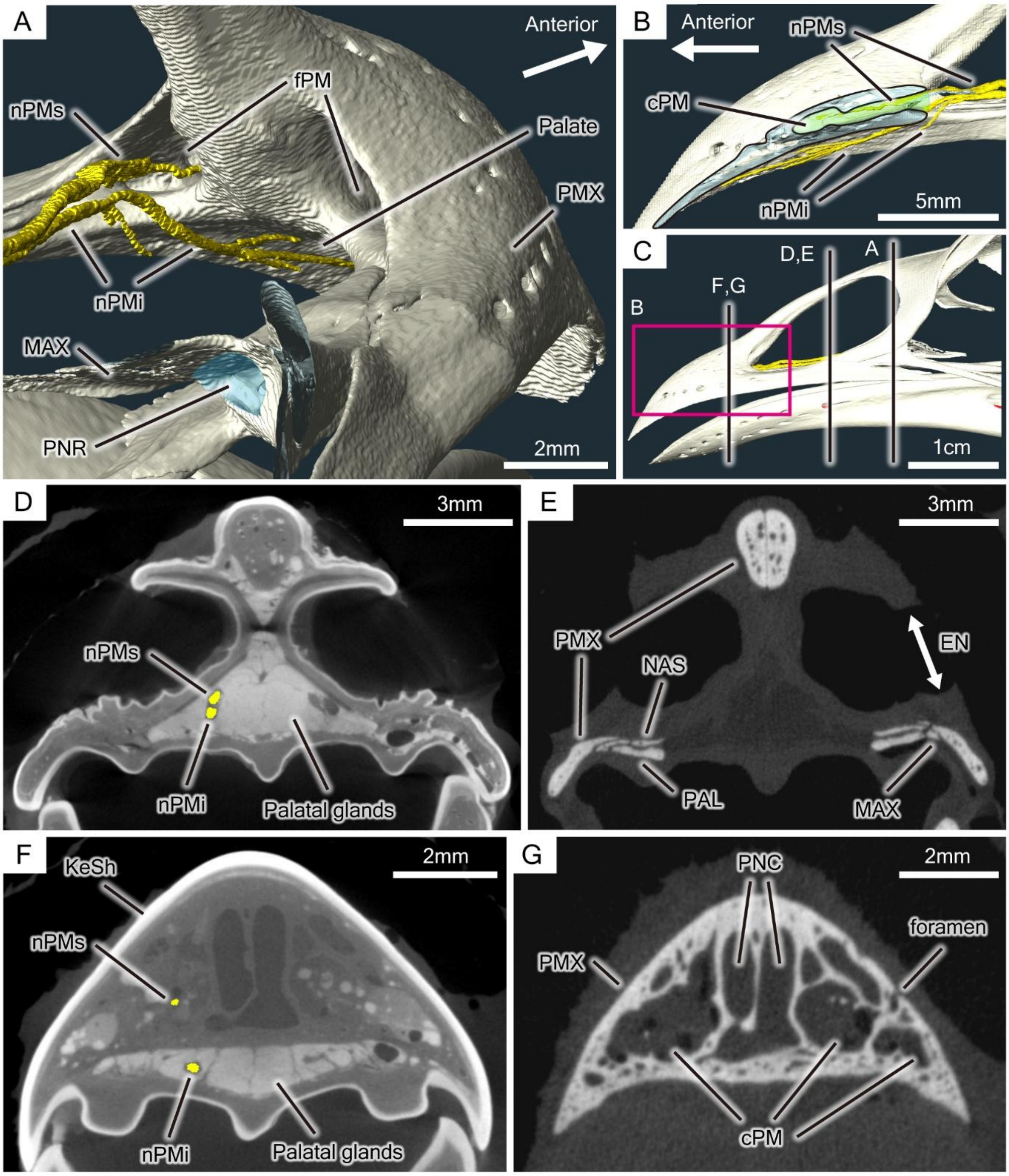

Figure S7.

The ophthalmic nerve pathway at the anterior part of the snout in *Pavo cristatus*.

(A), Right posterolateral view from the external naris (EN). (B), Sagittal section of the snout. (C), Left lateral view of the snout showing the cross-section area of (A-B) and (D-G). (D, E), Transverse section at the middle part of the external naris with diceCT and preCT images, respectively. (F, G), Transverse section at the anterior tip of the snout with diceCT and preCT images, respectively. In (A), the pneumatic recess (PNR) in the maxilla is shown in light blue. In (B), the cross-sectional edge of premaxilla and premaxillary canal are shown with black curved line and light green, respectively. In the diceCT images including (C, E), nerve branches of the ophthalmic nerve are shown in yellow. Abbreviations: cPM, premaxillary canal; fPM, premaxillary foramen; KeSh, keratinous sheath; MAX, maxilla; NAS, nasal; nPMi, inferior premaxillary nerve; nPMs, superior premaxillary nerve; PAL, palatine; PMX, premaxilla; PNC, pneumatic cavity.

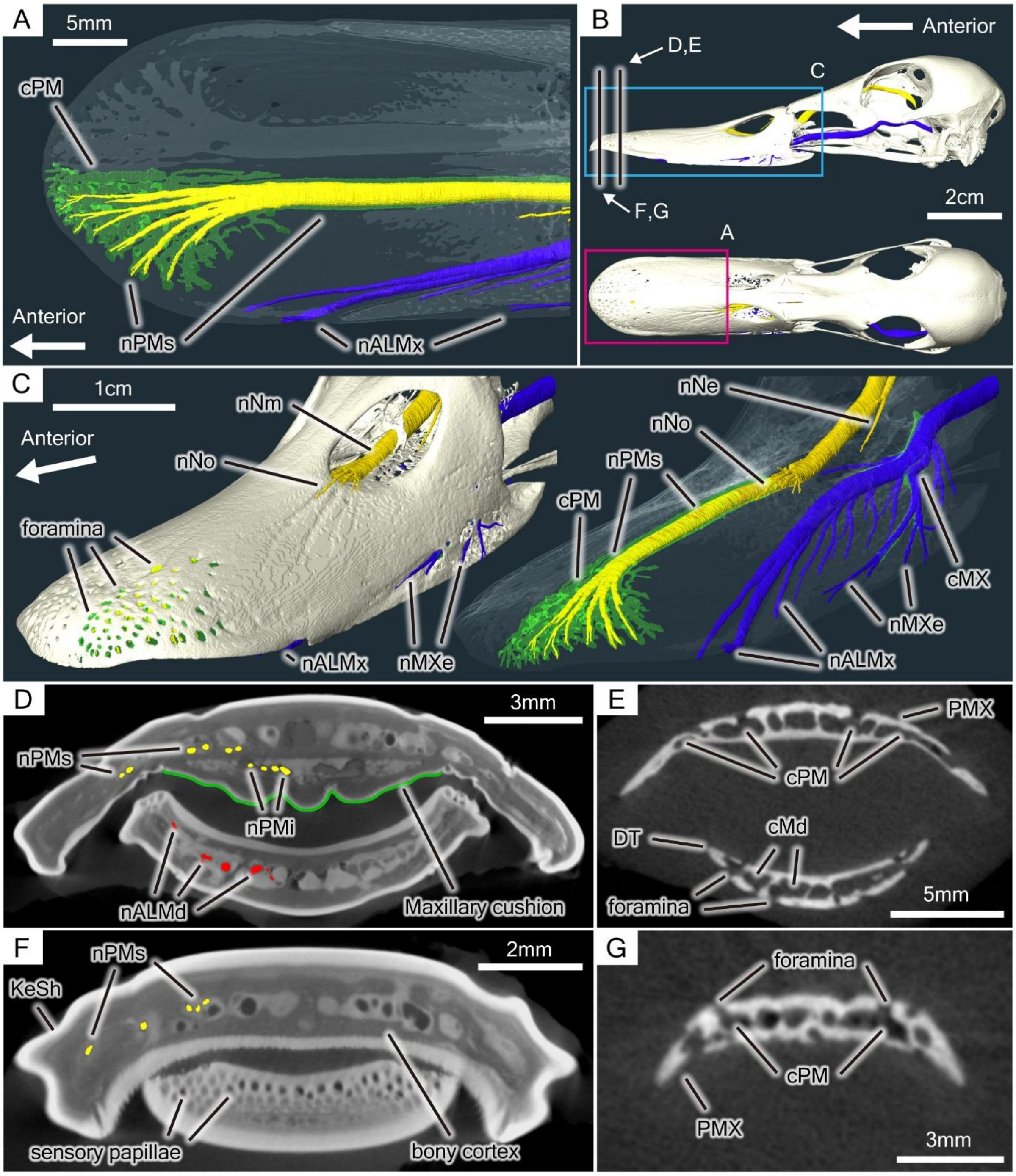

Figure S8.

The ophthalmic and maxillary nerve pathway around the bill tip of *Anas platyrhynchos.*

Note. (A), Nerve pathway in the dorsal view at the area indicated in (B). (B), Left lateral (top) and dorsal views (bottom) of the skull indicating the positions of cross sections (D-G) and areas shown in (A) and (C). (C), Nerve pathway in the anterolateral view at the area indicated in (B) in a solid (left) or transparent (right) skull. (D-G), Transverse diceCT images at the levels indicated in (B). The ophthalmic and mandibular nerve branches are shown in yellow and red dots, respectively, in (D, F). In (D), the area of the maxillary cushion is additionally shown in green. Abbreviations: cMd, mandibular canal; cMX, maxillary canal; cPM, premaxillary canal; DT, dentary; KeSh, keratinous sheath; nALMd, mandibular alveolar nerve; nALMx, maxillary alveolar nerve; nMXe, external maxillary branch; nNe, external nasal nerve; nNm, medial nasal nerve; nNo, oral nasal branch; nPMi, inferior premaxillary nerve; nPMs, superior premaxillary nerve; PMX, premaxilla.

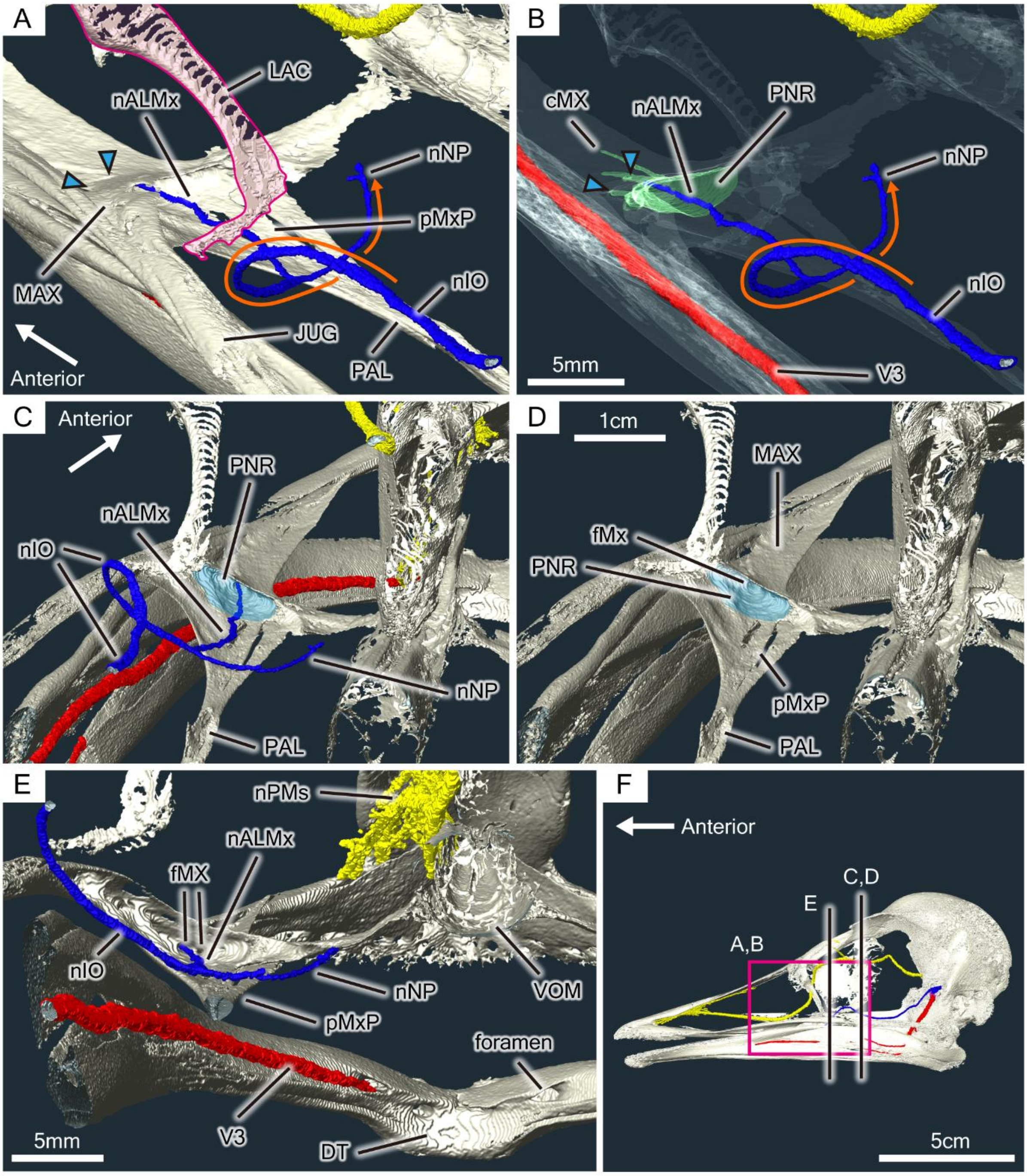

Figure S9.

The maxillary nerve pathway at the antorbital region of *Struthio camelus.*

Note. (A-E), Close-up views of the areas or levels indicated in (F). (A) and (B), Posterodorsal view with and without skull being transparent, respectively. The pathway of the nasopalatine nerve (nNP) around the maxillopalatine process (pMxP) is shown with orange arrows. In addition, bony foramina of the maxilla connecting the bony cavity of the pneumatic recess (PNR) are shown with blue arrows. (C, D), Posterodorsal view of the transverse section with and without nerve branches, respectively. Bony openings of the pneumatic recess are indicated in blue. (E), Posterior view of the transverse section. (F), Lateral view of skull showing the targeting areas (A-B) and levels of the cross sections (C-E). Abbreviations: cMX, maxillary canal; DT, dentary; fMx, maxillary foramen; JUG, jugal; LAC, lacrimal; MAX, maxilla; nALMx, maxillary alveolar nerve; nIO, infraorbital nerve; mPMs, superior premaxillary nerve; PAL, palatine; V3, mandibular nerve; VOM, vomer.

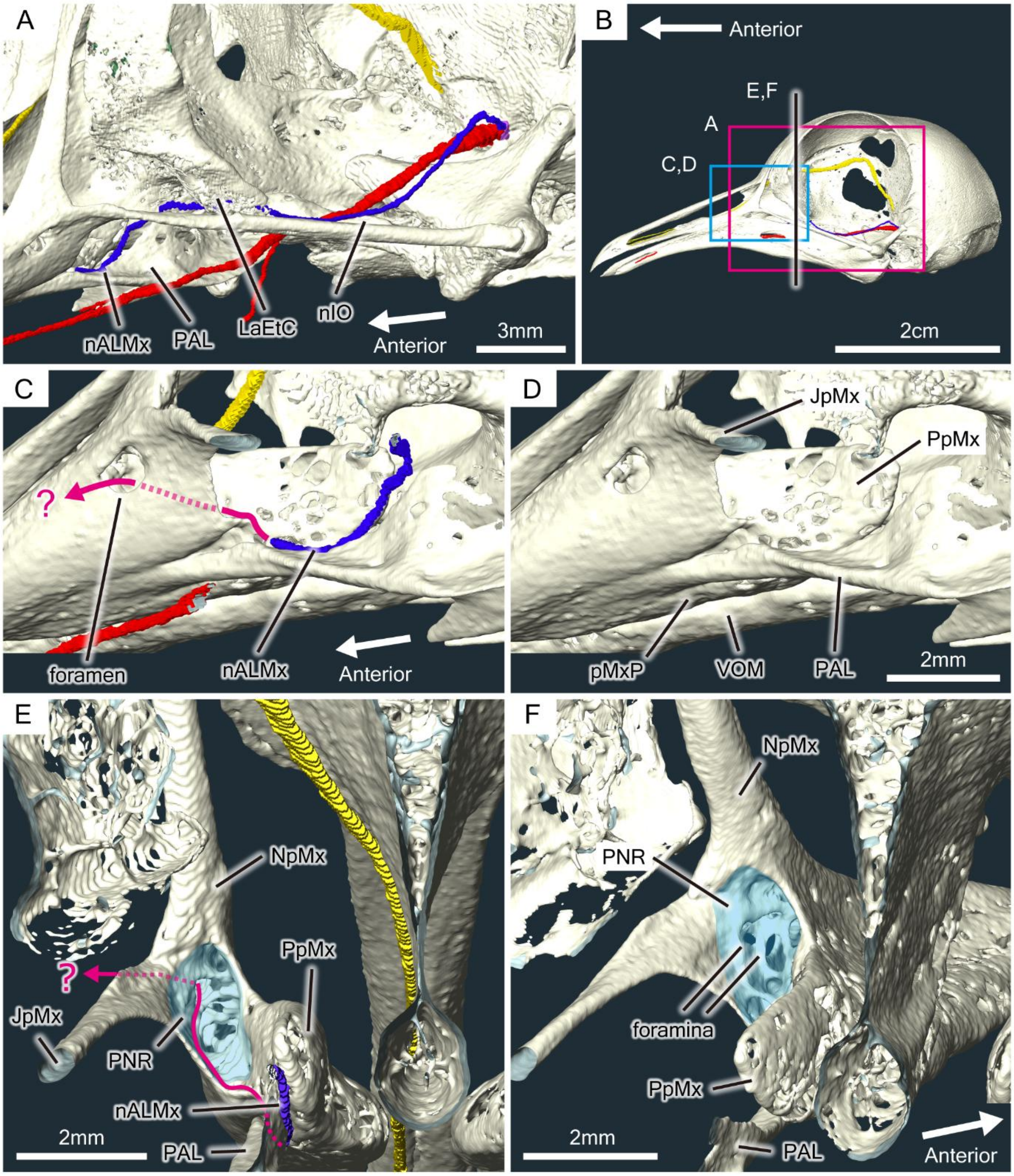

Figure S10.

The maxillary nerve pathway in the orbita of *Columba livia.*

(A), Left lateral view at the orbital region. (B), Lateral view of skull showing the targeting areas in (A, C, D). (C, D), Left posterolateral view of the maxilla with maxillary nerve and without nerve, respectively. (E), Posterior view of the transverse section of 3D reconstruction, at the area indicated in (B). (F), Right posterolateral view of the maxilla. In (E, F), the pneumatic recess of the maxilla (PNR) is shown in light blue. In (C, F), the hypothetical nerve pathway passing through the lateral maxillary foramen, based on the present study observation of the maxillary alveolar nerve (nALMx) is shown in pink lines. Abbreviations: JpMx, jugal process of maxilla; LaEtC, lacrimal-ethmoid bone complex; nIO, infraorbital nerve; NpMx, nasal process of maxilla; PAL, palatine; pMxP, maxillopalatine process; VOM, vomer.

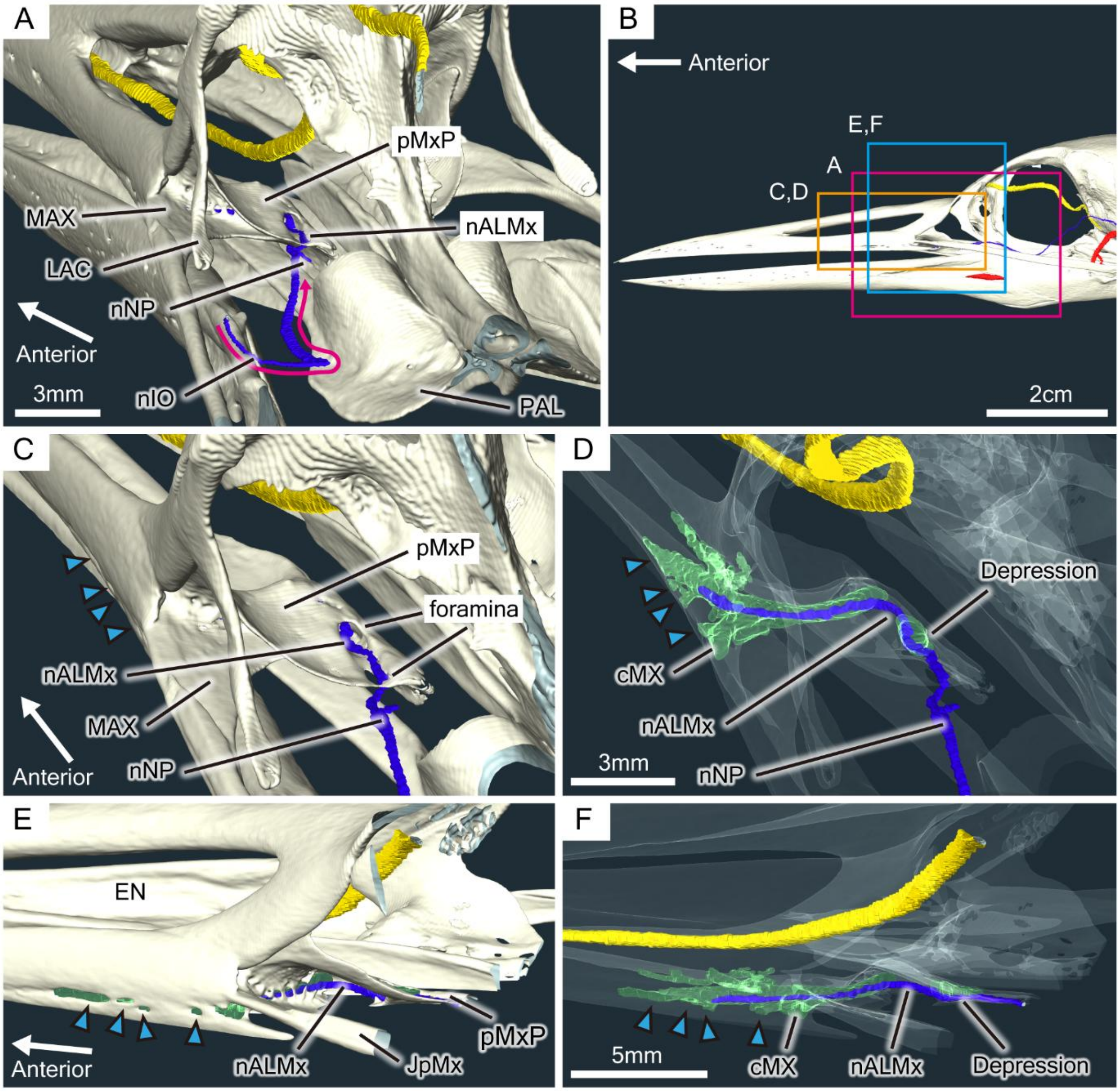

Figure S11.

The maxillary nerve pathway in the posterior beak region of *Podiceps cristatus.*

Note. (A, C-E), Close-up views of the areas indicated in (F). (A), Left posterolateral view. The pathway of infraorbital nerve (nIO) posterior to the maxillopalatine process (pMxP) is shown as a pink line with an arrowhead. (B), Lateral view of skull showing the targeting areas (A, C-F). (C, D), Left posterolateral view with and without the skull being transparent, respectively. (E, F), Left lateral view with and without the skull being transparent, respectively. In (C-F), the lateral maxillary foramen connecting the maxillary canal (cMX) are indicated with blue triangles. Abbreviations: EN, external naris; JpMx, jugal process of maxilla; LAC, lacrimal; MAX, maxilla; nALMx, maxillary alveolar nerve; nNP, nasopalatine nerve; PAL, palatine.

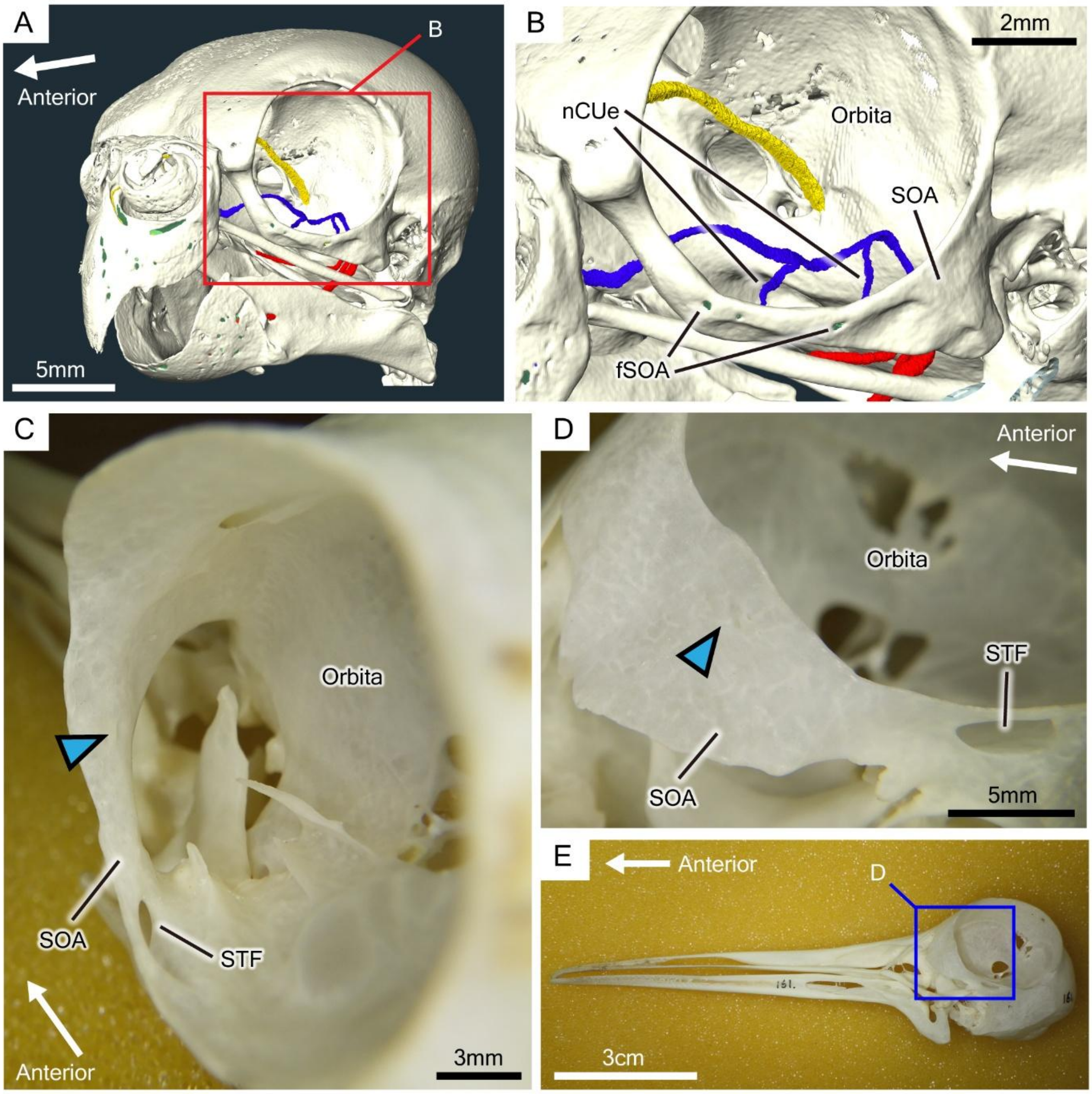

Figure S12.

The pathway of the external cutaneous nerve (nCUe) in *Melopsittacus* (A, B), and bony surface of *Scolopax rusticola* (NSM-PO-161) (C-E).

(A, B), Left anterolateral view of the skull. (C), Left posterolateral view of the suborbital arch (SOA). Left anteroventral view of the suborbital arch. In (A, B), bony foramina connecting to the bony canals are shown light green. In (C, D), the exit foramina of the suborbital arch (fSOA) are shown with blue triangles. In (E), the viewpoint of (D) is shown in the blue square. Abbreviation: STF, supratemporal fenestra.

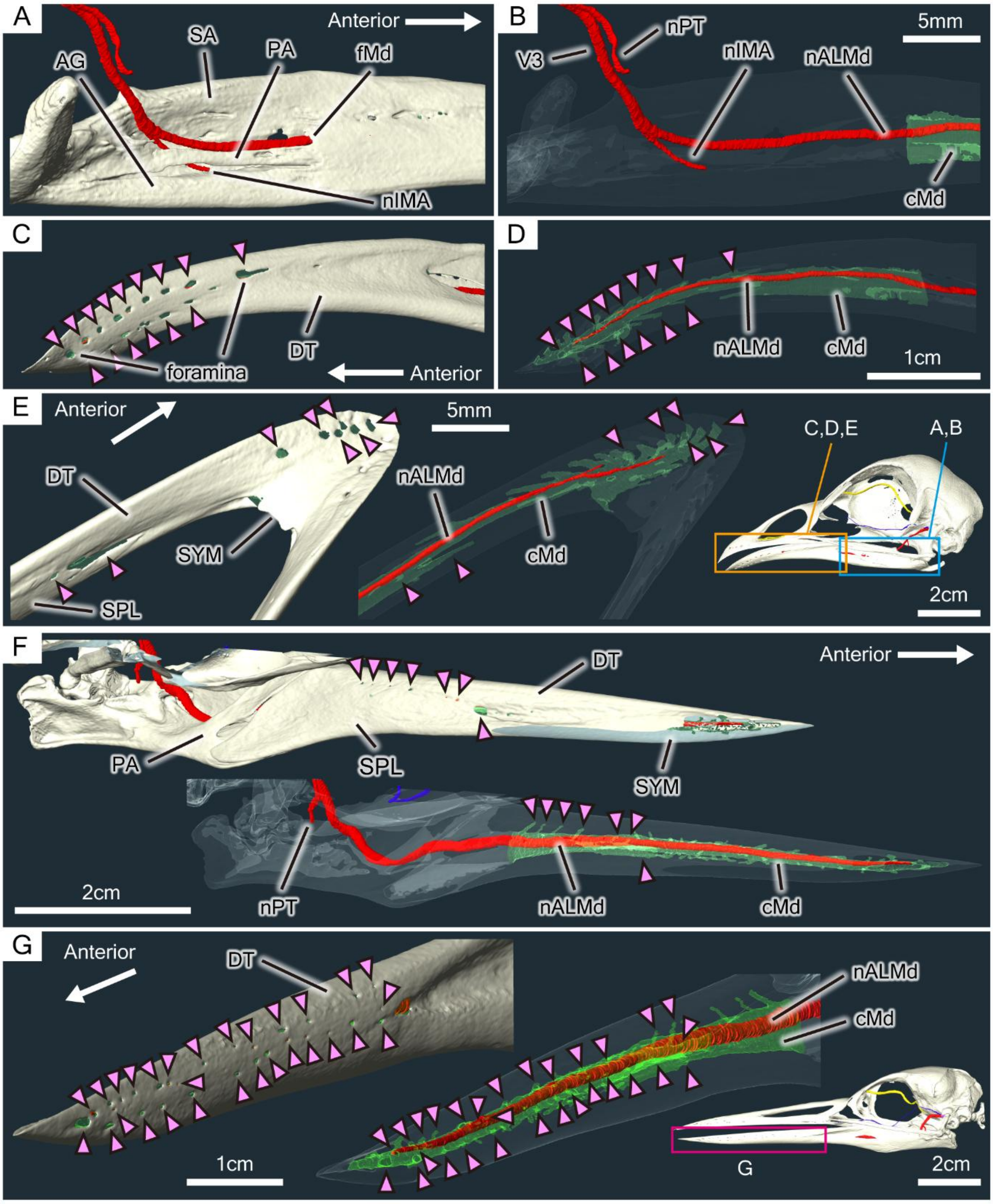

Figure S13.

The mandibular nerve pathway in the mandible of *Pavo cristatus* (A-E) and *Podiceps cristatus* (F, G).

Note. (A-E), Close-up views of the mandible in the areas indicated in the right-most inset figure of (E). (A, B), Medial view of the right ramus with and without the skull being transparent, respectively. (C, D), Left anterolateral view with and without the skull being transparent, respectively. (E), Right dorsolateral view of the mandibular tip with a solid (left) and transparent skull (center). (F), Left medial view of the whole mandible with a solid (top) and transparent skull (bottom). (G), Left anterolateral closeup view of the area indicated in the right-most inset figure, with a solid (left) and transparent skull (center). In (C-G), bony foramina connected to the mandibular canal (cMd) are indicated with pink

triangles. Abbreviations: AG, angular; DT, dentary; fMd, mandibular foramen; nALMd, mandibular alveolar nerve; nIMA, intermandibular nerve; nPT, pterygoid nerve; PA, prearticular; SA, surangular; SPL, splenial; SYM, mandibular symphysis; V3, mandibular nerve.

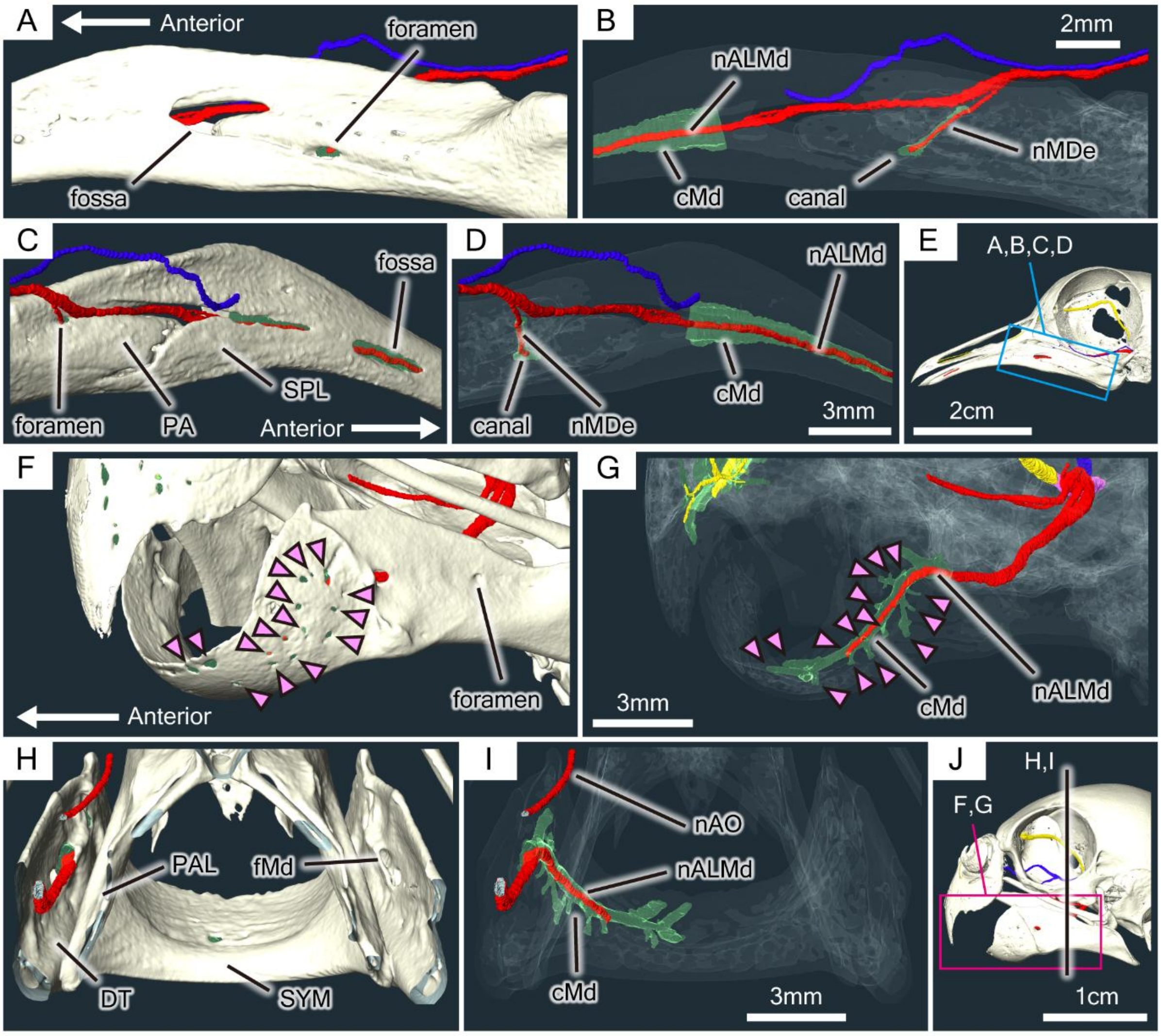

Figure S14.

Mandibular nerve pathway in the mandible of *Columba livia* (A-D) and *Melopsittacus undulatus* (F-J).

(A, B), Left lateral view of the middle part of the mandible with solid skull image and transparent skull image, respectively. (C, D), Same as (A, B), but from left medial view. (E), Left lateral view of the skull of *Columba*, showing the viewpoint of (A-D). (F, G), Same as (A, B), but in *Melopsittacus*. (G, H), Posterior view of the transverse section of the mandible with solid skull image and transparent skull image, respectively. (J), Left lateral skull of Melopsittacus, showing the viewpoint of (F, G), and cross-section area of (G, H). In (A-D, F-I), mandibular canals (cMd) are shown in light green. In (E, F), the bony foramina connected to the mandibular canal within the dentary is shown with pink triangles. Abbreviations: DT, dentary; fMd, mandibular foramen; nALMd, mandibular alveolar nerve; nAO, nerve for *anguli oris*; nMDe, external mandibular nerve; nSL, sublingual nerve; PA, prearticular; PAL, palatine; SPL, splenial; SYM, mandibular symphysis.

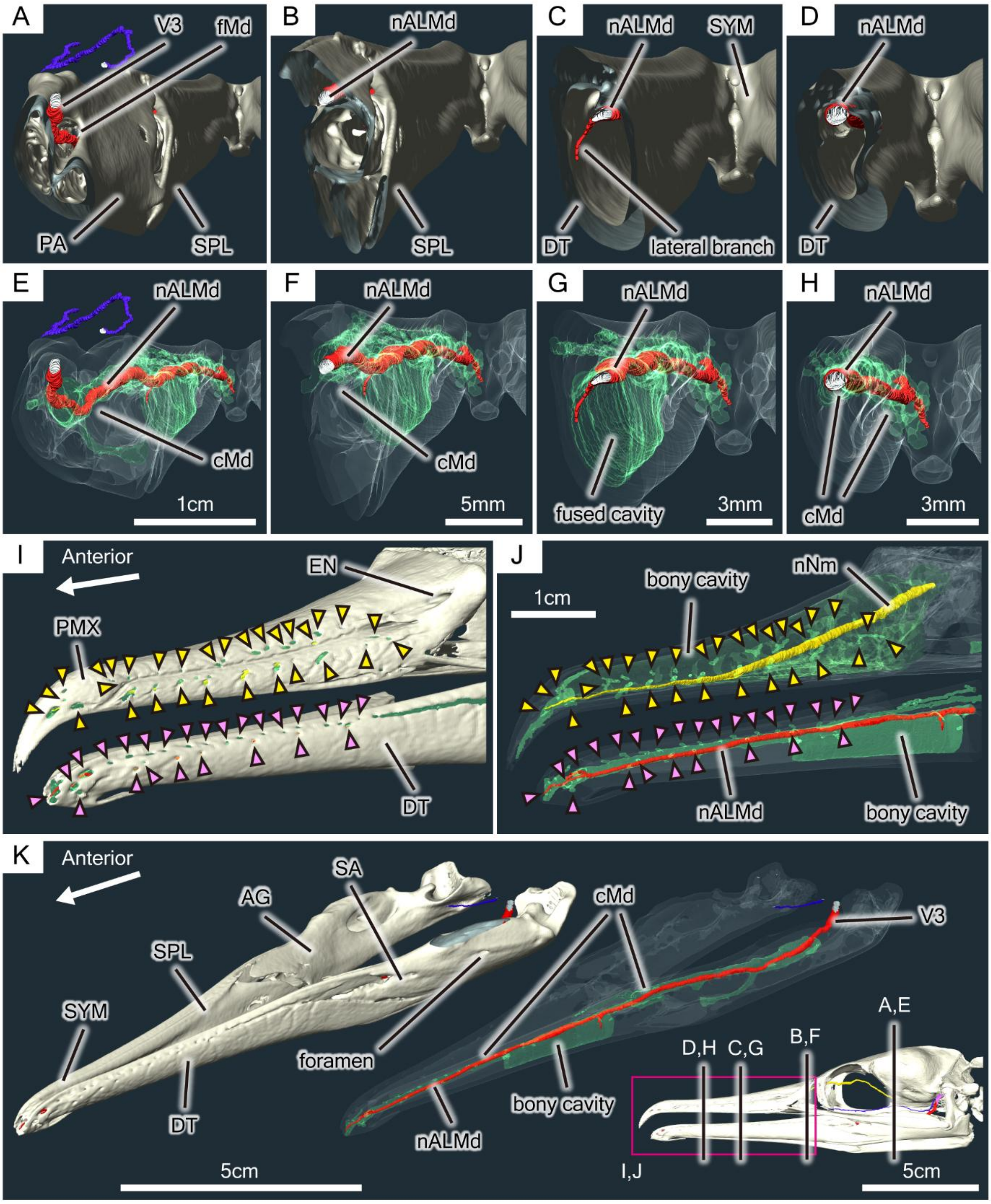

Figure S15.

The ophthalmic and mandibular nerve pathway in the skull of *Phalacrocorax carbo*.

(A-D), Posterior view of the transverse section of the 3D reconstruction of mandible. (E-H), Mandibular nerve pathway with the transparent skull image of (A-D). (I, J), Left anterolateral view of the snout with solid skull and transparent skull image, respectively. (K), Left anterolateral view of the mandibular nerve pathways with the solid skull image (left) and transparent skull image (middle). In (K), the areas of the transverse section of (A-F) are shown in the right inset figure. In (I, J), the bony foramina connected to the bony canals within the premaxilla and mandible are shown with yellow triangles and pink triangles, respectively. Abbreviations: AG, angular; cMd, mandibular canal; DT, dentary; EN, external naris; fMd, mandibular foramen; nALMd, mandibular alveolar nerve; nMDe, external mandibular nerve; nNm, medial nasal nerve; PA, prearticular; SA, surangular; SPL, splenial; SYM,

mandibular symphysis; PMX, premaxilla; V3, mandibular nerve.

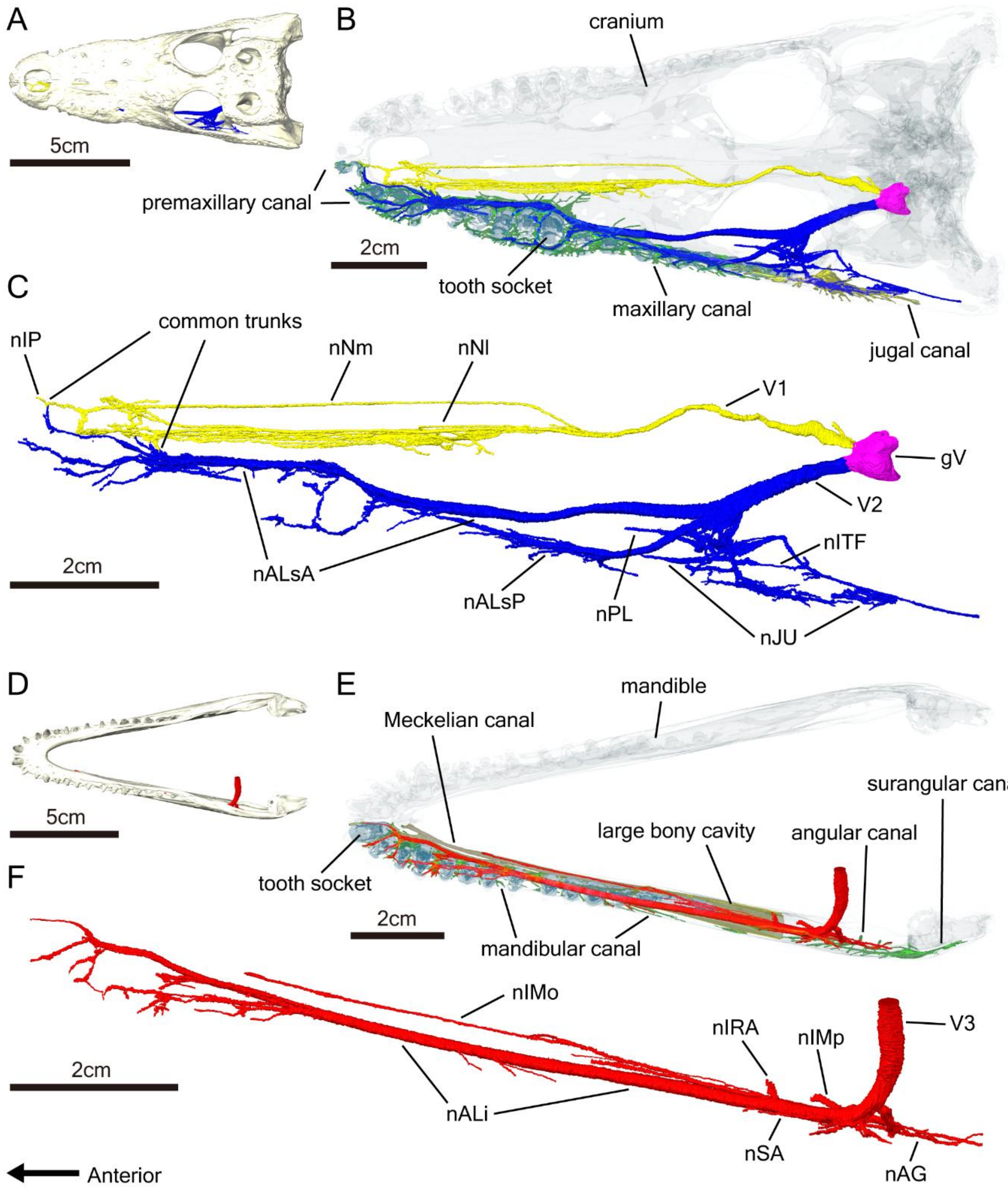

Figure S16.

3D reconstruction of the trigeminal nerve pathway in the upper jaw (A-C) and lower jaw (D-F) of *Crocodylus siamensis* in dorsal view.

(A, D), External view. (B, E), Internal view of nerve pathway. The trigeminal ganglion (gV) is shown in pink, the ophthalmic nerve (V1) in yellow, the maxillary nerve (V2) in blue, and the mandibular nerve (V3) in red. The neurovascular canal (NVC) and large bony cavities in the skull are shown in light green. The tooth sockets are shown in light blue. Abbreviations: nAG, angular branch; nALi, inferior alveolar nerve; nALsA, anterior division of the superior alveolar nerve; nALsP, posterior division of the superior alveolar nerve; nIMm, branch to the intramandibular muscle; nIMo, oral intermandibular branch; nIMp, caudal intermandibular branch; nIP, intrapremaxillary nerve; nITF, branch to infratemporal fenestra; nJU, jugal branch; nNl, lateral nasal nerve; nNm, medial nasal nerve; nPL, palatal branch; nSA, surangular branch.

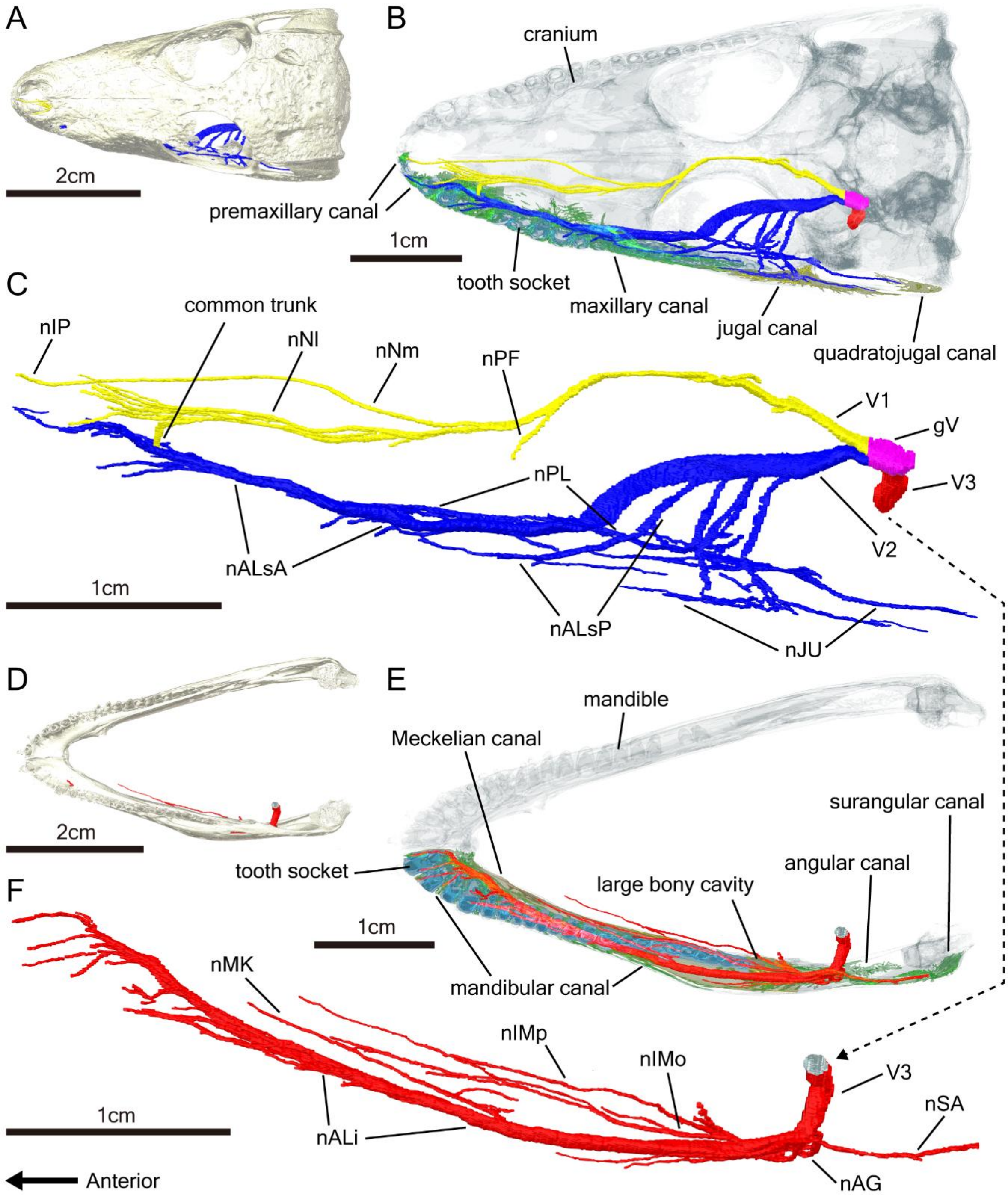

Figure S17.

3D reconstruction of the trigeminal nerve pathway in the upper jaw (A-C) and lower jaw (D-F) of *Caiman crocodilus* in dorsal view.

(A, D), External view. (B, E), Internal view of nerve pathway. The trigeminal ganglion (gV) is shown in pink, the ophthalmic nerve (V1) in yellow, the maxillary nerve (V2) in blue, and the mandibular nerve (V3) in red. The neurovascular canal (NVC) and large bony cavities in the skull are shown in light green. The tooth sockets are shown in light blue. The large mandibular cavities are shown in light yellow. Abbreviations: nAG, angular branch; nALi, inferior alveolar nerve; nALsA, anterior division of the superior alveolar nerve; nALsP, posterior division of the superior alveolar nerve; nIMo, oral intermandibular branch; nIMp, caudal intermandibular branch; nIP, intrapremaxillary nerve; nJU, jugal branch; nMK, Meckelian branch; nNl, lateral nasal nerve; nNm, medial nasal nerve; nPF, branch to the posterior part of prefrontal; nPL, palatal branch; nSA, surangular branch.

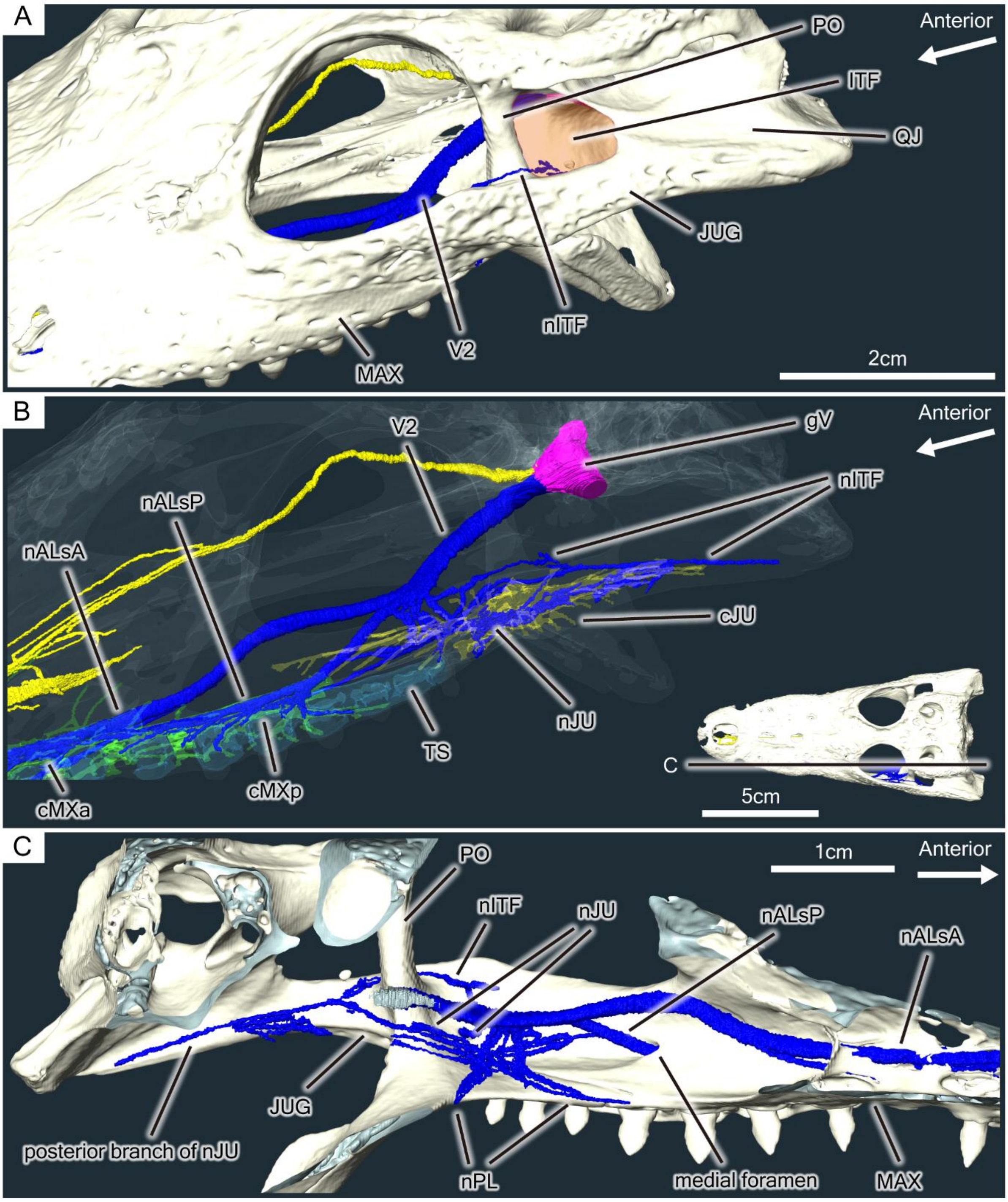

Figure S18.

Maxillary nerve pathways in the posterior part of the cranium of *Crocodylus siamensis*.

Note. (A-B), Left anterolateral view of the upper jaw around the jugal with and without the skull being transparent, respectively. The orange area in (A) indicates the infratemporal fenestra (ITF). The jugal canal is shown in light yellow to distinguish it from maxillary canals in (B). (C), Left part of the skull sagittally-sectioned at the level indicated in the right inset figure in (B). Abbreviations: cMXa, anterior part of the maxillary canal; cMXp, posterior part of the maxillary canal; gV, trigeminal ganglion; JUG, jugal; MAX, maxilla; nALsA, anterior division of superior alveolar nerve; nALsP, posterior division of the superior alveolar nerve; nITF, branch to infratemporal fenestra; nJU, jugal branch; nPL, palatal branch; PO, postorbital; QJ, quadratojugal; TS, tooth socket; V2, maxillary nerve.

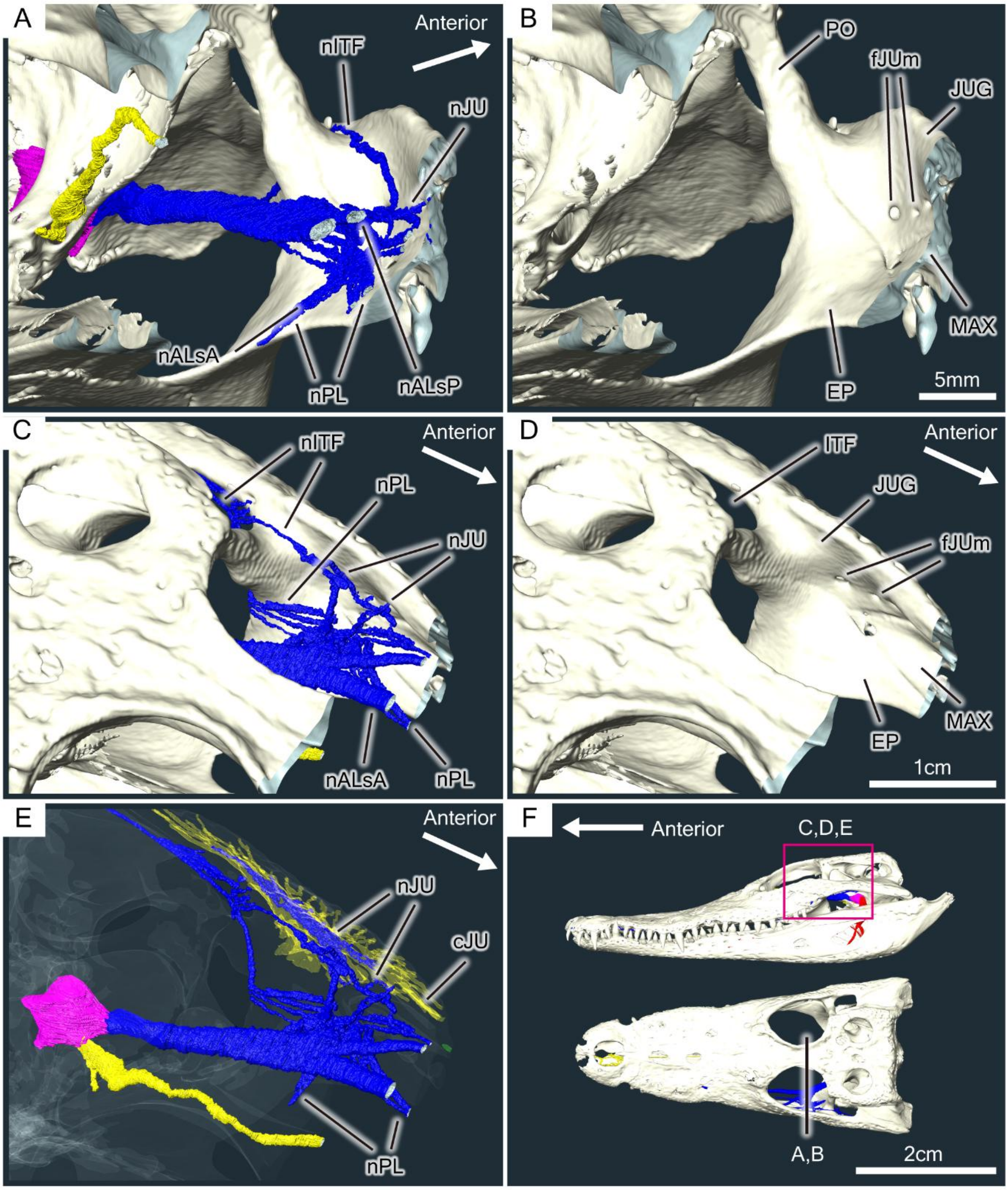

Figure S19.

The maxillary nerve pathway at the orbital region of *Crocodylus siamensis.*

(A), Anterior view of the transverse section at the orbital region. (B), Same as (A) but without nerve branches. (C), Right anterolateral view of the orbital region. (D), Same as (C) but without nerve branches. (E), Same as (C) but with transparent skull image. (F), Left lateral view of the skull (top) and dorsal view (bottom), showing cross-section area of (A, B) and viewpoint of (C-E). In (C), the jugal canal (cJU) is shown in light yellow. Abbreviations: EP, epipterygoid; fJUm, medial jugal foramen; ITF, infratemporal fenestra; JUG, jugal; MAX, maxilla; nALsA, anterior division of the superior alveolar nerve; nALsP, posterior division of the superior alveolar nerve; nITF, branch for infratemporal fenestra; nJU, jugal branch; nPL, palatal branch; PO, postorbital.

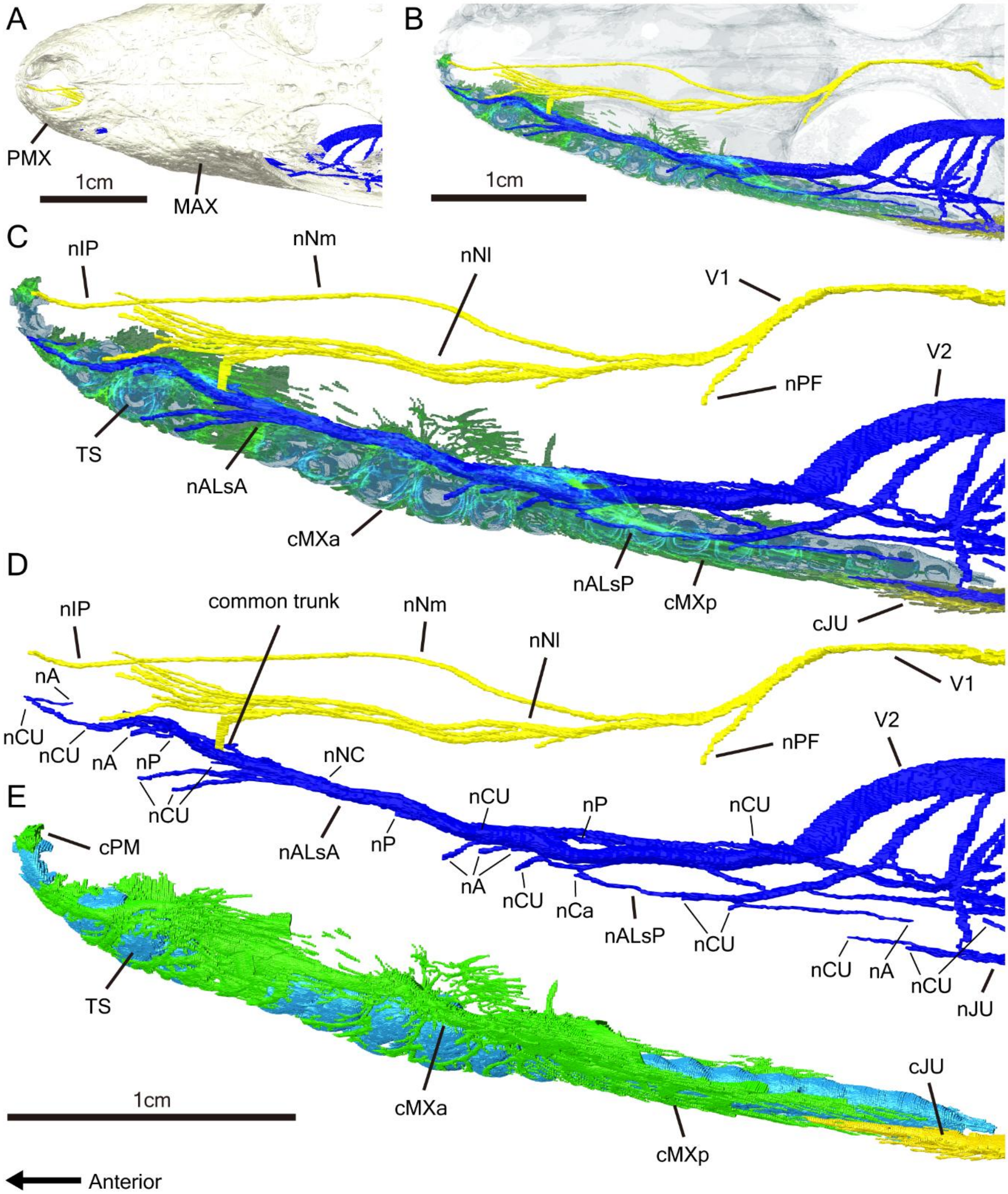

Figure S20.

The ophthalmic and maxillary nerve pathway at the snout region of *Caiman crocodilus* in dorsal view.

(A), External view. (B), Internal view of nerve pathway. (C), Nerve pathway with transparent image of bony canals (light green) and tooth sockets (light blue). (D), Only nerve pathway. (E), Solid image of bony canals and tooth sockets. The viewpoint of (A-E) is shown in the bottom left image of the skull (left lateral view). Abbreviations: cJU, jugal canal; cMXa, anterior part of maxillary canal; cMXp, posterior part of maxillary canal; cPM, premaxillary canal; MAX, maxilla; nA, branch to the tooth alveoli; nALsA, anterior division of the superior alveolar nerve; nALsP, posterior division of the superior alveolar nerve; nCa, branch with nerve ending confirmed within the canal; nCU, cutaneous branch; nIP, intrapremaxillary nerve; nJU, jugal branch; nNC, branch to nasal cavity; nNl, lateral nasal nerve; nNm, medial nasal nerve; nP, branch to palatal surface; nPF, branch to the posterior part of prefrontal; PMX, premaxilla; TS, tooth socket; V1, ophthalmic nerve; V2, maxillary nerve.

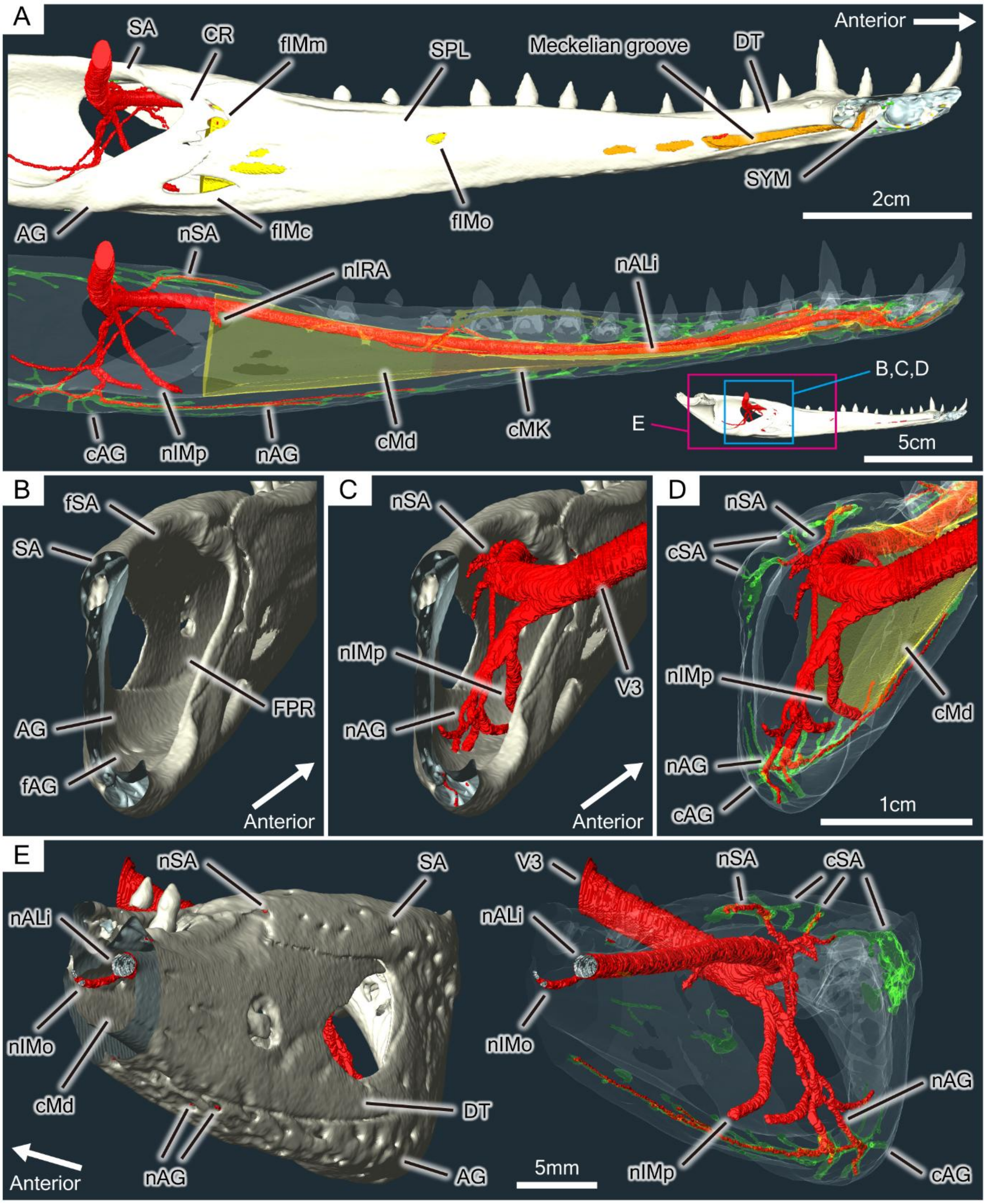

Figure S21.

Mandibular nerve pathway in the left ramus of the lower jaw of *Crocodylus siamensis*.

Note. (A), Medial view with a solid (top) and transparent skeleton (bottom). (B, C), Posteromedial view of the transverse section made posteriorly to the primordial fossa (FPR) at the level shown in the right bottom inset figure in (A), showing the skeleton only (B), solid skeleton with the mandibular nerve (C), and transparent skeleton with the mandibular nerve (D), respectively. (E), Anterolateral view of the area indicated in the right bottom inset figure in (A), with a solid (left) and transparent skull (right) In (A, D, E), thin bony canals, the large mandibular canal (cMd), Meckelian canal (cMK), and tooth sockets (TS) are shown in light green, light yellow, light orange and light blue, respectively. Abbreviations: AG, angular; cAG, angular canal; CR, coronoid; cSA, surangular canal; DT, dentary; fAG, angular foramen;

fIMc, caudal intermandibular foramen; fIMm, medial intermandibular foramen; fIMo, oral intermandibular foramen; fSA, surangular foramen; nAG, angular branch; nALi, inferior alveolar nerve; nIMm, branch to the intramandibular muscle; nIMo, oral intermandibular branch; nIMp; caudal intermandibular branch; nSA, surangular branch; SA, surangular; SPL, splenial; SYM, mandibular symphysis; V3, mandibular nerve.

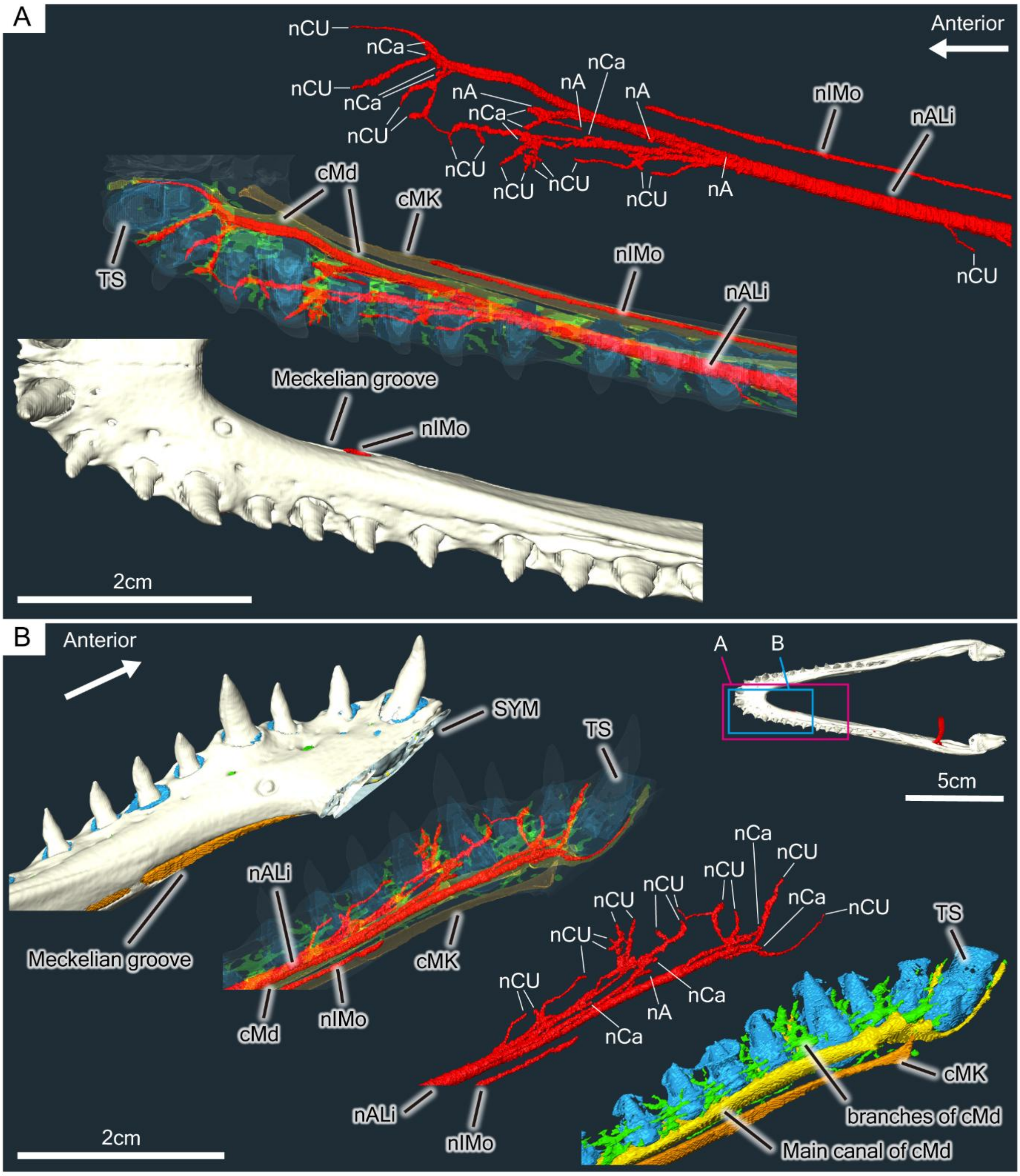

Figure S22.

The mandibular nerve pathway at the anterior tip of the mandible of *Crocodylus siamensis*.

(A), Dorsal view of the mandibular nerve (top), nerve branch with transparent skull image (middle), and with solid skull image (bottom). (B), Left dorsomedial view of the mandibular nerve pathway with solid skull image (top), with transparent skull image (top middle), only nerve pathway (bottom middle), and only solid image of bony canals and tooth sockets (bottom). In (B), the viewpoints of (A, B) are shown in the right column. The thin mandibular canals, thick mandibular canals, Meckelian canal, and tooth sockets are shown in light green, light yellow, light orange, and light blue, respectively. Abbreviations: cMd, mandibular canal; cMK, Meckelian canal; nA, branch to the tooth alveoli; nALi, inferior alveolar nerve; nCa, branch with nerve ending confirmed within the canal; nCU, cutaneous branch; nIMo, oral intermandibular branch; SYM, mandibular symphysis; TS, tooth socket.

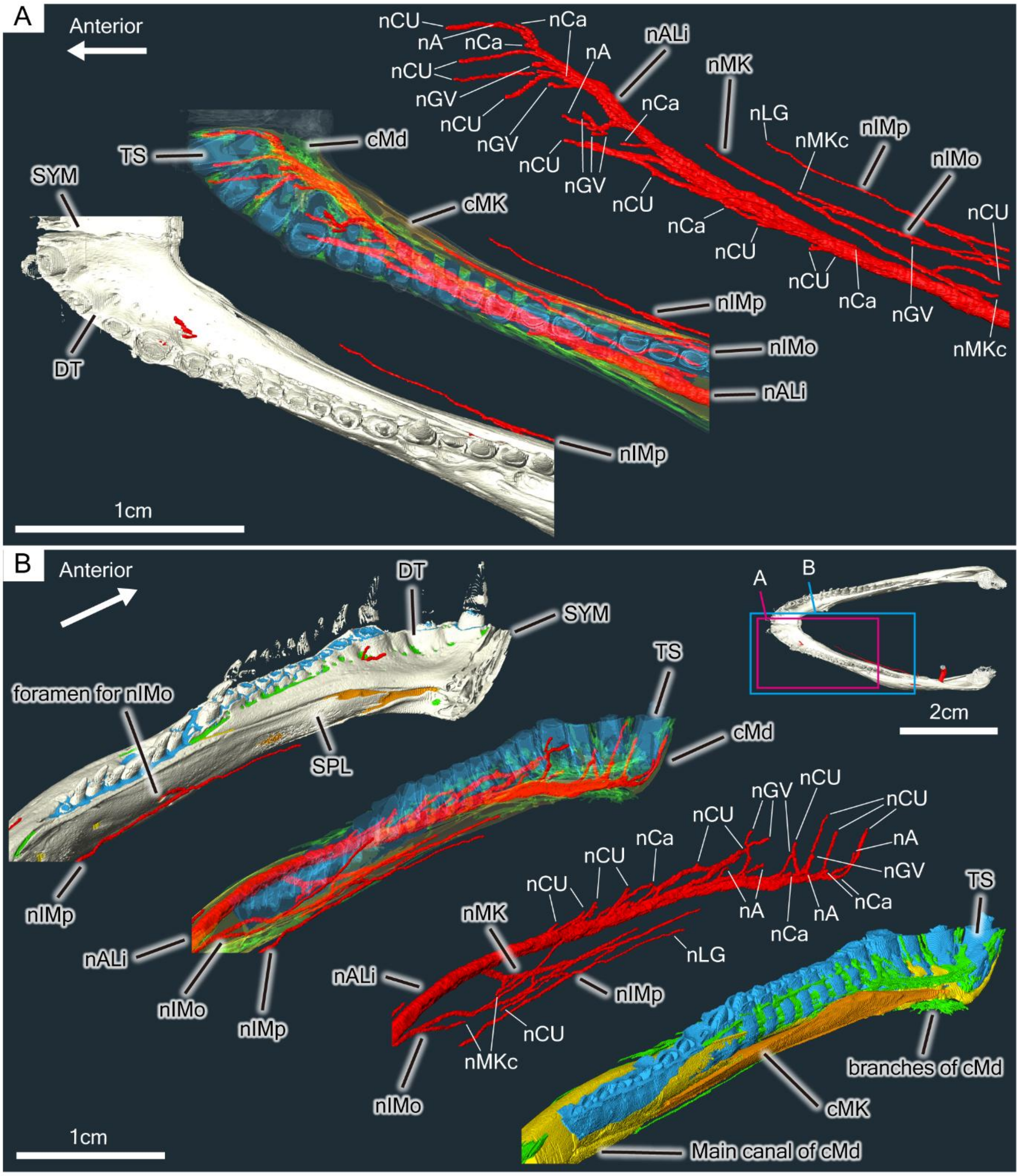

Figure S23.

The mandibular nerve pathway at the anterior tip of the mandible of *Caiman crocodylus*.

(A), Dorsal view of the mandibular nerve (top), nerve branch with transparent skull image (middle), and with solid skull image (bottom). (B), Left dorsomedial view of the mandibular nerve pathway with solid skull image (top), with transparent skull image (top middle), only nerve pathway (bottom middle), and only solid image of bony canals and tooth sockets (bottom). In (B), the viewpoints of (A, B) are shown in the right column. The thin mandibular canals, thick mandibular canals, Meckelian canal, and tooth sockets are shown in light green, light yellow, light orange, and light blue, respectively. Abbreviations: cMd, mandibular canal; cMK, Meckelian canal; DT, dentary; nA, branch to the tooth alveoli; nALi, inferior alveolar nerve; nCa, branch with nerve ending confirmed within the canal; nCU, cutaneous branch; nGV, gingival branch; nIMo, oral intermandibular branch; nIMp, caural intermandibular branch; nMKc, branch with nerve ending confirmed in the Meckelian canal; SPL, splenial; SYM, mandibular symphysis; TS, tooth socket.

Table S1. Used specimen for this study.

Taxon	Species	Specimen ID	Growth stages (W: wild, D: domestic)	Sex	Body length (mm)	Cranial length (mm)	Usage	Shown in figures	Taken from
Crocodylia									
Alligatoridae	*Caiman crocodilus*	Caiman 1	juvenile (D)	U	156	53.8	preCT + diceCT	✓	iZoo (Kamo-gun, Shizuoka)
Crocodylidae	*Crocodylus siamensis*	Crocodylus 1	juvenile (D)	U	-	146.4	preCT + diceCT	✓	stored in Tsuihiji laboratory (taken from domestic breeder)
		Crocodylus 2	juvenile (D)	U	698	142	preCT		stored in Tsuihiji laboratory (taken from domestic breeder)
		Crocodylus 3	juvenile (D)	U	741	142.5	preCT		stored in Tsuihiji laboratory (taken from domestic breeder)
		Crocodylus 4	juvenile (D)	U	968	157.5	preCT		stored in Tsuihiji laboratory (taken from domestic breeder)
Palaeognathae									
Struthioniformes	*Struthio camelus*	Struthio 1	juvenile (4 months, D)	F	-	151.9	preCT + diceCT	✓	Oyama Ostrich Farm (Oyama-city, Tochigi)
		Struthio 2	juvenile (6 months, D)	F	-	157	preCT		Oyama Ostrich Farm (Oyama-city, Tochigi)
		Struthio 3	juvenile (12 months, D)	M	-	195.8	preCT		Oyama Ostrich Farm (Oyama-city, Tochigi)
Galloanserae									
Galliformes	*Pavo cristatus*	Pavo 1	subadult (W)	M	1016	90.2	preCT + diceCT		Noblesse Oblige Co., Ltd. (Chikusei-city, Ibaraki)
		Pavo 2	subadult (W)	F	863	87.8	preCT + diceCT	✓	Noblesse Oblige Co., Ltd. (Chikusei-city, Ibaraki)
Anseriformes	*Anas platyrhynchos*	Anas 1	subadult (D)	U	-	143	preCT + diceCT		Shangmei Co., Ltd. (Nagoya-city, Aichi)
		Anas 2	subadult (D)	U	-	134.4	preCT		Shangmei Co., Ltd. (Nagoya-city, Aichi)
		Anas 3	adult (W)	M	589.9	121.1	preCT + diceCT	✓	Noblesse Oblige Co., Ltd. (Chikusei-city, Ibaraki)
		Anas 4	adult (W)	M	580.1	123.3	preCT		Noblesse Oblige Co., Ltd. (Chikusei-city, Ibaraki)
Neoaves									
Columbiformes	*Columba livia*	Columba 1	adult (D)	F	227	56.3	preCT + diceCT		Ryoushindou Co., Ltd. (Kasugai-shi, Aichi)
		Columba 2	adult (D)	M	244	57	preCT + diceCT		Ryoushindou Co., Ltd. (Kasugai-shi, Aichi)
		Columba 3	adult (W)	U	-	50.9	preCT + diceCT	✓	Dead body at The University of Tokyo (Bunkyo-ku, Tokyo)
Podicipediformes	*Podiceps cristatus*	Podiceps 1	adult (W)	M	530	108.2	preCT + diceCT	✓	Ouyo-Seibutsu Co., Ltd. (Minato-ku, Tokyo)
		Podiceps 2	adult (W)	F	537	101.6	preCT		Ouyo-Seibutsu Co., Ltd. (Minato-ku, Tokyo)
Suliformes	*Phalacrocorax carbo*	Phalacrocorax 1	adult (W)	F	797	136.7	preCT + diceCT	✓	Gunma prefecture fishery cooperative (Maebashi-city, Gunma)
Falconiformes	*Falco tinnunculus*	Falco 1	adult (W)	F	311	47.9	preCT + diceCT	✓	Ouyo-Seibutsu Co., Ltd. (Minato-ku, Tokyo)
		Falco 2	adult (W)	M	360	51.3	preCT		Ouyo-Seibutsu Co., Ltd. (Minato-ku, Tokyo)
Psittaciformes	*Melopsittacus undulatus*	Melopsittacus 1	subadult (D)	F	-	24.7	preCT + diceCT	✓	stored in Tsuihiji laboratory (taken from domestic breeder)
Passeriformes	*Turdus eunomus*	Turdus 1	adult (W)	M	180.5	49.8	preCT + diceCT		Dead body at The International Christian University (Mitaka-city, Tokyo)
		Turdus 2	adult (W)	M	233	49.4	preCT + diceCT	✓	Ouyo-Seibutsu Co., Ltd. (Minato-ku, Tokyo)
		Turdus 3	adult (W)	F	242	47.1	preCT + diceCT		Ouyo-Seibutsu Co., Ltd. (Minato-ku, Tokyo)

Table S2. Iodine staining method for diceCT imaging.

Specimen ID (See Table S1)	10% NBF fixation	70% EtOH preservation	20% Sucrose immersion	10% I2KI immersion	How to make 10% I2KI solution (3.3% I2 and 6.7% KI in water (w/v))
Crododylia					
Caiman 1	103 days	-	2 days	19 days	dilute 20% commercial Lugol's iodine solution
Crocodylus 1	> 2 years	7 days	2 days	20 days	dilute 20% commercial Lugol's iodine solution
Palaeognathae					
Struthio 1	29 days	-	2 days	33 days	dilute 20% commercial Lugol's iodine solution
Galloanserae					
Pavo 1	64 days	-	-	72 days	grind iodine granules and mix with KI powder in tapped water
Pavo 2	513 days	-	-	44 days	grind iodine granules and mix with KI powder in tapped water
Anas 1	142 days	-	2 days	26 days	dilute 20% commercial Lugol's iodine solution
Anas 3	522 days	-	-	36 days	grind iodine granules and mix with KI powder in tapped water
Neoaves					
Columba 1	150 days	-	2 days	26 days	dilute 20% commercial Lugol's iodine solution
Columba 2	189 days	-	2 days	22 days	dilute 20% commercial Lugol's iodine solution
Columba 3	156 days	-	-	17 days	grind iodine granules and mix with KI powder in tapped water
Podiceps 1	64 days	-	-	33 days	grind iodine granules and mix with KI powder in tapped water
Phalacrocorax 1	75 days	-	2 days	25 days	dilute 20% commercial Lugol's iodine solution
Falco 1	64 days	-	-	25 days	grind iodine granules and mix with KI powder in tapped water
Melopsittacus 1	-	> 1 year	-	17 days	grind iodine granules and mix with KI powder in tapped water
Turdus 1	79 days	-	2 days	27 days	dilute 20% commercial Lugol's iodine solution
Turdus 2	64 days	-	-	20 days	grind iodine granules and mix with KI powder in tapped water
Turdus 3	61 days	-	-	17 days	grind iodine granules and mix with KI powder in tapped water

Table S3. preCT scanning method for this study.

The institution abbreviations are as follows: National Museum of Nature and Science (NMNS), The University of Tokyo (UT).

Specimen ID (See Table S1)	Used in diceCT	CT scanner	Institute	Views	Rotation degree	Average	XV Voxel (um)	Z spacing (um)	Image pixel	Voltage (kV)	Current (uA)	Filter
Crocodylia												
Caiman 1	✓	TXS320-ACTIS (TESCO)	NMNS (Taito-ku, Tokyo)	1000	360	10	108.4	100	1024 x 1024	168	200	Cu 0.5mm
Crocodylus 1	✓	TXS320-ACTIS (TESCO)	NMNS (Taito-ku, Tokyo)	1000	360	10	84.96	100	1024 x 1024	145	200	Cu 0.5mm
Crocodylus 2		inspeXio SMX-225CT FPD HR (SHIMADZU)	NMNS (Tsukuba-city, Ibaraki)	600	360	2	69.37	122	1024 x 1024	225	70	-
Crocodylus 3		inspeXio SMX-225CT FPD HR (SHIMADZU)	NMNS (Tsukuba-city, Ibaraki)	1200	360	3	64.97	129	512 x 512	200	200	-
Crocodylus 4		inspeXio SMX-225CT FPD HR (SHIMADZU)	NMNS (Tsukuba-city, Ibaraki)	1200	360	3	81.02	77	1024 x 1024	200	200	-
Palaeognathae												
Struthio 1	✓	TXS320-ACTIS (TESCO)	NMNS (Taito-ku, Tokyo)	1000	360	10	216.8	100	1024 x 1024	207	200	Cu 0.5mm
Struthio 2		TXS320-ACTIS (TESCO)	NMNS (Taito-ku, Tokyo)	1000	360	10	216.8	100	1024 x 1024	207	200	Cu 0.5mm
Struthio 3		inspeXio SMX-225CT FPD HR (SHIMADZU)	NMNS (Tsukuba-city, Ibaraki)	1200	360	4	134.71	248	1024 x 1024	225	70	-
Galloanserae												
Pavo 1	✓	SkyScan 1173 (BRUKER)	UT (Bunkyo-ku, Tokyo)	800	240	3	24.94	24.94	2240 x 2240	130	55	-
Pavo 2	✓	SkyScan 1173 (BRUKER)	UT (Bunkyo-ku, Tokyo)	800	240	3	26.01	26.01	2240 x 2240	130	55	-
Anas 1	✓	TXS320-ACTIS (TESCO)	NMNS (Taito-ku, Tokyo)	1000	360	10	140.63	100	1536 x 1536	146	200	Cu 0.5mm
Anas 2		TXS320-ACTIS (TESCO)	NMNS (Taito-ku, Tokyo)	1000	360	10	140.63	100	1536 x 1536	146	200	Cu 0.5mm
Anas 3	✓	SkyScan 1173 (BRUKER)	UT (Bunkyo-ku, Tokyo)	685	240	3	54.15	54.15	1120 x 1120	110	55	-
Anas 4		SkyScan 1173 (BRUKER)	UT (Bunkyo-ku, Tokyo)	685	240	3	54.86	54.86	1120 x 1120	130	55	-
Neoaves												
Columba 1	✓	TXS320-ACTIS (TESCO)	NMNS (Taito-ku, Tokyo)	1000	360	10	168.95	100	1024 x 1024	146	200	Cu 0.5mm
Columba 2	✓	TXS320-ACTIS (TESCO)	NMNS (Taito-ku, Tokyo)	1000	360	10	168.95	100	1024 x 1024	146	200	Cu 0.5mm
Columba 3	✓	SkyScan 1173 (BRUKER)	UT (Bunkyo-ku, Tokyo)	600	240	3	19.62	19.62	2240 x 2240	130	55	-
Podiceps 1	✓	SkyScan 1173 (BRUKER)	UT (Bunkyo-ku, Tokyo)	685	240	3	23.87	23.87	2240 x 2240	130	55	-
Podiceps 2		SkyScan 1173 (BRUKER)	UT (Bunkyo-ku, Tokyo)	685	240	3	23.16	23.16	2240 x 2240	130	55	-
Phalacrocorax 1	✓	TXS320-ACTIS (TESCO)	NMNS (Taito-ku, Tokyo)	900	360	9	53.71	100	1024 x 1024	146	200	Cu 0.5mm
Falco 1	✓	SkyScan 1173 (BRUKER)	UT (Bunkyo-ku, Tokyo)	800	240	3	22.09	22.09	2240 x 2240	80	70	Al 1.0mm
Falco 2		SkyScan 1173 (BRUKER)	UT (Bunkyo-ku, Tokyo)	800	240	3	18.88	18.88	2240 x 2240	90	85	Al 1.0mm
Melopsittacus 1	✓	SkyScan 1173 (BRUKER)	UT (Bunkyo-ku, Tokyo)	960	240	3	14.96	14.96	2240 x 2240	75	100	Al 1.0mm
Turdus 1	✓	TXS320-ACTIS (TESCO)	NMNS (Taito-ku, Tokyo)	1000	360	10	101.56	100	1024 x 1024	145	200	Cu 0.5mm
Turdus 2	✓	SkyScan 1173 (BRUKER)	UT (Bunkyo-ku, Tokyo)	800	240	3	17.1	17.1	2240 x 2240	80	70	Al 1.0mm
Turdus 3	✓	SkyScan 1173 (BRUKER)	UT (Bunkyo-ku, Tokyo)	800	240	3	17.1	17.1	2240 x 2240	130	55	-

Table S4. diceCT scanning method for this study.

The institution abbreviations are as follows: National Museum of Nature and Science (NMNS), The University of Tokyo (UT).

Specimen ID (See Table S1)	iodine staining	CT scanner	Institute	Views	Rotation degree	Average	XY voxel (um)	Z spacing (um)	Image pixel	Voltage (kV)	Current (uA)	Filter	Offset scan	Shown in figures
Crocodylia														
Caiman 1	19 days	TXS320-ACTIS (TESCO)	NMNS (Taito-ku, Tokyo)	1000	360	10	34.18	50	1024 x 1024	174	200	Cu 0.5mm		✓
Crocodylus 1	20 days	TXS320-ACTIS (TESCO)	NMNS (Taito-ku, Tokyo)	1000	360	10	68.36	50	1024 x 1024	175	200	Cu 0.5mm		✓
Palaeognathae														
Struthio 1	33 days	TXS320-ACTIS (TESCO)	NMNS (Taito-ku, Tokyo)	1000	360	10	103.52	100	1024 x 1024	160	200	Cu 0.5mm		✓
Galloanserae														
Pavo 1	72 days	TXS320-ACTIS (TESCO)	NMNS (Taito-ku, Tokyo)	1000	360	10	29.3	50	2048 x 2048	175	200	Cu 0.5mm		
Pavo 2	44 days	inspeXio SMX-225CT FPD HR (SHIMADZU)	NMNS (Tsukuba-city, Ibaraki)	1200	360	1	24.57	22	2048 x 2408	200	70	-		✓
Anas 1	26 days	TXS320-ACTIS (TESCO)	NMNS (Taito-ku, Tokyo)	1000	360	10	62.5	50	1024 x 1024	175	200	Cu 0.5mm		
Anas 3	36 days	inspeXio SMX-225CT FPD HR (SHIMADZU)	NMNS (Tsukuba-city, Ibaraki)	1200	360	2	22.71	23	2048 x 2408	195	70	-		✓
Noeaves														
Columba 1	26 days	TXS320-ACTIS (TESCO)	NMNS (Taito-ku, Tokyo)	1000	360	10	42.97	50	1024 x 1024	175	200	Cu 0.5mm		
Columba 2	22 days	TXS320-ACTIS (TESCO)	NMNS (Taito-ku, Tokyo)	1000	360	10	44.92	50	1024 x 1024	175	200	Cu 0.5mm		
Columba 3	17 days	SkyScan 1173 (BRUKER)	UT (Bunkyo-ku, Tokyo)	960	240	5	17.46	17.46	2240 x 2240	120	66	Brass 0.25mm		✓
Podiceps 1	33 days	SkyScan 1173 (BRUKER)	UT (Bunkyo-ku, Tokyo)	1200	240	5	16.03	16.03	2240 x 2240	120	66	Brass 0.25mm		✓
Phalacrocorax 1	25 days	TXS320-ACTIS (TESCO)	NMNS (Taito-ku, Tokyo)	1000	360	10	53.71	100	1024 x 1024	175	200	Cu 0.5mm		✓
Falco 1	25 days	SkyScan 1173 (BRUKER)	UT (Bunkyo-ku, Tokyo)	1200	240	5	16.39	16.39	2240 x 2240	120	66	Brass 0.25mm		✓
Melopsittacus 1	17 days	SkyScan 1173 (BRUKER)	UT (Bunkyo-ku, Tokyo)	960	240	5	12.83	12.83	2240 x 2240	120	66	Brass 0.25mm	✓	✓
Turdus 1	27 days	TXS320-ACTIS (TESCO)	NMNS (Taito-ku, Tokyo)	1000	360	10	53.71	50	1024 x 1024	175	200	Cu 0.5mm		
Turdus 2	20 days	SkyScan 1173 (BRUKER)	UT (Bunkyo-ku, Tokyo)	1200	240	5	14.25	14.25	2240 x 2240	120	66	Brass 0.25mm		✓
Turdus 3	17 days	SkyScan 1173 (BRUKER)	UT (Bunkyo-ku, Tokyo)	800	240	4	14.25	14.25	2240 x 2240	120	66	Brass 0.25mm		
